# The Barley Genome Sequence Assembly Reveals Three Additional Members of the *CslF* (1,3;1,4)-β-Glucan Synthase Gene Family

**DOI:** 10.1371/journal.pone.0090888

**Published:** 2014-03-03

**Authors:** Miriam Schreiber, Frank Wright, Katrin MacKenzie, Pete E. Hedley, Julian G. Schwerdt, Alan Little, Rachel A. Burton, Geoffrey B. Fincher, David Marshall, Robbie Waugh, Claire Halpin

**Affiliations:** 1 Division of Plant Sciences, University of Dundee at The James Hutton Institute, Invergowrie, Dundee, Scotland, United Kingdom; 2 The James Hutton Institute, Invergowrie, Dundee, United Kingdom; 3 Biomathematics and Statistics Scotland (BioSS), Invergowrie, Dundee, United Kingdom; 4 ARC Centre of Excellence in Plant Cell Walls, University of Adelaide, Waite Campus, Glen Osmond, South Australia, Australia; University of Massachusetts Amherst, United States of America

## Abstract

An important component of barley cell walls, particularly in the endosperm, is (1,3;1,4)-β- glucan, a polymer that has proven health benefits in humans and that influences processability in the brewing industry. Genes of the cellulose synthase-like (*Csl*) F gene family have been shown to be involved in (1,3;1,4)-β-glucan synthesis but many aspects of the biosynthesis are still unclear. Examination of the sequence assembly of the barley genome has revealed the presence of an additional three *HvCslF* genes (*HvCslF11*, *HvCslF12* and *HvCslF13*) which may be involved in (1,3;1,4)-β-glucan synthesis. Transcripts of *HvCslF11* and *HvCslF12* mRNA were found in roots and young leaves, respectively. Transient expression of these genes in *Nicotiana benthamiana* resulted in phenotypic changes in the infiltrated leaves, although no authentic (1,3;1,4)-β-glucan was detected. Comparisons of the *CslF* gene families in cereals revealed evidence of intergenic recombination, gene duplications and translocation events. This significant divergence within the gene family might be related to multiple functions of (1,3;1,4)-β-glucans in the Poaceae. Emerging genomic and global expression data for barley and other cereals is a powerful resource for characterising the evolution and dynamics of complete gene families. In the case of the *CslF* gene family, the results will contribute to a more thorough understanding of carbohydrate metabolism in grass cell walls.

## Background

Interest in barley as a food component has been increasing due to the comparatively high levels of mixed linkage (1,3;1,4)-β-glucan found in the grain. In 2006, the U.S. Food and Drug Administration (FDA) approved health-related claims stating that the intake of 3 grams of soluble β-glucan (from oat or barley) per day helps to effectively lower blood total and LDL cholesterol [1], [2]. The (1,3;1,4)-β-glucan functions as soluble dietary fibre and has additional health benefits in reducing the risk of cardiovascular disease (CVD), type II diabetes and colorectal cancer [1], [2]. In the gastrointestinal tract, (1,3;1,4)-β-glucan is believed to form a gel matrix that increases bile acid excretion and delays glucose absorption into the blood, thus lowering insulin levels. The health properties of (1,3;1,4)-β-glucan are thus dependent on its molecular weight (MW) and solubility [3]. However, in the brewing and distilling industries, high levels of (1,3;1,4)-β-glucan are undesirable, causing problems with filtration and decreasing processability. Similarly, (1,3;1,4)-β-glucans are classified as anti-nutrients in animal feed formulations, where they reduce growth rates of monogastric animals [4].

Although commonly found in walls of the graminaceous monocotyledons, (1,3;1,4)-β-glucan is generally absent from dicotyledon cell walls. The polymer is a major constituent of the primary cell wall and more minor component of secondary cell walls in most members of the Poaceae, including the common cereals wheat, barley and oat [5]. In seeds, (1,3;1,4)-β-glucan may play a role in energy storage and it is believed to have a growth-related function in vegetative tissues, although significant levels of (1,3;1,4)-β-glucan also occur in mature tissues of rice and some other grasses [6]. The first functional identification of a gene capable of synthesising (1,3;1,4)-β-glucan came from Burton et al. [7] who transformed the dicot *Arabidopsis thaliana* with a cellulose synthase-like *CslF2* gene from rice (*Oryza sativa*) and demonstrated the subsequent presence of a small amount of (1,3;1,4)-β-glucan in the dicot cell walls. In 2009, Doblin et al. introduced a *CslH* gene from barley into Arabidopsis and this gene also promoted synthesis of detectable amounts of (1,3;1,4)-β-glucan; thus it appears that two different gene families could be involved in the synthesis of the polymer. To date, involvement in (1,3;1,4)-β-glucan synthesis has been demonstrated for the barley proteins CslF4, CslF6 and CslH [8], [9]. The *CslF* and *CslH* genes are members of the superfamily of cellulose synthases (*CesA* genes) and cellulose synthase-like (*Csl*) genes [10]. Further investigations of (1,3;1,4)-β-glucan synthesis and the *CslH1* gene showed that the enzyme, but not the (1,3;1,4)-β-glucan, could be detected in the Golgi apparatus by antibodies. The (1,3;1,4)-β-glucan can only be detected outside the plasma membrane. The hypothesis is that a modification occurs at the plasma membrane, making polymer epitopes accessible to the antibody [7], [9], [11].

Given the emerging importance of the (1,3;1,4)-β-glucan polymer for both human health and industry, it is of interest to understand which of the *Csl* genes have the potential to direct (1,3;1,4)-β-glucan synthesis, where and when they mediate it, and how the polysaccharide is used by the plant during different phases of its life cycle. In this paper we use the new barley genome assembly [12] to re-examine the composition and dynamics of the *HvCslF* gene family from barley, and also perform an initial analysis of the gene family in wheat. Our results provide a platform for understanding the different roles *HvCslF* genes may play in barley growth, development and interaction with its environment.

## Results

### Identification and mapping of barley *CslF* genes including three previously undescribed genes

**Figure 1:**
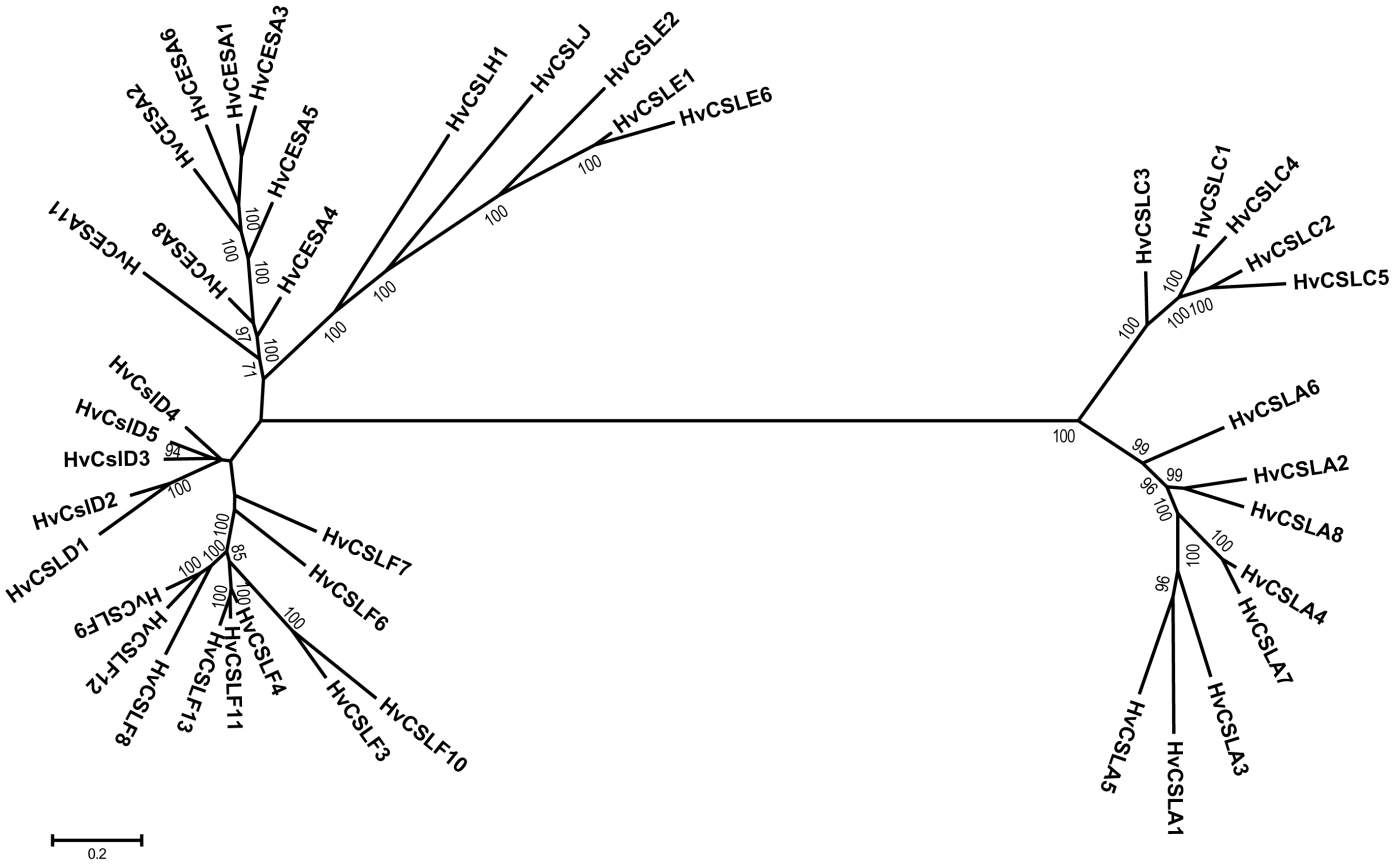
An unrooted phylogenetic tree of the barley *Csl* super family. Phylogenetic analysis was done using MrBayes (codon position model) in TOPALi v2. The posterior probabilities have been multiplied by 100. The scale bar shows expected number of nucleotide substitutions per site.

**Figure 2:**
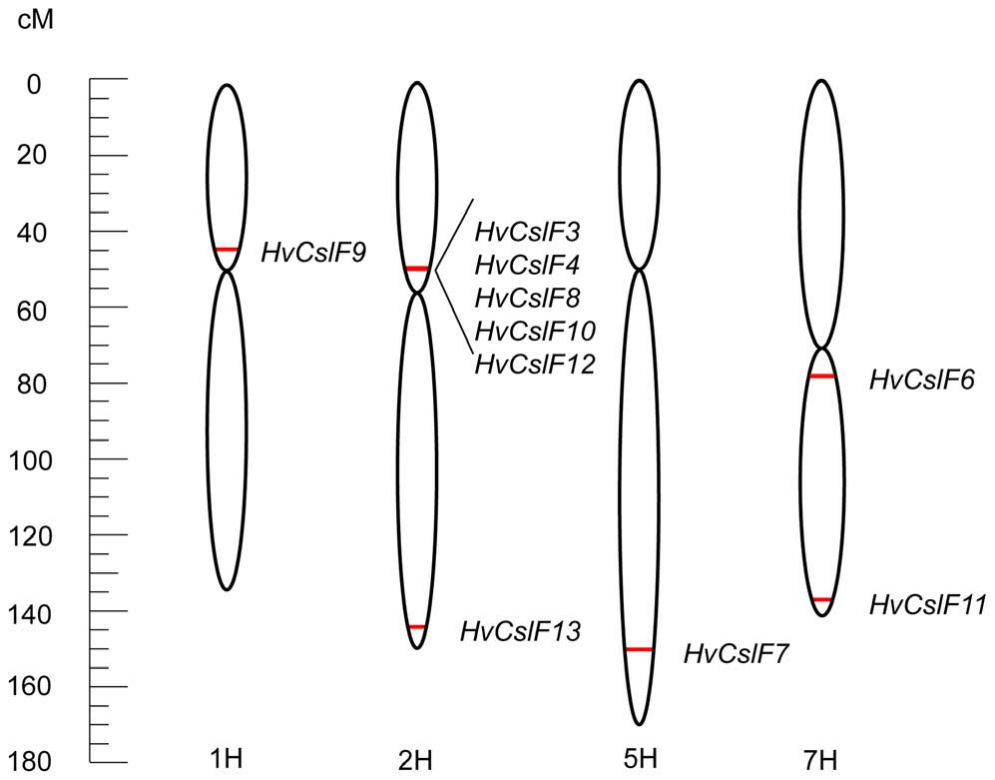
The genetic location of barley *HvCslF* genes. Genetic map of barley chromosomes 1H, 2H, 5H, and 7H showing the positions of barley *HvCslF* genes as mapped in a ‘Morex’ × ‘Barke’ population [12]. cM  =  centimorgan.

Sequences for the seven known barley *CslF* family genes were collected from GenBank (i.e. *HvCslF3*, *HvCslF4*, *HvCslF6*, *HvCslF7*, *HvCslF8*, *HvCslF9*, and *HvCslF10*). These seven genes, along with mutant versions of *CslF6*, are the only barley *CslF* sequences currently listed on the Carbohydrate-Active Enzymes database (www.cazy.org). A BLAST search of the newly available barley cv. Morex sequence assembly [12] resulted in seven sequences identical to those from GenBank together with three new sequences. The *CslF* family members are named after their homologs in rice, starting with *HvCslF3* as there are no homologous sequences in barley to *OsCslF1* and *OsCslF2*. *OsCslF5* is thought to be a pseudogene and there are no homologs in barley. An additional *CslF* gene was previously found in barley compared to rice and named *HvCslF10* [13]. In keeping with this scheme, the three new sequences described here were named *HvCslF11*, *HvCslF12* and *HvCslF13*. A phylogenetic tree (Figure 1) clearly shows that *HvCslF11* and *HvCslF13* are most closely related to *HvCslF4,* while *HvCslF12* is most closely related to *HvCslF9*. The genetic location of all ten barley *CslF* genes was determined from the barley genome assembly [12]. The *HvCslF9* gene is located on the short arm of chromosome 1H, *HvCslF7* is located on the long arm of chromosome 5H and *HvCslF6* is located on the long arm of chromosome 7H (Figure 2). The other members, *HvCslF3*, *HvCslF4*, *HvCslF8* and *HvCslF10*, are localized in a cluster on chromosome 2H near the centromeric region. Of the three new sequences, *HvCslF12* is also located in this cluster on the short arm of chromosome 2H and *HvCslF11* is on the long arm of chromosome 7H. A clear map position was initially not identified for *HvCslF13* but a precise mapping position on the long arm of chromosome 2H was determined by analysis of the results from Mascher et al. [14] (Figure 2).

### Predicted protein structures of the new *CslF* genes

**Figure 3:**
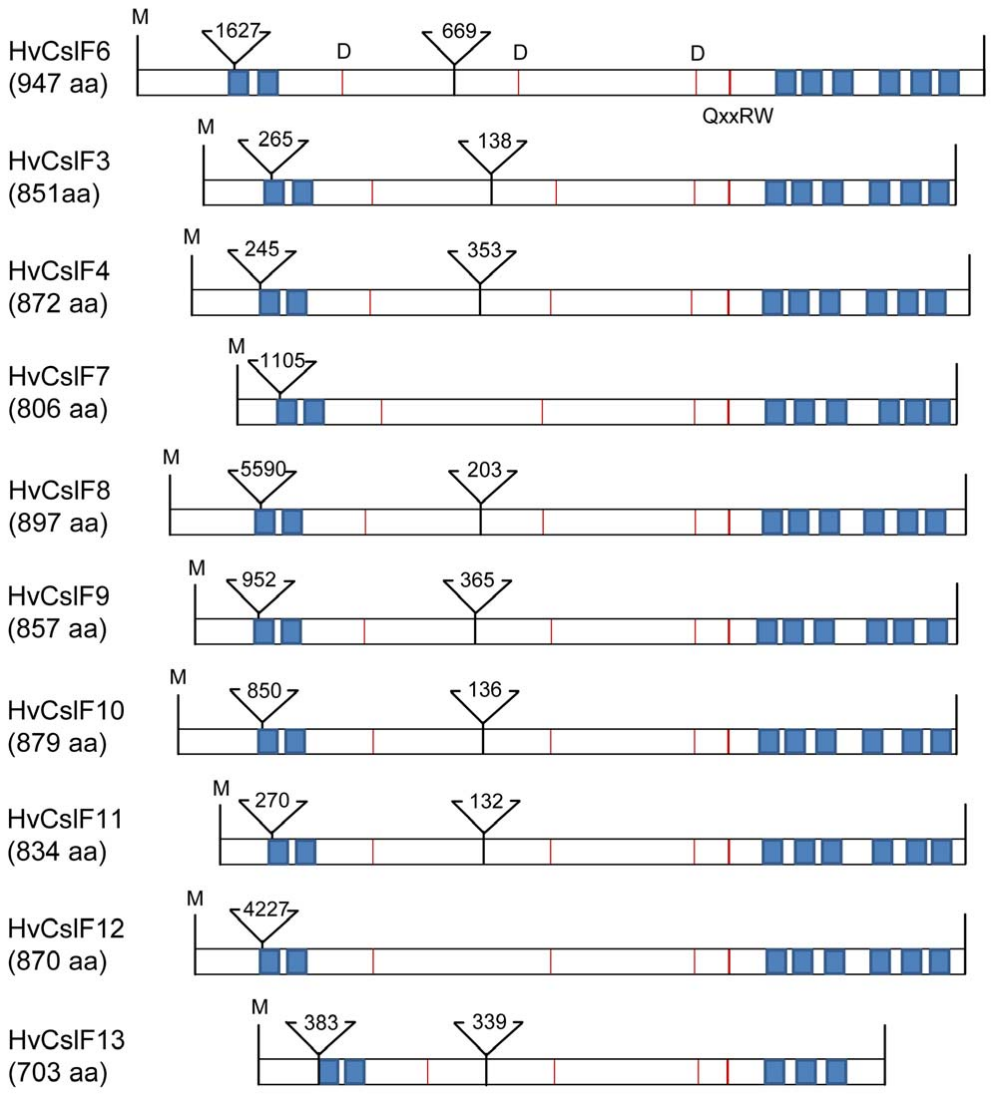
The protein structure of the HvCslF family members. The shaded boxes indicate the positions of sequences encoding trans-membrane helices, which can be found in similar positions in all genes. The triangle marks the intron position with the size given in base pairs. The lines indicate the glycosyltransferase GT2-motif D,D,D,QxxRW.

**Figure 4:**
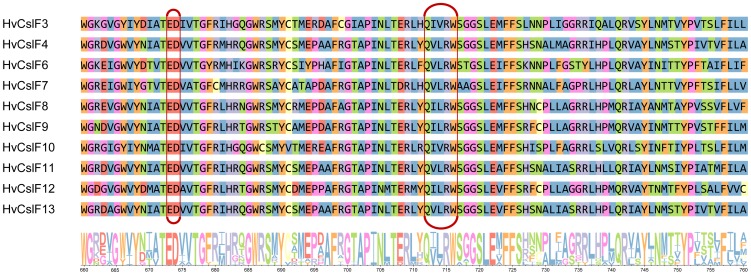
Part of the glycosyltransferase GT2-motif with surrounding amino acid residues. The C-terminal part of the glycosyltransferase GT2-motif, encompassing ED and QxxRW, are marked by the red border. The colour coding is as follows: Aliphatic/hydrophobic: ILVAM (blue), Aromatic: FWY (orange), Positive: KRH (purple), Negative: DE (red), Hydrophilic: STNQ (green), conformationally special: PG (pink), Cysteine: C (yellow).

The predicted protein structures of the newly-identified HvCslF11 and HvCslF12 indicate sizes of 834 and 870 amino acids, respectively, within the range defined by other family members. The full but shorter HvCslF13 protein sequence of 703 amino acids could only be inferred by reference to the cv. Bowman [12] since the cv. Morex sequence contained premature stop codons. All three enzymes have the characteristic glycosyltransferase motif D, D, D, QxxRW and therefore belong to the GT2 family of glycosyltransferases [15]. For all HvCslF family members except HvCslF13, eight trans-membrane helices have been predicted, two near the 5’ end and six near the 3’ end, placing the 5’ end and catalytic motif putatively in the cytosol. An early stop codon in the cv. Bowman *HvCslF13* gene leads to only three trans-membrane helices at the 3’ end; *HvCslF13* is therefore potentially a pseudogene. With the exception of *HvCslF7* and *HvCslF12*, which have a single intron, all other *CslFs* have two introns which vary from 132 to over 5500 base pairs (Figure 3). A closer look at the catalytic motif shows a strongly conserved region. The HvCslF3 and HvCslF10 enzymes have a QIVRW motif, while HvCslF8, HvCslF9 and the newly identified HvCslF12 share a QILRW motif. HvCslF4, HvCslF6, HvCslF7 and the two ‘new’ HvCslF11 and HvCslF13 enzymes have a QVLRW motif. This could be of importance because demonstration of (1,3;1,4)-β-glucan synthesis activity has so far been restricted to HvCslF4 and HvCslF6 [8], [9] i.e. the genes with the QVLRW motif. In the majority of cases, changes in amino acid residues around the motif are conservative (Figure 4). A key distinguishing feature of HvCslF6 is the presence of a 54 amino acid loop in the cytoplasmic region of the enzyme, compared to a loop of only 15–20 residues in the other HvCslF proteins [13] including the ‘new’ HvCslF11, HvCslF12 and HvCslF13.

### Expression profiles of barley *CslF* genes

**Figure 5:**
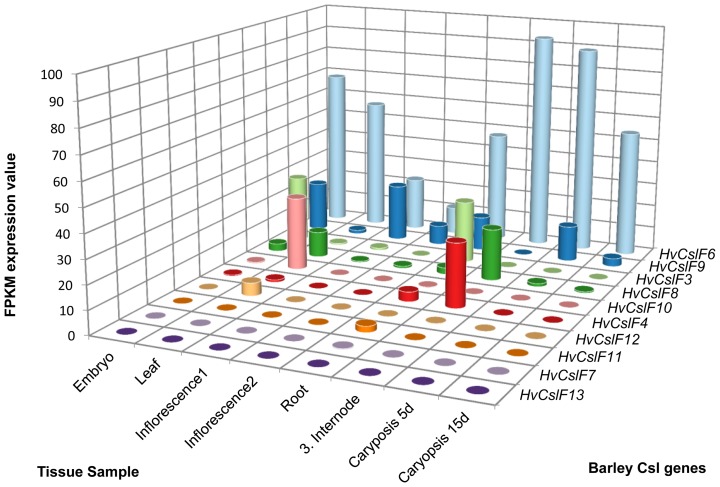
Expression data of the *HvCslF* genes based on RNA-sequence data. The RNA-sequence data for the *HvCslF* genes includes three biological replicates per tissue. The results are given in FPKM expression values (fragments per kilobase of exon per million fragments mapped). Values were obtained from the International Barley Genome Sequencing Consortium [12].

By mining the expression data published by the International Barley Genome Sequencing Consortium [12], some interesting variation between the members of this gene family is observed. In almost every tissue the highest expression is from the *HvCslF6* gene (Figure 5). For *HvCslF7* and *HvCslF13* no expression is detectable, while *HvCslF3*, *HvCslF8* and *HvCslF10* all had distinct expression patterns. The *HvCslF4* gene is transcribed in the third internode and the root. In comparison, the very closely related newly identified gene, *HvCslF11*, is only expressed in root tissue (Figure 5), and this result is validated by microarray analysis on the same tissue samples (Figure S1). Expression of *HvCslF9* can be found in almost every tissue except for the third internode and leaf with the highest expression in the first inflorescence sample. The structurally similar *HvCslF12* mRNA could only be found in the leaf (Figure 5 and Figure S1). The distinct expression patterns make the genes interesting for further analysis.

### Functional characterisation of *HvCslF11* and *HvCslF12* in *Nicotiana benthamiana*

**Figure 6:**
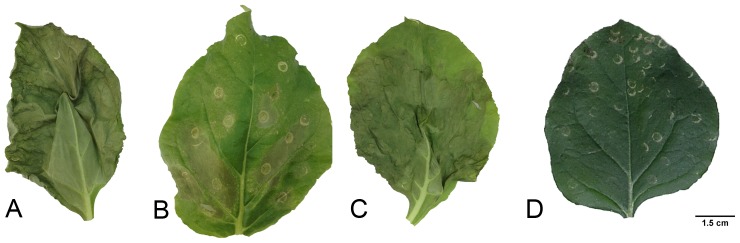
Transient expression of different *HvCslF*-constructs in *Nicotiana benthamiana* leaves results in necrosis. *HvCslF6* (a), *HvCslF11* (b), *HvCslF12* (c) and empty vector (d) constructs were transiently expressed in 4-weeks old *N. benthamiana* leaves using *Agrobacterium tumefaciens* as a vector. Photographs were taken six days after infiltration. Necrosis symptoms were observed for all three constructs.

In order to investigate the ability of the new HvCslF11 and HvCslF12 to synthesize (1,3;1,4)-β-glucan, a *N. benthamiana* transient expression system was used. The suspected pseudogene, *HvCslF13*, which gives rise to a truncated protein is not expected to have activity and was excluded from the analysis. For both genes, binary plasmid constructs were made and infiltrated into *N. benthamiana* leaves using *Agrobacterium tumefaciens* as a vector. After 6 days the leaves were harvested. Control leaves infiltrated with an ‘empty’ vector without *CslF* sequences showed no necrosis whereas leaves infiltrated with HvCslF11, HvCslF12 and HvCslF6 (positive control) showed unusual medium to strong necrosis (Figure 6). The leaves were analysed for (1,3;1,4)-β-glucan using a lichenase digestion which results mainly in tri-saccharide (DP3) and tetra-saccharide (DP4) hydrolysis products that can be analysed on a Dionex HPAEC column. HvCslF6 infiltrated leaves had a DP3:DP4 ratio of 1.6 but no DP3 and DP4 peaks could be detected for the HvCslF11 or HvCslF12 infiltrated leaves, despite the unusual phenotype (Table S1).

### Evolutionary analysis

Determining the relationships among the sequences of the *CslF* genes in different cereals can deepen our understanding of the evolutionary history of the individual genes. To put the relationship between the *HvCslF* genes into a wider evolutionary context, the *CslF* genes of barley (*Hordeum vulgare*), rice (*Oryza sativa*), sorghum (*Sorghum bicolor*), Brachypodium (*Brachypodium distachyon*) and wheat (*Triticum aestivum*) were compared. Our analyses identified eight members of the *CslF* family in rice, seven in Brachypodium, ten in sorghum and ten in barley. Searching the International Wheat Genome Sequencing Consortium (IWGSC) [16] database on the Unité de Recherche Génomique Info (URGI) [17] website revealed 34 *TaCslF* sequences in hexaploid wheat, although this may not be the complete gene family.

**Figure 7:**
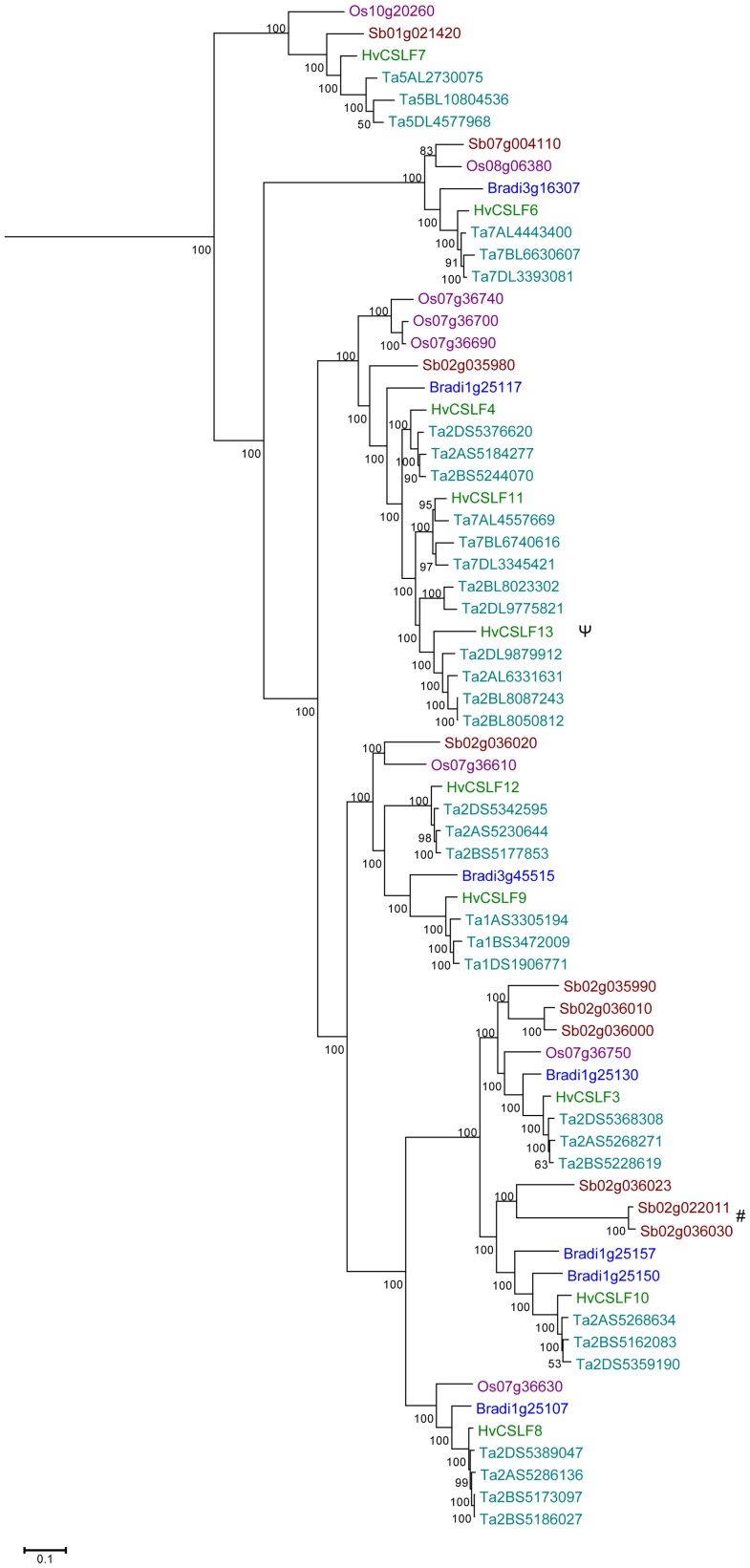
Phylogenetic tree of the *CslF* gene family including five cereals. Phylogenetic tree of 69 members of the *CslF* gene family including wheat, barley, Brachypodium, rice and sorghum. The tree was constructed with TOPALi v2 on a subset of the genes using MrBayes (codon position model). The posterior probabilities have been multiplied by 100. An outgroup (not shown) comprised 10 *CslH*/*CslJ* sequences. *HvCslF13* is marked as a potential pseudogene ψ. The two potential mosaic sequences Sb02g022011 and Sb02g036030 are marked by #. The scale bar shows expected number of nucleotide substitutions per site.

We performed Bayesian phylogenetic analysis using MrBayes from the TOPALi package [18] with the predicted coding sequences of all 69 *CslF* genes (plus an outgroup, not shown, of 10 *CslH*/*CslJ* genes). The resulting phylogenetic tree shows a clear division into different clades (Figure 7) and highlights several duplication events occurring in different cereals. For example, the (*CslF4 (CslF11, CslF13)*) clade reveals evidence of two duplication events in the rice lineage (*OsCslF1* [Os07g36700**],** *OsCslF2* [Os07g36690**]**), plus two duplication events barley (*CslF11*, *CslF13*) and possibly three in wheat, all independent from the rice duplication. Further duplication events in the *CslF9* gene clade appear to have led to the origin of the ‘new’ *HvCslF12* gene and the same duplication is also present in wheat. In the *CslF3* gene clade a duplication event in sorghum can be inferred, and the nonexistence of a *CslF10* gene in rice suggests a loss of this gene. Checking the coding sequences for evidence of past recombination using the NeighborNet method in the SplitsTree program [19] suggested that two sequences in sorghum (Sb02g022011 and Sb02g036030), assigned to the *CslF10* clade, appear to be mosaic sequences [Schreiber M, Wright F and MacKenzie K, unpublished observation] consisting of part of a *CslF10* gene and part of a *CslF9* gene.

An analysis of natural selection among clades was performed using the branch model in the PAML package [20]. It is likely that the duplicated genes evolve in distinct ways due to exposure to different types of selection pressure that can be described with the nonsynonymous/synonymous ratio (Ka/Ks). We found significant differences in the Ka/Ks ratio among clades with the ratio varying from 0.0398 to 0.1944 among the ten clades (see Methods). The lowest Ka/Ks ratios were found in the CslF6 clade (Ka/Ks  =  0.0398) and the highest in the CslF7 and CslF10 clades (Ka/Ks  =  0.1953 and 0.1944 respectively). The relative Ka/Ks ratios observed after putative duplication events in the CslF12 and CslF9 clades (Ka/Ks  =  0.1308 and 0.0780 respectively) suggested that these genes are not under positive selection pressure, but under purifying selection.

## Discussion

High levels of (1,3;1,4)-β-glucan in some cereal grains have important positive implications for human health, while low levels are necessary for processability in the alcoholic beverages and animal feed industry. Knowing what genes are involved in (1,3;1,4)-β-glucan synthesis and breakdown, and when and where they are switched on or off is therefore important to understanding these contrasting features. The discovery of three ‘new’ *HvCslF* genes in barley is relevant to ongoing studies of the dynamics of cell wall synthesis in the grasses. Our data are consistent with *HvCslF13* being either non-functional or a pseudogene. However, all three newly discovered *HvCslFs* possess the important glycosyltransferase GT2-motif, and *HvCslF11* and *HvCslF12* are expressed, albeit restricted to root and leaf respectively. These data suggests that *HvCslF11* and *HvCslF12* could play a role in (1,3;1,4)-β-glucan biosynthesis in roots or leaves but their role, if any, in grain tissues would likely be minor. Transient expression of *HvCslF11* and *HvCslF12* in *N. benthamiana* revealed no evidence for authentic (1,3;1,4)-β-glucan biosynthesis. Nevertheless, a shrivelled necrotic phenotype was observed that was not due to infiltration damage. The HPAEC profiles suggested that some new molecules were being produced, but they could not be identified. It is possible that the expressed genes are producing some unusual polysaccharide product and that this is causing the phenotype. The *N. benthamiana* transient system has been moderately successful for testing (1,3;1,4)-β-glucan synthase activity, producing small amounts of the polymer on expression of *HvCslF4*, *HvCslF6* or *HvCslH* [Little A, Burton RA, Fincher GB, unpublished observation]. It may be that other components that are necessary for efficient (1,3;1,4)-β-glucan synthesis are missing in the dicot cells and remain to be identified. Consequently, the fact that *HvCslF11* and *HvCslF12* cannot synthesize (1,3;1,4)-β-glucan on their own in dicot cells does not exclude them from being involved in (1,3;1,4)-β-glucan synthesis in barley.

The first connection between grain (1,3;1,4)-β-glucan content and the *CslF* genes emerged from a genetic study on QTL (Quantitative Trait Loci) that affect grain (1,3;1,4)-β-glucan content [21]. While this study identified four QTL, Burton et al. [7] used conservation of synteny around the major QTL on chromosome 2H to identify the orthologous cluster of *CslF* genes on rice chromosome Os07. They then showed using stable expression that two genes in the cluster, *OsCslF2* and *OsCslF4*, were able to synthesise (1,3;1,4)-β-glucan in Arabidopsis. An intriguing observation about these QTL studies is that the genetic analyses of [21] used a population derived from a cross between the barley cultivars Steptoe and Morex. The expression studies we report here used RNA-seq. data from the cv. Morex. Importantly in our datasets, none of the 2H *CslF* cluster genes are expressed in the developing grain suggesting they may not be involved in synthesising grain (1,3;1,4)-β-glucan. This conclusion is supported by observations that when *CslF6* on chromosome 7H is mutated, no detectable (1,3;1,4)-β-glucan is found in the leaf or in the grain [22], indicating *CslF6* as a major gene responsible for grain (1,3;1,4)-β-glucan biosynthesis. However, this apparent absence of (1,3;1,4)-β-glucan in the *HvCslF6* mutant must be reconciled with our observations (Figure 5) and those of Burton et al. [13], that transcripts of both *HvCslF9* and *HvCslF8* are present in developing grain, albeit at lower levels than *HvCslF6* transcripts. The *HvCslF8* gene is a member of the cluster on chromosome 2H. Furthermore, our transcript profiles here are limited to 15 days post pollination, and we have found in other studies that *HvCslF* gene transcription can be initiated as late as 30-35 days post pollination [Wong SC, Mather DE, Burton RA, Fincher GB, unpublished observation]. At this stage, the level of involvement of the *HvCslF* genes in the chromosome 2H cluster in (1,3;1,4)-β-glucan synthesis remains unclear. Similarly, the final levels of (1,3;1,4)-β-glucan in mature barley grain may also be controlled by (1,3;1,4)-β-glucan endohydrolases for which high levels of gene transcripts are sometimes detectable in developing grain [13].

The solubility of (1,3;1,4)-β-glucan is believed to be affected by changes in the DP3:DP4 ratio which could influence other (1,3;1,4)-β-glucan properties. *HvCslF6* and *HvCslF4* have been shown to alter the DP3:DP4 ratio when overexpressed in barley [8]. *HvCslF11* is very closely related to *HvCslF4* and therefore could have an influence on (1,3;1,4)-β-glucan characteristics of roots, where *HvCslF11* is specifically expressed. There has been no study so far to our knowledge that has examined the importance of (1,3;1,4)-β-glucan in the roots.

Using synteny (Figure S2) between several members of the grass family, and combining this information with phylogenetic analyses between the *CslF* genes allowed us to better understand the evolution of this gene family. Originating from a common ancestor, sorghum was the first to diverge, followed by rice and then Brachypodium [23]. Sorghum has *SbCslF7* (Sb01g021420) on chromosome Sb01, *SbCslF6* (Sb07g004110**)** on chromosome Sb07 and a cluster of *CslFs* on chromosome Sb02. These genes exhibit conserved synteny with their likely orthologs in rice, but different duplication events characterise the cluster. The sequence from *SbCslF3* (Sb02g035990**)** is duplicated twice (Sb02g036010, Sb02g036000**)** and there is a recombination event between two sequences (Sb02g022011, Sb02g036030**)** which are assigned to the *SbCslF10* clade. One of these sequences (Sb02g022011**)** appears to have been translocated to a more distal position, further from the centromere, on the chromosome Sb02 (Figure 7). Rice has one cluster of *CslF*s on chromosome Os07, and two outliers with *OsCslF6* (Os08g06380**)** on chromosome Os08 and *OsCslF7* (Os10g20260**)** on chromosome Os10. Based on homology one would expect to find *HvCslF9*, *HvCslF11* and *HvCslF13* as part of the cluster of genes located on barley chromosome 2H, instead of chromosomes 1H, 7H and 2H respectively. The *HvCslF11* and *HvCslF13* genes appear to have resulted from duplication followed by translocation. The wheat homologs of *HvCslF9*, *HvCslF11* and *HvCslF13* are present in all three genomes on chromosomes 1ABD, 7ABD and 2ABD, but they are not found in Brachypodium. The duplication and translocation must therefore have happened after the separation from Brachypodium. Wheat also appears to have duplicated a gene closely related to *CslF13* and *CslF11* after the separation from barley (Figure 7). Brachypodium shows a different pattern with the cluster on chromosome Bd01, and *BdCslF6* (Bradi3g16307**)** on chromosome Bd03, while *BdCslF7* is lost. *BdCslF9* (Bradi3g45515**)** in Brachypodium is an outlier as in barley, but synteny is not conserved with either rice or barley. The different composition of the *CslF* genes, their locations in the different grass species and patterns of gene expression could help us understand how they evolved and how they influence (1,3;1,4)-β-glucan content and function at different times and in different tissues in related species. Why, for example does rice have little or no (1,3;1,4)-β-glucan in the grain, whereas the (1,3;1,4)-β-glucan content in Brachypodium grain is up to 40% w/w and barley shows a moderate amount of (1,3;1,4)-β-glucan with 4-10% w/w [5], [24]? Is this due to selection for starchy grains during domestication? It is clear that the (1,3;1,4)-β-glucan in the grain of Brachypodium largely replaces starch as the primary storage carbohydrate, consistent with suggestions that (1,3;1,4)-β-glucan acts as an alternative source of metabolizable glucose in leaves of young barley seedlings [25]. Thus, (1,3;1,4)-β-glucans in the Poaceae may play several functional roles in cell walls and in plant energy biology.

## Conclusions

We have characterised here three newly identified *CslF* genes in barley that do not appear in rice, Brachypodium or sorghum, but are present in wheat. While their involvement in (1,3;1,4)-β-glucan synthesis has yet to be proven, at least two are expressed specifically in leaf and root tissues. Emerging genomic data for barley and related grass species is a powerful resource for characterising the evolution and dynamics of the complex *CslF* gene family. This will, in the longer term, contribute to a more thorough understanding of the mechanisms and processes regulating complex carbohydrate metabolism in grass cell walls.

## Methods

### Sequence data, databases and preprocessing

The available *HvCslF* gene sequences were collected from the National Center for Biotechnology Information (NCBI) [*HvCslF3*, GenBank: EU267179; *HvCslF4*, GenBank: EU267180; *HvCslF6*, GenBank: EU267181; *HvCslF7*, GenBank: EU267182; *HvCslF8*, GenBank: EU267183; *HvCslF9*, GenBank: EU267184; and *HvCslF10*, GenBank: EU267185], (http://www.ncbi.nlm.nih.gov/). These sequences were used for a BLAST [26] search on the Barley WGS Morex Assembly version3 [International Barley Genome Sequencing 12]. The deep-sequencing dataset is available for download from: http://mips.helmholtz-muenchen.de/plant/barley/index.jsp or is available for a BLAST search on: http://webblast.ipk-gatersleben.de/barley/. The accession numbers for the *CslF* gene sequences are included (*HvCslF3*: MLOC_59289, *HvCslF4*: MLOC_74149, *HvCslF6*: MLOC_57200, *HvCslF7*: MLOC_51212, *HvCslF8*: MLOC_52692, *HvCslF9*: MLOC_59327, *HvCslF10*: MLOC_13463, *HvCslF11*: MLOC_19594, *HvCslF12*: MLOC_7825). Additionally, the MSU Rice Genome Annotation Project (http://rice.plantbiology.msu.edu/) was used to obtain the *CslF* genes from rice and a BLAST search was conducted on the Barley WGS Morex Assembly to search for further sequences. These sequences from rice and barley were used to conduct a BLAST search for the *CslF* genes from sorghum (http://mips.helmholtz-muenchen.de/plant/sorghum/) [27], Brachypodium (http://mips.helmholtz-muenchen.de/plant/brachypodium/) [28] and wheat (IWGSC, URGI) [16], [17]. The default settings of the respective websites were used to conduct the BLAST search. The alignment was then checked by eye and the sequences were validated by a reciprocal BLAST search to the rice genome. The identified coding sequences (excluding wheat due to pre-publication access) are given in Dataset S1.

### Multiple alignment and Phylogenetic/evolutionary analyses

The above-mentioned 79 protein sequences were aligned using MUSCLE within MEGA5 [29] and from this a 4323bp-long codon alignment was created by replacing the amino acids with codons and single amino acid gaps with codon-sized gaps. Unreliable alignment positions were then removed using the BMGE method [30] resulting in an 1845bp alignment. The subsequent model selection and phylogenetic analysis took into account codon structure by having a nucleotide substitution model for each codon position: this “codon position” model thus consists of three models. The choice of model at each codon position was optimised using the TOPALi v2 [18] model selection method and the models chosen were GTR+I+G for the first and third positions and GTR+G for the second position. This model was then used to estimate a Bayesian phylogenetic tree using MrBayes v3.1.1 [31] launched from TOPALi v2. The Bayesian analysis settings were 2 runs of 625,000 generations, a 25% burn-in with trees sampled every 10 generations, resulting in 100,000 trees from two independent runs. The potential scale reduction factor (PRSF) values of all parameters were less than 1.06 (95% had values < 1.03) suggesting good convergence (i.e. less than a PRSF threshold of 1.2 as suggested by Gelman et al. [32]) of the two runs. The tree was rooted with ten sequences from the *CslH* and *CslJ* clusters (not shown in Figure 7). The posterior probabilities that show support for each cluster have been converted into percentages by MEGA5 during the production of the tree diagram shown in Figure 7.

Visual checking for evidence of recombination was done using the default analysis, NeighborNet, in the SPLITSTREE package [19]. When the NeighborNet phylogenetic network suggested that certain sequences were mosaic sequences, the analysis was rerun excluding them to see if the recombination signal was still present. By running NeighborNet interactively excluding alignment regions, the putative origin of regions in mosaic sequences was investigated. Phylogenetic trees were also estimated, using PhyML within TOPALi, from regions on each side of a putative recombination breakpoint to assist in determining the likely origin of regions in the mosaic sequences.

The PAML package [20] was used to investigate the variation in Ka/Ks ratios among clades using a likelihood ratio test for variation in selective pressure among branches in a gene tree based on the Yang and Bielawski protocol [33]. We found significant differences in the Ka/Ks ratio among clades by testing a null hypothesis, H_0_, that Ka/Ks was the same in all clades (Ka/Ks equal to 0.1329) versus the alternative hypothesis, H_1_, that Ka/Ks varied among the clades (Log likelihoods of -46465.1 and -46669.5, respectively for H_0_ and H_1,_ were used to produce a Likelihood Ratio test statistic of 408.4 which was significant at p<0.001). The Ka/Ks ratio varied from 0.0398 to 0.1944 among the ten clades with most clades in the range (0.075, 0.150).

### Genetic location and RNA-sequence experiments

Information on the genetic location of the genes and expression data is provided by [12]. For the genetic location over 3.90 gigabases of sequence contigs were anchored to a consensus genetic map based on the analysis and integration of maps from a number of populations, the largest contributor being a recombinant inbred line population derived from a cross between the cultivars Morex and Barke. Eight tissues from cultivar Morex were subjected to RNA sequencing with three replications per tissue [12]. These eight tissues were: germinated embryo (four days after germination), young leaf tissue (from a 10 cm high plant), young root tissue (from a 10 cm high plant), developing inflorescence (5 mm-long inflorescence and 10–15 mm-long inflorescence), the third internode (42-day-old plants) and two time points for the developing caryopsis (five days after anthesis and 15 days after anthesis). The data are presented in FPKM expression values (fragments per kilobase of exon per million fragments mapped).

### Protein prediction

The intron prediction was conducted using Softberry (FGENESH**,** HMM-based gene structure prediction**,** http://linux1.softberry.com/berry.phtml). The result was then confirmed using the RNA-seq. data, if available. Transmembrane helices were predicted using the following websites http://topcons.cbr.su.se/ and http://www.cbs.dtu.dk/services/TMHMM-2.0/ and taking a consensus of both predictions.

### Transient *Nicotiana benthamiana* expression system

Full length *HvCslF11* and *HvCslF12* cDNAs were amplified from Morex, root and young leaf tissue (10 days old), respectively. The following primer pairs were used: HvCslF11_F - AGCCACGGTTTACAGTACGA; HvCslF11_R - ACTACGTACGTGTCTATCCAGA; HvCslF12_F - GAAGAGCCAATGGTTTCGC; HvCslF12_R - CCAGAGAAACGGCATCATCC. The genes were cloned into the Gateway entry vector pCR8/GW/TOPO (Invitrogen, Carlsbad, CA, USA) and sequenced on an ABI 3700 (Applied Biosystems Inc., Foster City, CA, USA) at the Australian Genome Research Facility, Adelaide, Australia, to eliminate constructs with errors. In a LR recombination reaction the inserts were transferred into the Gateway destination vector pEAQ-HT-Dest1 under the control of a CaMW 35S promoter [34]. As a positive control HvCslF6 was included and as a negative control the vector pEAQ-HT-Dest1 without the chloramphenicol resistance gene and ccdB gene was used. The constructs were transformed into the *Agrobacterium tumefaciens* strain AGL1 and left to grow overnight at 28°C in LB medium containing rifampicin and kanamycin. 2 mL of the overnight culture were spread on a LB plate and grown for 2 days at 28°C. 10 mL of an infiltration buffer (10 mM MgCl_2_, 10 mM MES (2-(N-morpholino) ethanesulfonic acid)) were added per plate and cells scraped off the surface. OD_600_ was measured and adjusted to an infiltration OD of 1. 1 µL of 100 mM Acetosyringone was added per mL and left for 3 hours at room temperature. *Nicotiana benthamiana* seedlings were grown under glasshouse conditions, 22°C with natural light, in the Plant Accelerator (University of Adelaide). Whole leaves of 4 week old *Nictotiana benthamiana* plants were infiltrated from the underside using a 10 mL syringe without a needle. Leaves were harvested after 6 days, freeze-dried and ground using a ball bearing mill. Analysis of (1,3;1,4)-β-D-glucan was performed using 20 mg of ground tissue following the commercially available reagents (Megazyme International Ireland Ltd, Bray, Ireland) and a protocol based on [35]. Method modifications include two washes of 50% ethanol and two washes of 100% ethanol for 10 minutes at 97°C, followed by a 20 minute extraction at 90°C in 1 ml 20 mM sodium phosphate buffer (pH 6.5) and a 1.5 hour incubation at 50°C with 40 µL U/ml Lichenase. Total beta glucan levels within the samples were analysed using the glucose oxidase-peroxidase reagent supplied with the kit. DP3:DP4 levels were analysed using HPAEC according to [8] with samples collected following Lichenase digestion.

## Supporting Information

Figure S1 **Microarray validation of RNA seq expression pattern of** ***HvCslF11*** **and** ***HvCslF12.*** Microarray processing was performed on aliquots of identical RNA samples used for the RNAseq (IBGSC, 2012 [11]), using a custom-designed barley Agilent microarray (A-MEXP-2357; www.ebi.ac.uk/arrayexpress). The barley microarray contains c. 61,000 barley 60-mer probes derived from predicted barley transcripts and full-length cDNAs (IBGSC, 2012 [11]). Processing was performed according to the ‘One-Color Microarray-Based Gene Expression Analysis’ protocol (v. 6.5; Agilent Technologies). Data were extracted using Feature Extraction (FE) software (v. 10.7.3.1; Agilent Technologies) with default settings, and subsequently analysed using GeneSpring GX (v. 7.3; Agilent Technologies) software. Data were normalised using default Agilent FE one-colour settings in GeneSpring.
(TIF)

Figure S2 **Chromosome position of the CslF family members highlights synteny between sorghum, rice, Brachypodium and barley.** The Figure was created using Strudel (see Bayer M, Milne I, Stephen G, Shaw P, Cardle L, et al. (2011) Comparative visualization of genetic and physical maps with Strudel. Bioinformatics 27: 1307-1308.).
(TIF)

Table S1 **β-glucan content and DP3:DP4 ratio of** ***CslF*** **gene constructs in the** ***N. benthamiana*** **transient expression system.** MLG  =  mixed linkage glucan; nd  =  not detected.
(DOCX)

Dataset S1 **Coding sequences of the identified** ***CslF*** **genes.**
(DOCX)
