# Supplementary figures and images for: The Barley Genome Sequence Assembly Reveals Three Additional Members of the CslF (1,3;1,4)-β-Glucan Synthase Gene Family
Source: PLoS One. 2014 Mar 3;9(3):e90888. doi: 10.1371/journal.pone.0090888 (PMC3940952; doi:10.1371/journal.pone.0090888)

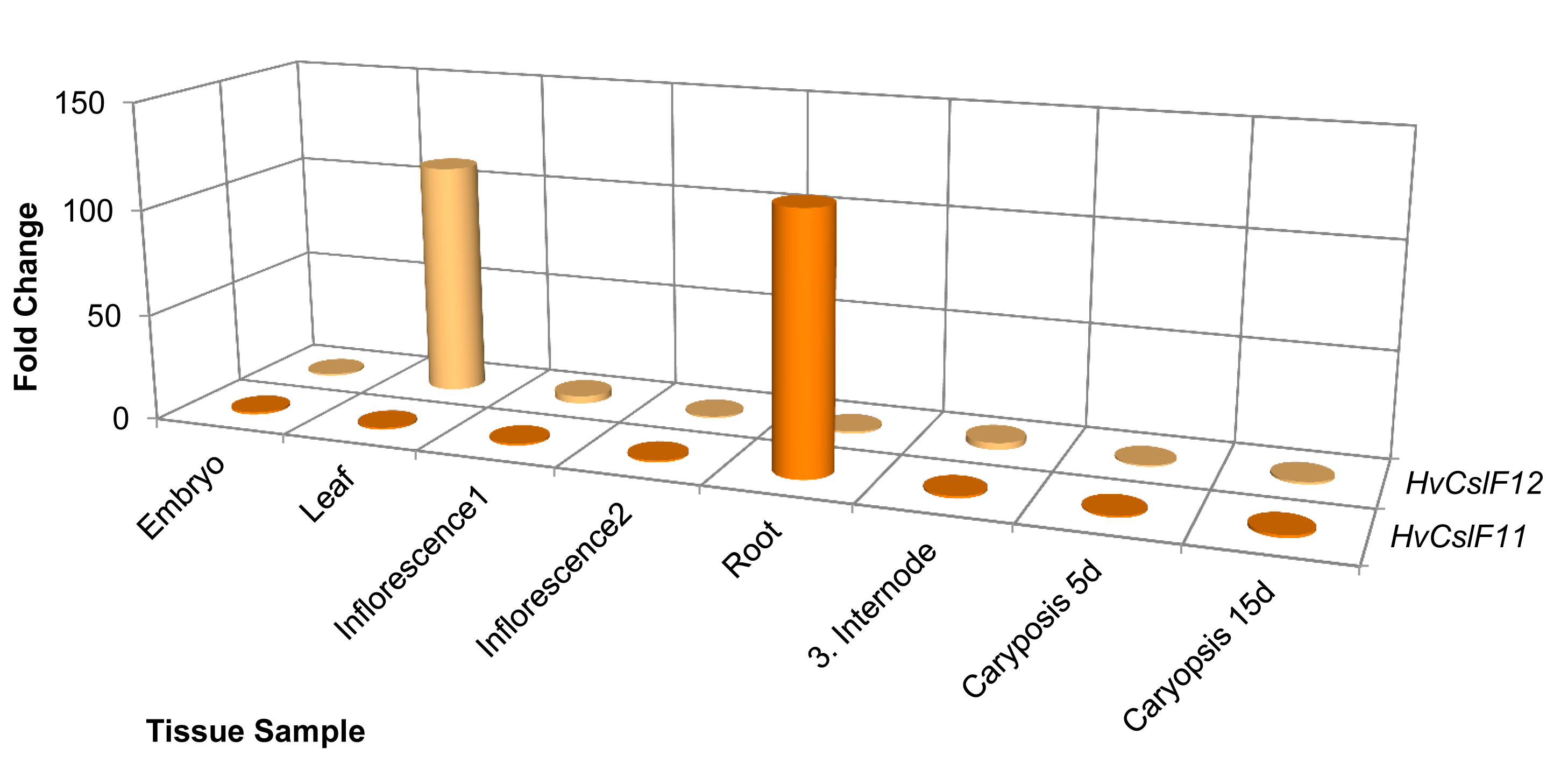

Supplement: Figure S1 — Microarray validation of RNA seq expression pattern of HvCslF11 and HvCslF12. Microarray processing was performed on aliquots of identical RNA samples used for the RNAseq (IBGSC, 2012 [11]), using a custom-designed barley Agilent microarray (A-MEXP-2357; www.ebi.ac.uk/arrayexpress). The barley microarray contains c. 61,000 barley 60-mer probes derived from predicted barley transcripts and full-length cDNAs (IBGSC, 2012 [11]). Processing was performed according to the ‘One-Color Microarray-Based Gene Expression Analysis’ protocol (v. 6.5; Agilent Technologies). Data were extracted using Feature Extraction (FE) software (v. 10.7.3.1; Agilent Technologies) with default settings, and subsequently analysed using GeneSpring GX (v. 7.3; Agilent Technologies) software. Data were normalised using default Agilent FE one-colour settings in GeneSpring. (TIF) [file pone.0090888.s001.tif]

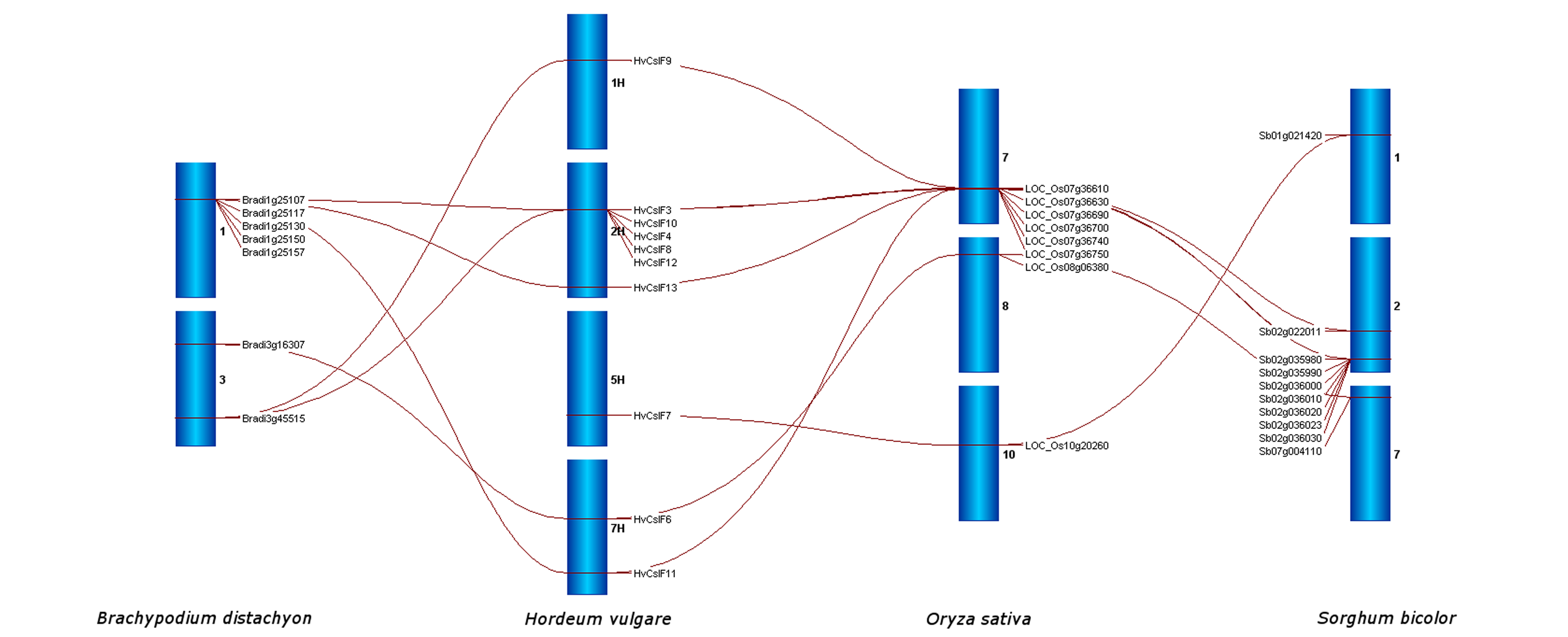

Supplement: Figure S2 — Chromosome position of the CslF family members highlights synteny between sorghum, rice, Brachypodium and barley. The Figure was created using Strudel (see Bayer M, Milne I, Stephen G, Shaw P, Cardle L, et al. (2011) Comparative visualization of genetic and physical maps with Strudel. Bioinformatics 27: 1307-1308.). (TIF) [file pone.0090888.s002.tif]
