# Supplementary material for: The Barley Genome Sequence Assembly Reveals Three Additional Members of the CslF (1,3;1,4)-β-Glucan Synthase Gene Family
Source: PLoS One. 2014 Mar 3;9(3):e90888. doi: 10.1371/journal.pone.0090888 (PMC3940952; doi:10.1371/journal.pone.0090888)
Supplement: Table S1 — β-glucan content and DP3:DP4 ratio of CslF gene constructs in the N. benthamiana transient expression system. MLG = mixed linkage glucan; nd = not detected. (DOCX) [file pone.0090888.s003.docx]

**Table S1.** **The β-glucan content and DP3:DP4 ratio of CslF gene constructs and empty vector control in the *N. benthamiana* transient expression system**

|  | % MLG w/w | StDev | DP3:DP4  where MLG detected | StDev |
| --- | --- | --- | --- | --- |
| empty vector | 0.14 | 0.09 | nd | nd |
| HvCslF6 | 1.56 | 0.12 | 1.66 | 0.01 |
| HvCslF11 | 0.04 | 0.02 | nd | nd |
| HvCslF12 | 0.07 | 0.04 | nd | nd |

MLG = mixed linkage glucan; nd = not detected.
