# Supplementary material for: The Barley Genome Sequence Assembly Reveals Three Additional Members of the CslF (1,3;1,4)-β-Glucan Synthase Gene Family
Source: PLoS One. 2014 Mar 3;9(3):e90888. doi: 10.1371/journal.pone.0090888 (PMC3940952; doi:10.1371/journal.pone.0090888)
Supplement: Dataset S1 — Coding sequences of the identified CslF genes. (DOCX) [file pone.0090888.s004.docx]

>Bradi1g25107

ATGGGTTCTCTGGAGGCAGCGAGCGGTGCTGCCGGCAATGGCGGCGGCGTCGCGGACCAGGCGCTGGCATTGGAGATCGGTGCTGGCATTAATGGGCAGCAGCACAAGGCCGCAGCTGCCGTCGTCGCCCGTGCGCCCGTGCTGGCGAACGGCGGCGCCGGTGGAAAGGCTACGAAGAAGAAGATCAGCCCCAAGGACAGGTATTGGGTGGCTGCCGATGAGGGGGAGATGGAGGCGGCCACGGCGGACGGCGGCGAGGACGGCTTGAGGCCGCTGCTGTACCGGAACTTCAGGGTCAGAGGCATCCTCCTCCATCCATACAGGTTGCTGAGCTTGGTCAGATTGGTTGCTATCGTCCTATTTTTCGTATGGCGTGTCAGGCATCCATATGCAGACGGCATGTGGCTTTGGTGGATATCTATGGTTGGAGATCTTTGGTTTGGTGTCACTTGGTTGCTAAACCAAGTTGCAAAGCTTAATCCTATCAAGCGTGTCCCCAACCTTGCCCTCTTGAAACAGCAGTTTGATCTCCCTGACGGCAACTCCAACCTCCCTCTCCTTGATGTGTTCATCAACACAGTGGATCCCATAAATGAGCCTATGATATACACTATGAACTCCATCTTATCCATTCTTGCCGCAGACTATCCAGTTGACAAGCATGCTTGCTACCTCTCAGATGATGGTGGGTCAATAATCCACTACGATGGGTTGCTTGAGACTGCAAAATTTGCTGCGTTGTGGGTTCCCTTTTGCCGAAAACATTCTATTGAGCCAAGAGCCCCTGAGAGCTACTTTTCTGTAAAGACACGCCCATACACTGGAAATGCTCCAGAAGAGTTTGTCAACGACCACAGACACATGTCTAGAGAGTATGATGAGTTCAAGGGGCATTTAGATGCACTTTTTACCGTCATTCCCCAACGGTCAGATAAATACAATCATGCAGATGCCAAAGAAGGTGCAAAGGCAACTTGGATGGCAGATGGGAAACAGTGGCCAGGCACATGGATCGACCCAGCTGAGAACCATAAGAAAGGACAACATGACGGAATTGTCCAGGTTATGTTGAAACATCCAAGTTATGAACCAGAACTTGGTTTACCTGCAAGCGCCAACAACCCTCTAGACTTCAGCGCCGTTGATGTACGCCTCCCAATGCTTGTGTACATCTCGCGTGAGAAGCATCCAAACTATGATCATCAAAAGAAGGCAGGTGCCATGAACGTACAGCTGCGAGTCTCTGCCCTCCTGACCAATGCGCCCTTCATCATCAACTTCGATGGCGACCACTACGTCAACAACTCAAAAGCTTTCCGGGCTGGCATCTGTTTCATGCTCGACCGCCGTGACGGTGACAATACTGCCTTTGTACAGTTCCCTCAACGCTTTGATGATGTTGATCCTACAGACAGGTATTGCAATCACAACCGTGTCTTCTTCGATGCCACCTTGCTCGGCCTCAACGGCATCCAAGGGCCTTCTTACGTCGGCACTGGTTGCATGTTCCGTCGTGTCTCACTTTATGGTGTTGACCCACCTCGCTGGAGACCTGATGACGCCATGATTGTGGACAGCTCCAACAAGTTTGGCAGTTCACTGTCCTTTATCAGCTCAATGCAGCCAGCCGCAAACCAATCACGCTCGATCATGTCACTGCTTGCACTTGAAGAGTCTGTCATGGCGGAGTTAGCTGATGTCATGAAATGTGCATATGAGGACGGGACTGAGTGGGGCAAGGAAGTTGGCTGGGTGTACAACATTGCAACAGAGGATGTGGTCACCGGCTTCCGCCTGCACCGGAATGGGTGGCGTTCAATGTACTGCCGCATGGAGCCAGATGCATTTGCCGGCACTGCACCTATCAACCTCACTGAGCGCCTCTACCAGATCCTGCGCTGGTCAGGGGGCTCCCTTGAGATGTTCTTCTCACGCAACTGCCCACTTCTGGCCGGTCGCCGACTCCACCCTATGCAACGAATAGCTTACGCCAACATGACGGCCTACCCAGTCTCATCGGTCTTTCTTGTGTTCTATCTCCTCTTCCCGGTTATATGGATCTTCCGTGGGCAATTCTACATCCAGAAGCCATTTCCCACGTACGTGTTGTACCTTGTCATCGTCATAGGCTTGACCGAATTGATTGGTATGGTGGAGATCAAGTGGGCAGGCCTCACGCTGCTGGACTGGATCCGCAATGAGCAGTTCTACATTGTTGGTGCGACTGCTGTGTACCCTACAGCAGTGCTGCACATAGTGCTGAAGTTGTTTGGTTTGAAGGGTGTTTCATTCAAGCTGACGGCAAAGCAGGTAGCAAGCAGCACCAGTGAGAAGTTTGCTGAACTGTACGCTGTGCAGTGGGCTCCAATGCTGATCCCAACAATGGTAGTGATAGCAGTGAATGTCTGTGCCATTGGTGCGTCGATAGGCAAGGCGATAATCGGAGGATGGTCGCTCTTGCAGATGGCAGATGCAGGGCTGGGGCTGCTGTTCAACGCATGGATTCTGCTGCTGATCTACCCTTTTGCACTGGGCATCATGGGACGGTGGAGCAAGAGACCCTATGTCCTGTTCATTATGTTTGTCCTTGCGTTTATTGTAATCGCGATGCTGGATATTGCCATCCAAGCCATGCGCTCTGGGTTTGTTCGGTTCCACTTCAGAAGGTCAGGTGGTGCCAGTTTTCCCACAAGCTGGGGTTTG

>Bradi1g25117

ATGGCTGCGGTAGTCACTCGCCGGGCCAATGCCCGTGTGGAGGCCCTGGACGCCGACGCCGAGAGCGGAGTGCACGGCCGCACAACGGCTGACTCGCCGGTGGCCAAGCGGGTCATCGACGCCAAGGACGACGTGTGGGTCGCCGCCGACGAGGGAGGAGACATGTACTCCGGGACCGACGCCAGCCGGCCGATCTTGTTCCGGACCATGAAGGTCAAGGGCAGCATCCTTCATCCTTACAGGTTCTTCATTCTCGTGCGGTTGGTGGCCATCGTGGCCTTCTTCGCGTGGCGGATCGAGCACCGGAACCGGGACGGCGTGTGGCTCTGGGCCACGTCCATGGTGGCCGACGTCTGGTTCGGCTTCTCCTGGCTCCTCAACCAGCTCCCAAAACTCAACCCTGTCAAACGCGTCCCGGACCTCGCCGCACTCGCCGACTCCTCCTCCGGCTCCGACGACAACCTCCCGGGCATCGACATCTTCGTCACCACCGTCGACCCCGTGGACGAGCCCATCCTCTACACCGTCAACACCATCCTCTCCATCCTGGCCACCGACTACCCCGTCGACAAGTACGCCTGCTACCTCTCCGACGACGGCGCCACGCTGGTGCACTACGAGGCCATGCTCGAGGTCGCCAACTTCGCCGTTCTCTGGGTCCCGTTCTGCCGGAAGCACTGCGTCGAGCCCAGGGCACCCGAGAGCTATTTCGGGATGAAGACGCAGCCGTATATTGGGGGTATGGCTGGGGAATTCATGAAGGATCATAGGAGAGTGAGAAGGGAATATGATGAGTTCAAGGTGAGGATTGACTCCCTTTCTAGCACCATTCGACAACGATCCGATGCGTACAATAACTCGGGCAACAAAGGACCTGGTCTTGTACGTGCGACTTGGATGGCTGACGGGACGCCGTGGCCCGGCACATGGATTGAGCAAGCTGAGAACCACCGAAAGGGACAGCACGCTGGGATTGTTCAGGTCATACTAAACCATCCAAGCCGTAAACCACAATTGGGATCACCAGCCAGCAAAGACAGTCCAATCGACTTCAGCAACGTCGACACGAGGATCCCCATGCTCGTCTACATGTCCCGGGAGAAGCGCCCCGGCTACAACCACCAAAAGAAGGCCGGCGCCATGAACGTGATGCTCCGCGTCTCCGCGTTGCTCTCCAACGCGCCATTCGTCGTCAACTTCGACTGCGACCACTACATCAACAACAACCAGGCTCTCCGTGCCCCGATGTGCTTCATGCTCGACCCGCGCGATGGACAGAACACGGCCTTCGTCCAGTTCCCGCAGCGCTTCGACGACGTGGACCCAACGGACCGCTACGCCAACCACAACCGCGTCTTCTTCGACGGCACCATGCTCTCCCTCAACGGCCTCCAGGGCCCCTCCTACCTCGGCACGGGCACCATGTTCCGCCGTGTCGCGCTCTACGGTATGGAGCCGCCACGCTGGAGAGCCGACAGCATCAAGCTCGCAGGCAAGTCACACGACTTCGGTACCTCGACGTCGCTGATAAACTCGATGCCGGATGGCGCCATCCAAGAGCGGTCTATCACACCGGTGGTTGTCGACGAGCCACTCGCCAACGAGCTGGCCGTCCTGATGACGTGTGCTTACGAGGACGGGACCTCGTGGGGCCGAGATGTCGGATGGGTGTACAACATCGCGACGGAGGACGTGGTGACCGGGTTCCGCATGCACAGGCAAGGGTGGCGCTCCATGTACTGCTCCATGGAGCCCGCCGCGTTCCGTGGCACAGCCCCAATCAACCTCACGGAGCGCCTCTTACAGGTGCTCCGCTGGTCGGGGGGATCCCTGGAGATGTTCTTCTCCCACAGCAACGCGCTCCTCGCGGGCCGCCGGCTCCACCCTCTGCAGCGGGTGGCCTACCTCAACATGTCCACCTACCCGATCGTGACGGTGTTCATCTTTGCGTACAACCTCTTCCCGGTCATGTGGCTCGTCTCGGAGCAATTCTACATCCAGAGGCCCTTCGGAACCTACATCGTGTACCTCGCCGCGGTGATCTCCATCATCCACGTCATCGGCATGTTCGAGGTGAAGTGGGCCGGGATCACGTTGTTGGACTGGTGCCGCAACGAGCAGTTCTACATGATCGGGGCCACGGGCGTGTACCCGACGGCGGTGCTGTACATGGCGATGAAGCTGGTGACCGGGAAGGGGATATACTTCCGGCTCACGTCCAAGCAGTCAGACGCATGCTCCGACGACAAGTTCGCGGACCTTTACACTGTCAGGTGGGTCCCATTGCTGATCCCGACCATCGTCGTGCTTGTGGTGAACGTCGCGGCGGTCGGGACGGCGGTGGGCAAGGCGGTGGCGTGGGGGGTTTTCACGGACCAGGCGCAGCACGCGATGCTCGGGATGGTGTTCAACGTGTGGATCCTCGTGCTCCTCTACCCGTTCGCGCTTGGGATCATGGGGCGGTGGGGCAAGAGGCCCGCCCTGCTGTTCGTCATGCTGGTCATGGCCATTGGCGCCGTGGCGCTCCTGTATATCATGCTCCATGGAGCACGGTACCCATCAGAATTGTCAGAAGTTGCGGCTTCTCTTGGTAAAGCATCGCTGACCGGGCCATCTGGG

>Bradi1g25130

ATGGCGTCGGCCACCAGTAATGGTGGGACGACGGGCAATGCCGGGCTGGCTGACCCGCTGCTGGTGAGCCGCGATCATGGCAGCACCAAGAAGCAGGTCGGTCCCAAGGGCAAGTACTGGGAGGCTGCTGACAAGGTGGAGCGCCGGGCGGCCAAGGAGAGCGGCGGCGAGGATGGCCGGCAGCTGCTGTTCCGGACGTACAAGGTCAAGGGCACCCTCTTGCACCCGTACAGGATGCTGATCTTCATACGCTTAATTGCCGTCCTTCTATTCTTTGTATGGCGCATCAGGCACAACAAATCTGACATCATGTGGTTTTGGACAATGTCTGTCGTTGGGGATGTCTGGTTCGGCTTCTCATGGCTGCTCAACCAGCTCCCAAAGTTCAACCCCATTAAAACCATACCTGATCTTGTCGCCCTAAGGCAACAATACGATCTCCCAGATGGGACATCTAGACTCCCTGGCATCGATGTCTTTGTCACCACTGCTGACCCAATTGATGAGCCAATACTATACACCATGAATTGTGTCCTCTCTATCCTAGCTTCTGACTACCCAATTGATAGGTGTGCCTGCTATCTCTCAGATGATAGTGGAGCATTAATCCTATACGAGGCATTGGTTGAGACCGCAAAATTTGCTACTTTGTGGGCTCCCTTTTGCCGGAAGCATTGTATCGAGCCAAGAGCCCCAGAAAGTTACTTTGAACAAGAGGCACCGTTGTACAGTGGAAGAGAACCAGAGGAGTTCAAGAATGATCATAGGATTGTACATAGGGAGTATGATGAGTTCAAGGAGCGCTTAGACTCACTATCTAGTGCTATTGCCAAGCGCTCCGATGTTTACAACAGCATGAAGACCGAAGAAAAAGATGTAAAGGCGACTTGGATGGCAAATGGGACACAGTGGCCAGGTGCTTGGATTGACACAACAGAAAATCATAGGAAAGGAAACCATGCCGGGATTGTTAAGGTTGTTTTGGATCATCCAATCCGTGGGCATAATCTTGGCTCACAAGCAAGCATTCACAATGACCTCAACTTCACCAACATTGATGTGCGCATCCCAATGCTCGTGTATGTCTCTCGTGGAAAGAACCCAAGCTACGACCACAATAAAAAGGCGGGTGCCTTGAATGCGCAATTGCGTGTATCTGCGCTACTCTCCAATGCACAATTCATCATCAACTTTGACTGCGACCACTACATCAATAATTCTCAAGCCCTGCGTGCAGCTGTCTGCTTTATGCTAGATCAAAGGGAAGGTGATAATACTGCCTTCGTTCAGTTCCCTCAACGTTTTGATAATGTTGATCCATCAGATCGATACGGCAACCACAATCGTGTCTTCTTTGATGGTACAATGCTCGCCCTCAATGGCCTCCAAGGGCCCTCTTACCTCGGCACTGGTTGCATGTTCCGCCGCATAGCGCTCTACGGCATTGACCCTCCTCAATGGAGACAGGCCAACATCGCAATTGAAGGTACCAGGTTTGGTAGCTCCATACCCTTCCTAGATTCCGTGTCAAAAGCCATAAACCAAGAACGGTCTACCATACCACCACCACTCAGTGACCAGTTTGTCGCTGAGATGGAGAAGGTCGCGTCAGCTTCACACGATAAGCAAACCGGGTGGGGCAAGGGTGTTGGGTACATATATGACATAGCCACAGAAGATATAGTGACCGGATTCCGCATCCACGGGCAAGGTTGGCGTTCCATGTATTGTACAATGGAGCGTGACGCCTTCTGTGGTATTGCACCCATCAACCTAACCGAGCGCCTCCACCAAATTGTGCGTTGGTCTGGTGGATCTTTAGAGATGTTCTTCTCCCGCAATAACCCACTCATTGGCGGCCATAGGATTCACACCCTTCAACGAGTCTCATACCTCAACATGACAGTCTACCCAGTCACATCACTATTTATCCTACTCTATGCTCTTAGCCCTGTGATGTGGCTTATTCCTGACGAACTATACATTCAAAGGCCATTCACCAGATATGTTGTTTACCTTCTCGTGATCATTTTGATGATCCATATAATTGGATGGCTCGAGATAAAATGGGCGGGGGTCACATGGTTGGATTATTGGAGGAATGAACAATTCTTCATGATTGGCTCGACAAGTGCATATCCCACAGCCGTGCTGCACATGGTGGTCAATCTCCTTACAAAGAAGGGTATACACTTCAGAGTGACTTCAAAGCAAACAGCTGCTGACACCAATGACAAGTTTGCTGACTTGTATGACATGCGATGGGTGCCAATGTTAATCCCGACATTGGTAGTTTTGGTTGCTAATGTTGGTGCCATTGGTGTCGCCATGGGTAAAACAATAGTGTATATGGGAGTATGGACAACAGCACAGAAGACACATGCTGCAATGGGTCTGCTATTCAATGTGTGGATTATGGTGCTCCTTTATCCGTTTGCATTGGCGATCATGGGACGGTGGGCAAAGAGACCGGTCATCCTAGTTGTCTTGTTACCGGTTGCCTTTGTAATAGTTGGCCTTGTATATGTTGCTGTTCATATCTTATTTGCTAGTTTTGTTCCAATC

>Bradi1g25150

ATGTCGTCGCCGGGGACAGAAGAAGCCGCCGGCCTCAACGAGCCGCTCCTCGCGAACGGCAATAATGGCGTCGACGAGGCAGAGGTGGCCAAGCGTAAGGGCGCCCTGAAGAGCAAGGATGACAACTGGTGTTGGGAAGACGTCGGCCAGCCGGACGACGTGGCGGCGCCGCCGGACCTGGAGAATGGCGACGGACGACGGCCGCTGCTGTTCAGGAACAGGAAAGTCAAGAACATCGTCCTTTACCCGTTCAGGGCATTGATTCTAATACGAATAATTACCCTCATCTTATTTGTTGGATGGCGGATCAAGAACAGTAATTCCGATGTCATCTGGTTTTGGGTGATGTCCATTATCGCAGATGTGTGGTTTGGTTTATCATGGCTAAGCTACCAGCTCCCAAAGTGTAACCCCATCAAAAGTATACCTGACCTTGTTACACTTAGGAAACATTGTGACCTACCAGGTGGGAGCTTCCAGCTCCCAGGCATTGACGTCATTGTTACCACTGCCAGTCCTATTGCTGAACCTATATTGTACACCATGAATTGTGTCCTGTCTATCCTTGCAGTCGACTATCATGTTGGTAAATTCACCTGCTACCTCTCTGATGATAGTGGGTCATTGATCCTTTATGAAGCACTAGTTGAGACTGCAAAGTTTGCTACTTTGTGGGTTCCCTTCTGTAGAAAGCATCGCATTGAGCCAAGAGCACCGGAAAGCTATTTTGAACTACATGGGTCGTTGTATGAAGGAGAATCACTAGAGGTGTTTATGAGTGATTATAAGCATGTGCGCACAAAATATGAGGAGTTCAAGATGTATTTGGATATGCTCTCTGATGCTATCCGCGAAAGATCAAATATTTACAATAGAATGGAAACAAAAAAAGTAGACACAAAAGCGACTTGGATGGACAATGGGACACAATGGCCAGGCACTTGGTTTGACCCAACAGAAAACCACAGGATGGGGCATCATGCAGGAATTGTTCAGATTGTACAGAGCCATCCAAACCATATGGCTCAACCAGGTCCACAAGAGGCCAACAACTATCCTCTCAACTTTGAAGATGTTGATCTGCGCCTTCCAATGCTTGTCTATGTGGCTCGTGAAAAAGGGTCAGGTTGTGAGCACAACAAAAAGGCGGGTGCTCTAAATGCCGAGCTACGCATCTCAGCTCTACTCTCTAATGCACCGTTCTTCATCAACTTTGATTGTGATCATTACATCAACAACTCACAAGCCCTGCTTGCAGCTATTTGTTTCATGCTGGACCGTCGAGAAGGGGATAATACTGGATTTGTCCAATTCCCACAAAGGTTTGATAATGTTGACCCTACAGATCGATATGGAAACCACAACCGGGTATTTTTTGATGGAGCCATGTATGGTCTCAACGGTCAACAAGGGCCTACTTACCTTGGTACTGGTTGCATGTTCCGCCGCCTTGCGCTCTATGGCATTGATCCACCTTGTTGGAGATCCAAGGAGATCATAATCAACAGTAATAAGTTTGGTAACTCACTACCCTTTCTAAATTCAGTACTAGCAGCCATAAAACAAGAACAATGTGTCACACCACCACTAGATGATTCTTTTGTTGCTGAGATGACGAGAGTTGTGTCATCTTCGTATGATGATTCAACGGACTGGGGCAGGGGTGTTGGCTACATTTATAAAATGGCAACCGAGGACATAGTAACCGGCTTTCGCATCCACGGGCAAGGGTGGCGCTCCATGTATTGTAGTATGGAGCGTGAAGCGTTCCGTGGCACGGCACCGATCAACCTAACCGAGCGCCTCCACCAAATAGTGCGATGGTCTGGCGGCTCCCTGGAGATGTTCTTCTCCTACATGAGTCCATTATTTGCTGGTCATCGACTCAACACCATGCAACGGGTCTCATACATTAATTTTACTATCTACCCAATCACATCGCTCTTTATCCTAATGTATGCTCTTTGTCCAGTGATGTGGCTTCTCCCAACAGAAATATTTATACAGAGGCCATATACTAGGTACATTGTTTACCTTTTTATCGTAATTGGGATGATCCATGTGATTGGCATGTTCGAGATAATGTGGGCAGGGATCACATGGTTGGATTGGTGGCGCAGCGAGCAGTTTTTTATAGTCAGCTCAGTAAGTGCTTATCCAACGGCGGTGTTGCACATGGTGGTCAATCTCCTTACAAAGAAAGGTATAAAATTCAGGGTCACTGAAAAGCAATCGGTGGTTGATACGGACGATAAGTATGCGGAGATGTATGAATTGCGATGGGTGCCCATGATGATCCCAGCAGTTGTGGTTTTGTTCTCCAACATCATTGCTATTGGTGTAGCAATTGGTAAATCAATCCTATACATGGGGACATGGACTCCAGCGCAGAAGAGGCATGGTGCACTAGGCCTGATGTTCAATGTGTGGATTATGGTTCTCCTTTACCCATTTGCATTGGCGATTATTGGACGATGGGCCAAGAAAACCGGCATCCTCTTCATCTTGTTACCGATTACATTTTTGTCTATTGCGATTATGTATATTGGCATCCATACCTTTCTTTCAAATTTCCTTCCATCCATGGTCATA

>Bradi1g25157

ATGGAGGCCAGCTGCATGTCTTCGCCGGTGACCACGGACGCCGCCGGCGCCGTCCGCGTGGACGATGATGCCGCCGGGCTCAGCAAGCCGCTCCTGGCGAACGGCAAGGAGAAGAAAGGTGCCGTGAAGGCCAGTGAGAGGTACTGGGTGGATGTCGACCAGCCGGACGTGGCGTCGGCGGCAGATTTGGAAGGCGGCAGCGGCCGGCCGCTGCTGTTCCGGAACAGGAGGGTCAAGAACATACTTCTCTACCCGTACAGGGCATTGACTGTCATACGTTTGATTGCCGTCATCTTCTTCATCACATGGCGCATCAAGCACAACAAATCAGACGTCATGTGGTTTTGGGTGACGAGCATCGTCGGGGATGTATGGTTTGGCTTATCATGGCTAAGCTACCAGCTCCCAAAGTTCAACCCCATCAAACGGGTACCTGACCTTGCTACACTCAGGCAACACTATGACCTACCAGATGGGAGTTCCCATCTCCCAGGCATTGACGTCATCGTCACCACTGCCAGTCCTATAAATGAACCCATTTTATACACCATGAATTGTGTCCTCTCCGTCCTTGCAGCCGACTACCATATTGATAGATACACCTGCTACTTATCAGATGATAGTGGATCATTGATCGTTTACGAGGCATTGGTTGAGACTGCAAAGTTTGCTGCTATTTGGGTTCCCTTTTGTCGAAAGCATCGCATTGAGCCGAGAGCACCAGAGAGCTATTTTGAATCAGAGGAGTCGGTGATGGTGTACAGAGGAAGACCACAGCAAGAGTTGATGAGCGATTATAAGCATGTGCGTGCGCAATATGAAGAGTTCAAAGTCTACTTGGATAAGCTTCCTAATAGCATTCAACAGAGGTCAGATGTTTACAATGGAATGGAAACAAAGGGTGGTCATGCAAAAGCGACCTGGATGGCTAATGGGACACAATGGTCGGGCACATGGATTGATCCAATAGAAAACCACAGGACCGGTCATCATGCAGGAATTGTTCAGATTGTGCAGGAGCATCCAAAACATATGGCCCAACAGAGCATTGGCAACCCTCTAAACGTTGATGATGCTGATTTACTCCTCCCGATGCTTGTCTACGTGTCTCGTGAAAAGAGCCCACATTATGACCACAATAAGAAAGCGGGTGCCCTGAATGCGCAGTTACGTATCTCCGCCTTGCTCTCCAATGCTCCATTTATTATCAACTTTGATTGCGACCACTACATCAACAATTCACAAGCACTACGCGCAGCCGTGTGCTTCATGCTAGACCAACGAGAAGGAGAGAACACAGCATTTGTTCAATTCCCGCAACGGTTTGAAAATGTTGACCCGACAGATCGATATGGGAACCACAACCGTGTCTTCTTTGATTGTGCCATGTATGGCCTTAATGGTCTGCAAGGGCCTACATATCTTGGCACTGGCTGCATGTTTCGCCGTGTTTCACTCTACGGCATTGATCCGCCTTGTTGGAGACCAGATGACATCATAGTCGACACCAGCAAGTTTGGTAATTCGGTACCCTTTTTGAAATCTGTGTTAACAGCCATCAAGCAAGAACGGTATGTCACACCACCACCATTAGATGAGCTGTTTCTTTCTGAGATGATTGCTGTTGTGTCATCTTCATATGATAAGGAGACAGAGTGGGGTAGGAGTGTTGGCTACATTTACAACATAGCAACAGAGGACATAGTAACTGGTTTCCGTATCCATGGGCAAGGGTGGCGCTCCATGTATGGTACATTATTAGAACGTGAAGCGTTCGTTGGCACTGCGCCAATCAACCTAACCGAGCGCCTACACCAAATAGTACGATGGTCCGGTGGCTCCCTAGAGATGGTCTTCTCCCATAACAATCCATTCTTTGCTGGGCCTCGACTCCAATGGCTACAACGGGTTTCATATATCAATTTCACTGTCTATCCAATCACATCACTCTTCATACTAATGTATGCCTTATGTCCAGTGATGTGGCTTCTACCACGGGAAATATTCATTCAAAAGCCGTTCGCTACCTATGTTTTATACCTTATCGCTATCATAGTGATGATCCAGACAATTGGCTTGTTTGAGATAAAGTGGGCAGGGATCAGATGGCTCGATTGGTGGCGCAACGAGCAGCTCTTCATGATCGGTTCCACTAGTGCTTACCCGGTGGCGGTGATGCACATGGTAGTGAAACTTCTCTTGAGGAAGGGTATATATTTTAGAGTCACTACAAAGCAAGCAGTGGTTGATATGGACGACAAGTTTGCTGAGTTGTATGAATTGCGATGGGTGCCCATGATGATCCCTGCGATAGTGGTGTTGTTCTCCAACATCTTGGCTATTGGTGTAGCGATAGGTAAATTCATCCTATACATAGGGACATGGTCAGCGGTGCAGCAGAGGAATGCTGCACTAGGTCTCATGTTCAATATGTGGGTTACAATGCTCCTTTACCCATTTGCACAAGCAGTGATTGGACGGTGGGGCAAGAGACCCGGCATCCTCTACATCCTGTTACCGATTGCTTATGTAGCCATTGCGCTGATGTATCTTTGCATCCATGCATTCCTTGTCCATTTCCTTCCATCCATGGTGATA

>Bradi3g16307

ATGGCGCCAGCGGTGGCCGGCGGGAGCAGCCGGGGTGCAGGGTGTAAGTGCGGGTTCCAGGTGTGCGTGTGCTCTGGGTCGGCGGCGGTGGCGTCGGCGGGTTCGTCGCTGGAGGTGGAGAGAGCCATGGCGGTGACGCCGGTGGAAGGGCAGGCGGCGCCGGTGGACGGCGAGAGCTGGGTCGGCGTCGAGCTCGGCCCCGACGGCGTGGAGACGGACGAGAGCGGCGCCGGCGTCGACGACCGCCCCGTCTTCAAGACCGAGAAGATCAAGGGCGTCCTCCTCCACCCCTACAGGGTGCTGATCTTTGTTCGTCTGATAGCGTTCACCCTGTTCGTGATCTGGCGTATCTCGCACAAGAACCCGGACACGATGTGGCTGTGGGTGACCTCCATCTGCGGCGAGTTCTGGTTCGGCTTCTCCTGGCTGCTGGACCAGCTTCCAAAGCTCAACCCGATCAACCGGATCCCGGACCTCGCCGTGCTCCGGCAACGCTTCGACCGCGCCGACGGGACATCCACATTGCCGGGCCTCGACATCTTCGTCACCACGGCCGACCCCATCAAGGAACCCATCCTGTCGACGGCCAACTCCGTGCTCTCCATCCTGGCCGCCGACTACCCGGTGGACCGCAACACCTGCTACATCTCCGACGACAGCGGCATGCTCATGACCTACGAGGCCATGGCGGAGTCGGCCAAGTTCGCCACCCTCTGGGTGCCATTCTGCCGCAAGCACGGCATCGAACCACGCGGGCCGGAGAGCTACTTCGAGCTCAAGTCGCACCCGTACATGGGGAGAGCGCACGACGAGTTCGTCAATGACCGCCGCCGGGTGCGCAAGGAGTATGATGACTTCAAGGCCAAGATTAACTCTCTGGAGACTGATATCCAGCAGAGGAATGATCTGCATAACGCTGCCGTGCCGCAGAATGGGGATGGGATCCCCAGGCCTACCTGGATGGCTGATGGAGTCCAGTGGCAGGGGACTTGGGTCGAGCCGTCCGCTAATCACCGCAAGGGAGACCACGCCGGCATCGTCCTGGTTCTGATTGACCACCCGAGCCACGACCGCCTTCCCGGCGCGCCGGCGAGCGCCGACAACGCGCTGGACTTCAGCGGCGTGGACACCCGCCTCCCGATGCTCGTCTACATGTCCCGCGAGAAGCGCCCAGGCCACAACCACCAGAAGAAGGCCGGCGCCATGAACGCGCTCACCAGGGCTTCCGCGCTGCTCTCCAACGCGCCCTTCATCCTCAACCTCGACTGCGACCACTACATCAACAACTCCCAGGCCCTCCGCGCCGGGATCTGCTTCATGGTCGGCCGGGACAGCGACACCGTCGCCTTCGTGCAGTTCCCGCAGCGGTTCGAGGGCGTCGACCCCACGGACCTCTACGCCAACCACAACCGCATCTTCTTCGACGGCACCCTCAGGGCGCTCGACGGAATGCAAGGCCCGATCTATGTCGGCACGGGATGCCTCTTCCGGCGCATCACCGTCTACGGCTTCGACCCGCCCAGGATCAACGTCGGCGGGCCATGCTTCCCTGCTCTCGGTGGCCTGTTCGCCAAGACCAAGTATGAGAAGCCCAGCATGGAGATGACCATGGCGAGAGCCAACCAGGCCGTGGTGCCGGCCATGGCCAAGGGGAAGCACGGCTTCCTGCCGCTCCCCAAGAAGACGTACGGGAAGTCCGACAAGTTCGTGGACACCATCCCGCGCGCGTCCCACCCGTCGCCGTACGCGGCGGAGGGGATCCGCGTGGTGGACTCCGGCGCGGAGACTCTGGCTGAGGCCGTCAAGGTGACCGGATCGGCATTCGAGCAGAAGACCGGATGGGGCAGCGAGCTCGGCTGGGTCTACGACACTGTCACAGAGGACGTGGTGACTGGCTACAGGATGCACATCAAGGGCTGGAGGTCCCGCTACTGCTCCATCTACCCGCACGCCTTCATCGGCACCGCCCCGATCAACCTCACGGAGCGGCTCTTCCAGGTGCTCCGCTGGTCCACCGGCTCCCTCGAGATCTTCTTCTCCAAGAACAACCCGCTCTTCGGCAGCACCTACCTGCACCCGCTCCAGCGCGTCGCCTACATCAACATCACCACATACCCGTTCACCGCCATCTTCCTCATCTTCTACACCACCGTGCCGGCGCTCTCCTTCGTCACCGGCCACTTCATCGTGCAGCGCCCGACGACCATGTTCTACGTCTACCTGGGGATCGTGCTGGCGACGCTGCTCATCATCGCTGTTCTTGAGGTCAAGTGGGCTGGAGTGACAGTGTTCGAGTGGTTCAGGAACGGGCAGTTCTGGATGACGGCTAGCTGCTCCGCCTACCTTGCTGCTGTGTGCCAGGTGCTCACCAAGGTGATCTTCAGGAGGGACATCTCATTCAAGCTCACTTCCAAGCTGCCTGCTGGGGACGAGAAGAAGGACCCCTATGCCGATCTGTACGTGGTGCGTTGGACTCCACTCATGATCACTCCAATCATCATCATCTTCGTCAACATCATCGGCTCGGCGGTGGCCTTCGCCAAGGTGCTGGACGGCGAGTGGACGCACTGGCTCAAGGTGGCGGGAGGAGTCTTCTTCAACTTCTGGGTGCTGTTCCACCTCTACCCGTTCGCCAAGGGTCTCCTGGGGAAGCATGGCAAGACCCCCGTCGTCGTGCTCGTCTGGTGGGCATTCACCTTCGTCATCACCGCCGTCCTCTACATCAACATCCCGCACATCCATGGAGGAGGAGGCAAGCACAGCGTGGGGCATGGGATGCACCATGGCAAGAAGTTCGACGGCTACTACCTCTGGCCG

>Bradi3g45515

ATGGCTTCGGCGCCCGCGGCCGGCGCTGGTGGCAATGCGGGTGGTTTAGCCGAGCCGCTGCTGGCGGCGGCGAACGGCGGCGGCGTTGGCGCCAAGGACAAGTACTGGGTTCCCGCCGACGAGAAAGAGGAGATGCTGGGGGCGTCGCAGGAGGACGGCCGGCCGCCGCAGCAGCCGTTGCTGTACCGCACGTTCAGGGTCAAGGGCGCCCTCATCAACCTCTACAGGTTGTTGACCCTGGTTAGAGTTATTGTGGTTATCCTATTCTTCACTTGGCGCATGAAGCACCGGGACTCCGATGCAATGTGGTTGTGGTGGATCTCGGTCGTTGGGGACCTGTGGTTCGGCGTAAGCTGGCTGCTAAACCAACTGACCAAGCTCAAGCCCAGGAAATGCATCCCCAACCTTTCCCTCCTGAGAGAACAATTTGAACAGCAGCCTGTCGATGGCTCCAGCTCTGGCCTCCCTGTCCTTGACGTGTTCATTAACACTGTCGACCCAGTGGATGAACCCATGCTCTATACCATGAACTCTGTCTTGTCCATCCTCGCCACCGATTACCCAGCTGAGAAGCACGCCACCTACTTCTCTGATGATGGCGGGTCACTGGTGCACTATGAGGGGTTACTTGAAACTGCAAAGTTTGCTGCATTGTGGGTTCCATTTTGCCGGAAGCATTGCGTCGAGCCAAGAGCACCAGAGAGCTACTTCTGGACGAAGACGCGGCTCTATGCCGGGAACGCCCCCGAGGAGTTCGTTGATGACCATAGGTGCATGCACGTGGAGTATGAAGAGTTTAAGGCACGATTGGATGCGCTTTCTACTGTCATTGCGCAACGATCAGAGGCCTGCAACCATGCAAACACCAAAGTAAGGTGTGAAAATGCAACTTGGATGCTGGATGGAACACAATGGCAAGGCACCTGGGTTGAGCCAGCGACCGGGCATAGGAAAGGACATCACCCTGCAATTCTTCAGGTCATGTTGAATCAACCAAGCAATGAGCCTCAGCTCGGCATGCCAGCAAGCTCTGACAACCCGCTAGACTTCAGCACGGTCGATGTGCGCCTCCCAATGCTGGTGTACATCTCCCGCGAGAAGCGGCCGGGCTACGACCACCAGAAGAAGGCGGGCGCCATGAACGTGCAGCTGCGTGTGTCGGCGCTGCTGTCGAACGCGCCCTTCATCATCAACTTCGACGGCGACCACTACATCAACAACTCGCAGGCCTTCCGCGCAGCCATGTGCTTCATGCTGGACCGGCGTGATGGCGACGACACTGCCTTTGTGCAGTTCCCACAGCGCTTCGACGATGTCGACCCAACGGACCGGTACTGCAACCACAACCGCATGTTCTTCGATGCCACCCTGCTGGGCCTCAATGGAATCCAGGGCCCCTCCTTCGTTGGCACCGGCTGCATGTTTCGCCGCGTCGCGCTCTATGGTGCTGATCCGCCTCGGTGGCAGCCTGACGACGACTCCAAGGCCTTGCAACAACACAGTCCAAACATTTTCGGCACATCGGCGGCGTTCGTCAACTCGCTGCCGATGGCCGCTGACCAAGAACGTTCCGTCGCGACACCGGTGACGCTCGATGAGGCGGAGCTGAGCGACGTGATGACGTGCGCTTACGAGGACAGCACGGAGTGGGGCAACGGCGTCGGCTGGGTGTACAACATTGCGACAGAGGATGTGGTGACTGGCTTCCGGCTGCACCGGGCAGGCTGGCGGTCTATGTACTGCGCCATGGAGCCGGATGCGTTCCGCGGCACGGCACCGATCAACCTGACGGAGCGGCTATACCAGATCCTGCGCTGGTCGGGGGGGTCCCTGGAGATGTTCTTCTCCCGCTTCTGCCCGCTCCTCGCCGGCCGCCGGCTCCACCCCATGCAGCGCATCGCCTACGTCAACATGACAACCTACCCAGTCTCGACCTTCTTCATCGTCATGTATGACCTGTACCCGGTGATGTGGCTCTTCCACGGCCATTTCTACATCCAGAAGCCGTTCCAGACGTTCGCTCTGTTCGTTGCTGTCATCATCGCCACGGTGGAGGTGATCGGCATGGTCGAGGTGAAGTGGGCGGGGCTCACGCTGCTCGACTGGTTCCGGAACGAGCAGTTCTACATCATCGGCACGACAGGGGTGTACCCGACAGCGATGCTGCATATCCTGCTCAGGTCCCTCGGCCTCAAAGGGGTGTCCTTCAAGTTGACAGCCAAGAAGCTGATGACGGCCGGCAGCGCTAGGGAGAGGCTTGCAGAGCTGTACGACGTGCAATGGGCGCCTCTGCTGGCACCCACGGTGGTGGTGTTGGCTGTGAATGTGGCCGCAATTGGCGCGGCGGTGGGAAAGGCAGTTGCCTGGCGATGGTCGACCGTGCAGGTTGCCGAGGCAGCGACCGGGCTGACGTTCAATGTGTGGATGCTGCTGCTGCTCTACCCCTTCGCGCTTGGGATCATGGGGCTCTGGAGCAAGAGGCCGTATGTCCTCTTCGCCTTGCTCTTGGCCGCGGTCGCTGCCACCGCCTCGGTTTATGTCGTTCTGGTTGGCTCCGTGCCGGATTTCCTCTCTTCTCGGGATCTTGGCTCCATTCCATCTAGTAAGCTAGTT

>OsCSLF1

ATGTCCGCGGCGGCAGCGGTGACAAGCTGGACTAACGGATGCTGGTCGCCCGCGGCTACGCGGGTGAACGACGGCGGCAAGGACGATGTGTGGGTGGCCGTCGACGAAGCGGACGTGTCGGGGGCCCGCGGCAGCGACGGCGGCGGCCGGCCGCCGCTGTTCCAGACGTACAAGGTCAAGGGCAGCATCCTTCATCCTTACAGGTTCTTGATCCTGGCGCGACTGATCGCCATCGTCGCCTTCTTCGCGTGGCGCATACGTCACAAGAACCGCGACGGCGCGTGGCTGTGGACAATGTCCATGGTCGGCGACGTCTGGTTCGGCTTCTCGTGGGTGCTCAACCAGCTACCGAAGCAGAGCCCCATCAAGCGCGTCCCGGACATCGCCGCCCTCGCCGACCGGCACTCCGGCGACCTACCCGGCGTCGACGTCTTCGTCACCACCGTCGACCCTGTCGACGAGCCGATACTCTACACCGTGAACACCATCCTCTCCATTCTCGCCGCCGACTACCCGGTGGACAGGTACGCTTGCTACCTCTCCGACGACGGCGGGACGCTGGTCCACTACGAGGCTATGGTGGAGGTCGCCAAGTTCGCCGAGCTGTGGGTGCCCTTCTGCCGGAAGCACTGCGTCGAGCCGAGGTCGCCGGAGAACTACTTCGCGATGAAGACGCAGGCGTACAAAGGCGGCGTCCCCGGCGAGCTGATGAGCGATCACCGGCGTGTGCGGCGAGAGTACGAGGAGTTCAAGGTCAGGATCGACTCCCTCTCGAGCACCATTCGCCAGCGATCTGATGTGTACAACGCCAAACATGCCGGCGAAAATGCGACATGGATGGCTGATGGCACACATTGGCCCGGCACATGGTTTGAGCCGGCTGACAACCACCAGAGAGGGAAACATGCTGGAATTGTTCAGGTTTTACTGAACCATCCAAGCTGTAAACCGAGGCTTGGATTGGCGGCGAGTGCTGAGAATCCGGTTGATTTCAGCGGTGTCGACGTGCGGCTCCCCATGCTGGTGTACATCTCGCGCGAGAAGCGGCCCGGATACAACCACCAGAAGAAGGCCGGCGCCATGAACGTGATGCTCCGCGTGTCCGCGCTGCTGTCGAACGCGCCGTTCGTCATCAACTTCGACGGCGACCACTACGTCAACAACTCGCAGGCGTTCAGGGCGCCGATGTGCTTCATGCTCGACGGCCGCGGCCGCGGCGGCGAGAACACGGCGTTCGTCCAGTTCCCGCAGCGGTTCGACGACGTTGACCCGACGGACCGGTACGCGAACCATAACCGCGTCTTCTTCGACGGCACCATGCTCTCCCTCAACGGCCTCCAGGGGCCCTCCTACCTCGGCACCGGCACCATGTTCCGCCGCGTCGCGCTCTACGGCGTGGAGCCGCCGCGCTGGGGAGCGGCGGCGAGCCAGATCAAGGCTATGGACATCGCCAACAAGTTCGGCAGCTCGACGTCGTTCGTCGGCACGATGCTGGACGGCGCCAACCAAGAACGGTCGATCACGCCGCTGGCGGTGCTCGACGAGTCGGTCGCCGGCGACCTCGCCGCCCTGACGGCGTGCGCGTATGAGGACGGGACGTCATGGGGGAGAGACGTCGGGTGGGTGTACAACATCGCGACGGAGGACGTGGTGACCGGGTTCCGCATGCACCGGCAGGGGTGGCGCTCCGTATACGCCTCAGTGGAGCCCGCCGCGTTCCGCGGCACGGCGCCGATCAACCTCACCGAGCGCCTCTACCAGATCCTCCGGTGGTCGGGCGGCTCGCTGGAGATGTTCTTCTCCCACAGCAACGCGCTCCTCGCCGGCCGCCGCCTCCACCCGCTGCAGCGCGTCGCCTACCTCAATATGTCCACCTACCCGATCGTGACCGTGTTCATCTTCTTCTACAACCTCTTCCCGGTGATGTGGCTCATCTCCGAGCAGTATTACATCCAGCGGCCGTTCGGCGAGTACCTCCTCTACCTCGTCGCCGTCATCGCCATGATCCACGTGATCGGCATGTTCGAGGTGAAGTGGGCTGGCATCACGCTGCTGGACTGGTGCCGCAACGAGCAGTTCTACATGATCGGATCCACGGGGGTGTACCCGACGGCGGTGCTGTACATGGCGCTCAAGCTCGTCACCGGGAAGGGCATCTACTTCCGCCTCACGTCGAAGCAGACGGCAGCCAGCTCCGGCGACAAGTTCGCCGACCTGTACACCGTGCGGTGGGTGCCTCTGCTGATCCCGACCATCGTCATCATGGTCGTGAACGTCGCCGCCGTCGGGGTGGCGGTCGGCAAGGCGGCGGCGTGGGGGCCGCTCACCGAGCCGGGGTGGCTCGCCGTGCTCGGGATGGTGTTCAACGTGTGGATCCTGGTGCTCCTCTACCCGTTCGCGCTCGGGGTCATGGGTCAATGGGGGAAGCGGCCGGCCGTGCTGTTCGTGGCGATGGCGATGGCCGTCGCCGCCGTGGCGGCCATGTACGTCGCCTTCGGTGCACCGTACCAAGCTGAGTTGTCAGGTGTTGCTGCTTCTCTCGGTAAAGTGGCGGCGGCATCGCTGACTGGGCCATCTGGG

>OsCSLF2

ATGGCGGCCACCGCGGCTTCCACGATGTCCGCAGCGGCGGCAGTGACTCGCCGGATCAACGCTGCCCTCCGCGTGGACGCCACCAGCGGTGACGTCGCGGCCGGCGCCGACGGGCAGAACGGGCGCCGGTCGCCCGTGGCCAAGCGGGTGAACGACGGCGGTGGCGGCAAGGATGACGTGTGGGTGGCCGTCGACGAAAAGGACGTGTGCGGGGCCCGCGGCGGCGATGGCGCCGCCCGGCCGCCGCTGTTCCGGACGTACAAGGTCAAGGGCAGCATCCTTCATCCTTACAGGTTCCTGATCCTTCTTCGACTGATCGCCATCGTCGCCTTCTTCGCGTGGCGCGTACGTCACAAGAACCGCGACGGCGTGTGGCTGTGGACAATGTCCATGGTCGGCGACGTCTGGTTCGGCTTCTCGTGGGTGCTCAACCAGCTCCCGAAGCTGAGCCCCATCAAGCGCGTCCCGGACCTCGCCGCCCTCGCCGACCGGCACTCCGGCGACCTACCCGGCGTCGACGTCTTCGTCACCACCGTCGACCCCGTCGACGAGCCGATACTCTACACCGTGAACACCATCCTCTCCATCCTCGCCGCCGACTACCCGGTGGACAGGTACGCCTGCTACCTGTCCGACGACGGCGGGACGCTGGTCCACTACGAGGCCATGGTGGAGGTCGCCAAGTTCGCCGAGCTGTGGGTGCCCTTCTGCCGGAAGCACTGCGTCGAGCCGAGGTCGCCGGAGAACTACTTCGCGATGAAGACGCAGGCGTACAAAGGCGGCGTCCCCGGCGAGCTGATGAGCGATCACCGGCGTGTGCGGCGAGAGTACGAGGAGTTCAAGGTCAGGATCGACTCCCTCTCGAGCACCATTCGCCAGCGATCAGATGTGTACAACGCCAAACATGCCGGCGAAAATGCGACATGGATGGCTGATGGCACACATTGGCCCGGCACATGGTTTGAGCCGGCTGACAACCACCAGAGAGGGAAACATGCTGGAATTGTTCAGGTTTTACTGAACCATCCAAGCTGTAAACCGAGGCTTGGATTGGCGGCGAGTGCTGAGAATCCGGTTGATTTCAGCGGTGTCGACGTGCGGCTCCCCATGCTGGTGTACATCTCGCGCGAGAAGAGGCCCGGATACAACCACCAGAAGAAGGCCGGCGCCATGAACGTGATGCTCCGCGTGTCCGCGCTGCTGTCGAACGCGCCGTTCGTCATCAACTTCGACGGCGACCACTACGTCAACAACTCGCAGGCGTTCAGGGCGCCGATGTGCTTCATGCTCGACGGCCGCGGCCGCGGCGGCGAGAACACGGCGTTCGTCCAGTTCCCGCAGCGGTTCGACGACGTTGACCCGACGGACCGGTACGCGAACCATAACCGCGTCTTCTTCGACGGCACCATGCTCTCCCTCAACGGCCTCCAGGGGCCCTCCTACCTCGGCACCGGCACCATGTTCCGCCGCGTCGCGCTCTACGGCGTGGAGCCGCCGCGCTGGGGAGCGGCGGCGAGCCAGATCAAGGCTATGGACATCGCCAACAAGTTCGGCAGCTCGACGTCGTTCGTCGGCACGATGCTGGACGGCGCCAACCAAGAACGGTCGATCACGCCGCTGGCGGTGCTCGACGAGTCGGTCGCTGGCGATCTCGCCGCCCTGACGGCGTGCGCATACGAGGATGGGACGTCGTGGGGGAGAGACGTCGGGTGGGTGTACAACATCGCGACGGAGGACGTGGTGACCGGGTTCCGCATGCACCGGCAGGGGTGGCGCTCCGTGTACGCCTCAGTGGAGCCCGCCGCGTTCCGCGGCACGGCGCCGATCAACCTCACCGAGCGCCTCTACCAGATCCTCCGGTGGTCGGGCGGCTCGCTGGAGATGTTCTTCTCCCACAGCAACGCGCTCCTCGCCGGCCGCCGCCTCCACCCGCTGCAGCGCGTCGCCTACCTCAACATGTCGACCTACCCGATCGTGACCGTGTTCATCTTCTTCTACAACCTCTTCCCGGTGATGTGGCTCATCTCCGAGCAGTACTACATCCAGCGGCCGTTCGGCGAGTACCTCCTCTACCTCGTCGCCGTCATCGCCATGATCCACGTGATCGGCATGTTCGAGGTGAAGTGGGCTGGCATCACGCTGCTCGACTGGTGCCGCAACGAGCAGTTCTACATGATCGGCTCCACGGGGGTGTACCCGACGGCGGTGCTGTACATGGCGCTCAAGCTCGTCACCGGGAAGGGCATCTACTTCCGGCTCACGTCGAAGCAGACGACGGCCAGCTCCGGCGACAAGTTCGCCGACCTGTACACCGTGCGGTGGGTGCCGCTGCTGATACCGACCATCGTCATCATTGTCGTGAACGTCGCCGCCGTCGGGGTGGCGGTCGGCAAGGCGGCGGCGTGGGGGCCGCTCACCGAGCCGGGGTGGCTCGCCGTGCTCGGGATGGTGTTCAACGTGTGGATCCTGGTGCTCCTCTACCCGTTCGCGCTCGGGGTCATGGGTCAATGGGGGAAGCGGCCGGCCGTGCTGTTCGTGGCGATGGCGATGGCCGTCGCCGCCGTGGCGGCCATGTACGTCGCCTTCGGTGCACCGTACCAAGCTGAGTTGTCAGGTGGTGCTGCTTCTCTCGGAAAAGCGGCGGCGTCGCTGACCGGGCCATCCGGG

>OsCSLF3

ATGGCTTCGCCGGCGTCGGTCGCCGGCGGTGGTGAGGATAGCAATGGCTGCAGCAGCCTCATCGACCCGCTGCTAGTGAGCCGCACGAGCAGCATCGGCGGCGCGGAGAGGAAGGCGGCCGGCGGCGGCGGCGGCGGCGCCAAGGGGAAGCACTGGGCCGCCGCCGATAAGGGGGAGCGGCGCGCGGCGAAGGAGTGCGGCGGCGAGGACGGCCGCCGGCCGCTGCTGTTCAGGTCGTACAGGGTCAAGGGCTCCCTCCTGCACCCGTACAGGGCTCTGATCTTTGCACGCTTGATTGCCGTTCTCCTCTTCTTCGGATGGCGGATCAGGCACAATAATTCTGACATAATGTGGTTCTGGACAATGTCAGTCGCCGGTGATGTTTGGTTTGGTTTTTCATGGCTACTTAACCAACTCCCAAAGTTCAATCCGGTCAAAACCATACCTGACCTTACTGCCCTAAGGCAGTACTGTGATCTCGCCGACGGAAGCTACAGACTTCCCGGCATCGATGTTTTCGTCACCACCGCTGATCCAATCGACGAACCGGTTCTATACACCATGAATTGTGTTCTCTCTATTCTTGCGGCTGACTACCCTGTTGATAGGTCAGCCTGCTATCTCTCTGATGATAGTGGAGCATTGATCCTATATGAAGCATTGGTTGAGACAGCCAAATTTGCTACTCTATGGGTTCCATTTTGCCGGAAGCATTGCATTGAGCCTAGATCCCCAGAGAGCTACTTTGAGCTTGAGGCACCATCGTATACTGGAAGTGCACCAGAGGAGTTCAAGAATGACTCTAGGATTGTGCATCTTGAGTATGATGAGTTCAAGGTGCGATTGGAAGCACTTCCTGAGACTATTCGTAAACGATCAGATGTTTACAATAGTATGAAAACTGATCAAGGAGCACCAAATGCGACTTGGATGGCTAATGGGACCCAATGGCCAGGCACGTGGATTGAGCCAATAGAAAATCACAGGAAAGGACACCATGCTGGAATTGTTAAGGTTGTGTTGGACCATCCCATCCGTGGCCACAATCTTAGCCTGAAGGATAGCACGGGCAACAATCTTAATTTTAATGCCACTGATGTGCGCATCCCGATGCTTGTCTATGTGTCTCGTGGAAAGAACCCAAATTATGATCATAATAAGAAGGCGGGTGCATTAAATGCGCAACTTCGTGCCTCTGCTCTACTCTCCAATGCACAATTCATCATCAACTTTGATTGTGATCACTACATCAACAATTCTCAAGCCTTCCGTGCAGCAATTTGTTTCATGCTTGACCAAAGAGAAGGTGATAATACTGCCTTTGTTCAGTTCCCACAACGCTTTGACAATGTTGACCCAAAAGACCGATATGGCAATCATAATCGTGTATTCTTTGATGGCACAATGCTTGCCCTAAATGGTCTCCAAGGACCTTCATACCTTGGTACTGGTTGCATGTTCCGTCGCTTAGCTCTCTATGGTATTGATCCTCCTCATTGGAGACAAGACAACATCACACCTGAAGCTAGCAAGTTTGGTAACTCCATACTCTTATTAGAGTCAGTGTTAGAAGCCCTAAACCAAGACCGATTTGCTACACCATCACCGGTCAATGACATATTTGTCAATGAGCTGGAGATGGTTGTGTCAGCTTCATTCGACAAAGAAACCGATTGGGGCAAGGGTGTTGGATACATATATGACATAGCCACAGAAGATATAGTCACGGGTTTTCGCATCCATGGGCAAGGTTGGCGATCCATGTATTGCACCATGGAGCATGATGCATTCTGTGGCACTGCACCTATAAATCTAACAGAACGTCTTCACCAAATTGTACGTTGGTCTGGTGGATCCCTAGAGATGTTCTTCTCCCACAATAACCCACTTATTGGAGGTCGTCGGCTCCAACCTCTCCAGCGTGTCTCATACCTCAATATGACAATCTACCCGGTGACATCACTCTTTATTTTACTCTATGCTATCAGCCCTGTGATGTGGCTTATCCCCGATGAAGTATATATTCAGAGGCCATTCACTAGGTATGTGGTGTACCTTCTCGTGATCATTTTGATGATTCATATGATCGGATGGCTCGAGATAAAGTGGGCAGGGATCACATGGTTAGATTATTGGCGCAATGAGCAGTTCTTCATGATCGGCTCAACGAGTGCTTACCCAACAGCTGTGCTTCATATGGTGGTCAATCTTCTCACAAAGAAGGGTATACATTTTAGAGTCACTTCAAAGCAAACAACCGCTGACACCAACGATAAATTTGCTGACTTATATGAGATGAGATGGGTTCCCATGTTAATCCCAACAATGGTAGTTTTAGTTGCTAATATCGGTGCCATTGGTGTAGCTATTGGAAAGACGGCAGTATATATGGGAGTATGGACGATAGCACAGAAGAGACATGCTGCAATGGGACTCCTATTCAACATGTGGGTTATGTTTCTCCTTTACCCATTTGCACTAGCAATCATGGGGAGATGGGCAAAGAGGTCAATCATTCTTGTTGTTTTGTTGCCAATTATCTTTGTAATTGTTGCCCTTGTATATGTTGCTACCCATATCTTACTAGCAAACATTATTCCATTC

>OsCSLF4

ATGTCCGCGGCGGCCGTGACTCGCCGGATCAACGCGGGCGGCCTCCGCGTCGAGGTCACCAACGGCAATGGCGCGGCCGGCGTCTACGTGGCGGCGGCGGCGGCACCGTGCTCGCCGGCGGCCAAGCGGGTGAACGACGGCGGCGGCAAGGATGACGTGTGGGTGGCCGTCGACGAGGCGGACGTGTCGGGGCCCAGCGGCGGCGATGGCGTTCGGCCGACGCTGTTCCGGACGTACAAGGTCAAGGGCAGCATCCTGCATCCTTACAGGTTCTTGATCCTAGTTCGACTGATCGCCATCGTCGCCTTCTTCGCGTGGCGCGTACGCCACAAGAACCGCGACGGCGCGTGGCTGTGGACAATGTCCATGGCCGGCGACGTCTGGTTCGGCTTCTCGTGGGCGCTCAACCAGCTCCCGAAGCTGAACCCCATCAAGCGCGTCGCGGACCTCGCCGCCCTCGCCGACCGGCAGCAGCACGGCACCTCCGGCGGCGGCGAGCTCCCCGGCGTCGACGTCTTCGTCACCACCGTCGACCCCGTCGACGAGCCGATCCTCTACACCGTGAACTCCATCCTCTCCATCCTCGCCGCCGACTACCCGGTGGACAGGTACGCCTGCTACCTGTCCGACGACGGCGGGACGCTGGTCCACTACGAGGCCATGGTGGAGGTCGCCAAGTTCGCTGAGCTGTGGGTGCCCTTCTGCCGGAAGCACTGCGTCGAGCCGAGGGCGCCGGAGAGCTACTTCGCGATGAAGACGCAGGCGTACAGGGGCGGCGTCGCCGGCGAGCTGATGAGCGATCGCCGCCGCGTGCGGCGAGAGTACGAGGAGTTCAAGGTCAGGATCGACTCGCTGTTCAGCACCATTCGCAAGCGATCTGACGCGTACAACAGAGCGAAGGATGGCAAAGATGACGGTGAAAACGCGACATGGATGGCTGATGGGACGCATTGGCCCGGCACATGGTTTGAGCCGGCGGAGAATCACCGGAAAGGGCAACACGCTGGGATTGTTCAGGTTTTACTGAACCATCCCACCAGTAAGCCACGGTTTGGAGTGGCGGCGAGTGTTGACAACCCGTTGGACTTCAGCGGCGTGGACGTGCGGCTCCCCATGCTGGTGTACATCTCGCGCGAGAAGCGCCCCGGGTACAACCACCAGAAGAAGGCCGGCGCCATGAACGCGCTGCTCCGCGTGTCCGCGCTGCTGTCGAACGCGCCCTTCATCATCAACTTCGACTGCGACCACTACGTCAACAACTCGCAGGCGTTCCGTGCGCCGATGTGCTTCATGCTCGACCGGCGCGGCGGCGGCGACGACGTGGCGTTCGTCCAGTTCCCGCAGCGGTTCGACGACGTCGACCCGACGGACCGGTACGCGAACCACAACCGCGTCTTCTTCGACGGCACCACGCTCTCCCTCAACGGCCTCCAGGGCCCCTCCTACCTCGGCACCGGCACCATGTTCCGCCGCGCCGCGCTCTACGGCCTGGAGCCGCCGCGGTGGGGGGCGGCGGGGAGCCAGATCAAGGCCATGGACAATGCCAACAAGTTCGGCGCCTCGTCGACGCTAGTCAGCTCGATGCTGGACGGCGCCAACCAAGAACGGTCGATCACGCCGCCCGTGGCGATCGACGGGTCGGTCGCCCGTGACCTCGCCGCCGTGACGGCGTGCGGCTACGACCTCGGGACGTCGTGGGGGAGAGACGCCGGGTGGGTGTACGACATCGCGACGGAGGACGTGGCGACCGGGTTCCGCATGCACCAGCAGGGATGGCGCTCCGTGTACACCTCCATGGAGCCCGCCGCGTTCCGCGGCACGGCGCCGATCAACCTCACCGAGCGCCTCTACCAGATCCTAAGGTGGTCGGGCGGCTCGCTCGAGATGTTCTTCTCCCACAGCAACGCGCTCCTCGCCGGCCGCCGCCTCCACCCGCTGCAGCGCATCGCCTACCTCAACATGTCGACCTACCCGATCGTCACCGTGTTCATCTTCTTCTACAACCTCTTCCCGGTGATGTGGCTCATCTCCGAGCAGTACTACATCCAGCAGCCATTCGGCGAGTACCTCCTCTACCTCGTCGCCATCATCGCCATGATCCACGTGATCGGCATGTTCGAGGTGAAGTGGTCGGGCATCACGGTGCTGGACTGGTGCCGCAACGAGCAGTTCTACATGATCGGCTCCACGGGGGTGTACCCGACGGCGGTGCTGTACATGGCGCTCAAGCTCTTCACCGGGAAGGGCATCCACTTCAGGCTCACGTCGAAGCAGACGACGGCCAGCTCCGGCGACAAGTTCGCCGACCTGTACACCGTGCGGTGGGTGCCTCTGCTGATCCCGACCATCGTCGTCCTGGCCGTGAACGTCGGCGCCGTCGGGGTGGCGGTCGGCAAGGCAGCGGCGTGGGGGTTGCTCACCGAGCAGGGGCGGTTCGCGGTGCTCGGGATGGTGTTCAACGTGTGGATCCTGGCGCTCCTCTACCCGTTCGCGCTGGGGATCATGGGGCAGCGGGGGAAGCGGCCGGCGGTGCTGTTCGTGGCGACGGTGATGGCCGTCGCCGCCGTGGCGATCATGTACGCCGCCTTCGGTGCGCCGTACCAAGCTGGGTTGTCAGGTGTCGCGGCTTCTCTCGGTAAAGCGGCGTCGCTGACCGGGCCATCTGGG

>OsCSLF6

ATGGCGCCAGCGGTGGCCGGCGGCGGAGGGAGGAGGAACAATGAGGGGGTGAACGGGAACGCGGCGGCGCCGGCGTGCGTGTGCGGGTTCCCGGTGTGCGCGTGCGCGGGGGCGGCGGCGGTGGCGTCGGCGGCGTCGTCGGCGGACATGGACATCGTGGCGGCGGGGCAGATCGGCGCCGTCAACGACGAGAGCTGGGTCGCCGTCGACCTCAGCGACAGCGACGACGCCCCCGCCGCCGGCGACGTCCAGGGCGCCCTCGACGACCGCCCCGTCTTCCGTACCGAGAAGATCAAGGGCGTCCTCCTCCACCCCTACCGGGTGCTGATCTTTGTGAGGCTGATCGCGTTCACACTGTTCGTGATATGGCGTATCGAGCACAAGAACCCGGACGCGATGTGGCTGTGGGTGACGTCGATCGCCGGCGAGTTCTGGTTCGGGTTCTCGTGGCTGCTCGACCAGCTCCCCAAGCTGAACCCGATCAACCGCGTCCCCGACCTCGCCGTCCTCCGCCGCCGCTTCGACCACGCCGACGGGACCTCCTCCCTCCCGGGGCTGGACATCTTCGTCACCACCGCCGACCCGATCAAGGAGCCCATCCTGTCGACGGCGAACTCCATCCTCTCCATCCTCGCCGCCGACTACCCCGTCGACCGCAACACCTGCTACCTCTCCGACGACTCTGGGATGCTCCTCACCTACGAGGCCATGGCGGAGGCGGCCAAGTTCGCGACGCTGTGGGTGCCCTTCTGCCGGAAGCACGCCATCGAGCCGCGCGGGCCTGAGAGCTACTTCGAGCTCAAGTCCCACCCCTACATGGGGAGGGCGCAGGAGGAGTTCGTCAACGACCGCCGCCGCGTCCGCAAGGAGTACGACGACTTCAAGGCCAGGATCAACGGCCTCGAGCACGACATCAAGCAGAGGTCCGACTCCTACAACGCCGCCGCCGGCGTCAAGGACGGCGAGCCCCGCGCCACCTGGATGGCCGACGGGTCGCAGTGGGAGGGCACCTGGATCGAGCAGTCGGAGAACCACCGCAAGGGCGACCACGCCGGCATCGTCCTGGTGTTGCTGAACCACCCGAGCCACGCACGGCAGCTGGGGCCGCCGGCGAGCGCCGACAACCCGCTGGACTTCAGCGGCGTGGACGTGCGGCTGCCGATGCTGGTGTACGTCGCACGTGAGAAGCGCCCCGGGTGCAACCACCAGAAGAAGGCCGGCGCCATGAACGCGCTGACCCGCGCCTCCGCCGTGCTCTCCAACTCCCCCTTCATCCTCAACCTCGACTGCGACCACTACATCAACAACTCCCAGGCGCTCCGCGCCGGCATCTGCTTCATGCTCGGCCGCGACAGCGACACCGTCGCGTTCGTCCAGTTCCCGCAGCGCTTCGAGGGCGTCGACCCCACCGACCTCTATGCTAACCACAACCGTATCTTCTTCGACGGCACGCTCCGTGCCCTCGACGGGCTGCAGGGGCCTATCTACGTCGGCACCGGGTGTCTCTTCCGCCGCATCACGCTGTACGGGTTCGAGCCGCCGAGGATCAACGTCGGCGGACCGTGCTTCCCGAGGCTCGGTGGGATGTTCGCCAAGAACAGGTACCAGAAGCCTGGGTTCGAGATGACCAAGCCTGGTGCCAAGCCGGTGGCGCCGCCGCCGGCGGCGACGGTGGCGAAGGGGAAGCACGGGTTCCTGCCGATGCCCAAGAAGGCGTACGGCAAGTCGGACGCGTTCGCCGACACCATCCCGCGCGCGTCGCACCCGTCGCCGTACGCGGCGGAGGCGGCGGTGGCGGCCGACGAGGCGGCGATCGCGGAGGCCGTGATGGTGACGGCGGCGGCGTACGAGAAGAAGACCGGGTGGGGGAGCGACATCGGGTGGGTGTACGGCACGGTGACGGAGGACGTGGTGACCGGCTACCGGATGCACATCAAGGGGTGGAGGTCGCGCTACTGCTCCATCTACCCGCACGCGTTCATCGGGACGGCGCCGATCAACCTGACGGAGAGGCTGTTCCAGGTGCTCCGGTGGTCGACGGGTTCGCTGGAGATCTTCTTCTCGAGGAACAACCCGCTGTTCGGGAGCACGTTCCTGCACCCGCTGCAGCGCGTGGCGTACATCAACATCACCACCTACCCGTTCACGGCGCTGTTCCTCATCTTCTACACCACCGTGCCGGCGCTGTCGTTCGTGACGGGGCACTTCATCGTGCAGAGGCCGACCACCATGTTCTACGTCTACCTCGCCATCGTGCTCGGGACGCTGCTCATCCTCGCCGTGCTGGAGGTGAAGTGGGCGGGGGTCACCGTGTTCGAGTGGTTCAGGAACGGGCAGTTCTGGATGACGGCCAGCTGCTCCGCCTACCTCGCCGCCGTGCTGCAGGTGGTCACCAAGGTGGTGTTCCGGCGGGACATCTCGTTCAAGCTCACCTCCAAGCTCCCCGCCGGCGACGAGAAGAAGGACCCCTACGCCGACCTGTACGTGGTGCGGTGGACGTGGCTCATGATCACCCCCATCATCATCATCCTCGTCAACATCATCGGCTCCGCCGTCGCCTTCGCCAAGGTGCTCGACGGCGAGTGGACGCACTGGCTCAAGGTCGCCGGCGGCGTGTTCTTCAACTTCTGGGTCCTCTTCCACCTCTACCCCTTCGCCAAGGGCATCCTCGGGAAGCACGGCAAGACGCCGGTGGTGGTGCTCGTCTGGTGGGCCTTCACCTTCGTCATCACCGCCGTGCTCTACATCAACATCCCCCACATCCATGGCCCCGGCCGCCACGGCGCCGCCTCACCATCCCACGGCCACCACAGCGCCCATGGCACCAAGAAGTACGACTTCACCTACGCCTGGCCA

>OsCSLF7

ATGCCGCCGTCAGCAGGCTTGGCCACTGAGAGCTTGCCGGCGGCGACATGCCCGGCCAAGAAGGATGCCTATGCCGCGGCGGCGTCGCCGGAGTCCGAGACGAAGCTGGCCGCCGGCGACGAGAGGGCGCCGCTCGTCCGGACGACTCGCATCTCGACAACTACCATCAAGTTATACAGGCTCACCATCTTTGTTCGCATCGCCATCTTCGTGCTCTTCTTCAAGTGGAGAATCACCTACGCTGCTCGCGCCATCAGCTCCACCGACGCCGGCGGCATCGGCATGAGCAAGGCGGCGACATTTTGGACGGCGTCCATCGCCGGCGAGCTCTGGTTCGCGTTCATGTGGGTGCTCGACCAGCTGCCCAAGACGATGCCCGTCCGGCGCGCCGTCGACGTCACGGCGCTGAACGACGACACGCTGCTCCCGGCGATGGACGTGTTCGTCACCACCGCCGACCCCGACAAGGAGCCGCCGCTCGCCACGGCGAACACCGTGCTGTCCATCCTCGCCGCGGGCTACCCCGCCGGCAAGGTGACGTGCTACGTCTCCGACGACGCCGGCGCGGAGGTGACACGCGGGGCGGTCGTGGAGGCGGCCCGGTTCGCGGCACTGTGGGTGCCCTTCTGCCGGAAGCACGGCGTCGAGCCGAGGAACCCGGAGGCGTACTTCAACGGCGGCGAGGGTGGCGGTGGTGGCGGCAAGGCGAGGGTGGTGGCGAGGGGGAGCTACAAGGGGAGGGCGTGGCCGGAGCTGGTGCGCGACAGGAGGCGGGTGCGCCGCGAGTACGAGGAGATGCGGCTGCGGATCGACGCGCTGCAGGCCGCCGACGCGCGCCGCCGGCGCTGCGGCGCGGCCGATGACCACGCCGGAGTTGTGCAGGTACTGATCGATTCTGCTGGGAGCGCGCCACAGCTCGGCGTCGCGGACGGGAGCAAGCTCATCGACCTCGCCTCCGTCGACGTGCGCCTCCCGGCGCTTGTGTACGTGTGCCGCGAGAAGCGCCGCGGCCGCGCACACCACCGGAAGGCCGGCGCCATGAACGCGCTGCTGCGCGCATCCGCCGTGCTCTCGAACGCGCCCTTCATCCTCAACCTCGACTGCGACCACTATGTCAACAACTCGCAGGCCCTCCGCGCCGGCATTTGCTTCATGATCGAACGCCGCGGCGGCGGCGCCGAAGACGCCGGCGATGTCGCGTTCGTCCAGTTCCCGCAGCGGTTCGACGGCGTCGATCCCGGCGACCGCTACGCCAACCACAACCGCGTCTTCTTCGACTGTACCGAGCTTGGCCTCGACGGCCTCCAGGGCCCCATCTACGTCGGCACCGGCTGCTTGTTCCGCCGCGTCGCGCTCTACGGCGTCGACCCACCGCGCTGGAGATCGCCCGGCGGCGGTGTCGCCGCGGACCCTGCCAAGTTCGGCGAGTCGGCGCCGTTCCTAGCCTCCGTTCGGGCGGAGCAGAGTCACAGTCGCGACGACGGCGACGCCATTGCCGAGGCGAGTGCGCTCGTGTCGTGCGCGTACGAGGACGGGACGGCGTGGGGCAGGGACGTCGGCTGGGTGTACGGCACCGTGACGGAGGACGTGGCCACGGGCTTCTGCATGCACCGGCGAGGGTGGCGCTCCGCCTACTACGCCGCCGCGCCCGACGCGTTCCGCGGCACGGCGCCGATCAACCTCGCCGACCGCCTCCACCAGGTGCTCCGCTGGGCGGCGGGCTCCCTCGAGATCTTCTTCTCCCGCAACAACGCACTCCTCGCCGGCGGCCGCCGCCGCCTCCACCCGCTGCAGCGCGCCGCCTACCTCAACACGACGGTGTACCCGTTCACGTCGCTCTTCCTCATGGCCTACTGCCTCTTCCCGGCGATCCCGCTCATCGCCGGCGGCGGCGGCTGGAACGCCGCGCCGACACCGACGTACGTCGCGTTCCTGGCGGCGCTGATGGTGACGCTCGCGGCGGTGGCCGTGCTGGAGACGAGGTGGTCGGGGATCGCGCTGGGTGAGTGGTGGCGGAACGAGCAGTTCTGGATGGTGTCCGCGACGAGCGCGTACCTCGCCGCGGTGGCGCAGGTGGCGCTCAAGGTCGCGACGGGGAAGGAAATATCGTTCAAGCTGACCTCGAAGCATCTCGCGTCGTCGGCGACGCCGGTCGCCGGTAAGGATAGGCAGTACGCGGAGCTGTACGCCGTGAGGTGGACGGCGCTGATGGCGCCGACGGCGGCGGCTCTGGCAGTGAACGTGGCGTCGATGGCGGCGGCGGGTGGTGGTGGCCGGTGGTGGTGGTGGGACGCTCCGTCGGCGGCGGCGGCGGCGGCGGCGGCACTCCCGGTGGCGTTCAACGTGTGGGTGGTGGTGCATCTCTACCCGTTCGCGCTCGGGCTGATGGGTCGCCGGAGCAAGGCGGTGCGCCCCATTCTGTTCCTGTTCGCCGTCGTCGCCTACCTCGCCGTCCGCTTCCTTTGTCTCTTGTTACAATTCCATACGGCT

>OsCSLF8

ATGGCGGCCAACGGCGGCGGCGGCGGCGCCGGTGGCTGCAGCAATGGTGGCGGTGGCGGCGCCGTGAACGGCGCGGCGGCGAATGGCGGCGGCGGCGGAGGAGGCGGCAGTAAGGGCGCGACGACGAGGAGGGCGAAGGTCAGCCCGATGGACAGGTACTGGGTGCCCACCGACGAGAAGGAGATGGCGGCGGCGGTCGCCGACGGCGGCGAGGACGGCCGGCGGCCGCTGCTGTTCCGGACGTTCACGGTCAGGGGCATCCTCCTCCACCCCTACAGGTTATTGACATTGGTCAGATTGGTTGCTATCGTCCTATTTTTCATATGGCGTATCAGGCACCCGTACGCCGATGGCATGTTTTTCTGGTGGATATCTGTGATTGGAGATTTTTGGTTTGGTGTTAGTTGGCTGCTAAACCAAGTGGCAAAGCTGAAACCGATCAGGCGTGTTCCCGATCTTAACCTCTTACAACAACAGTTTGATCTTCCTGATGGAAACTCCAACCTCCCTGGCCTTGATGTATTCATCAACACCGTCGATCCCATAAATGAGCCTATGATATACACTATGAACGCCATTTTATCCATTCTTGCAGCAGACTACCCAGTTGACAAGCATGCTTGCTATCTTTCGGATGATGGTGGATCGATCATCCATTATGATGGTTTACTTGAGACTGCAAAGTTTGCTGCGTTATGGGTTCCCTTTTGCCGAAAACATTCCATTGAGCCTAGGGCCCCTGAGAGCTATTTTGCAGTGAAGTCACGTCCATACGCTGGAAGTGCACCAGAGGATTTTCTCAGTGACCACAGATACATGCGTAGGGAGTATGATGAGTTCAAGGTACGCTTAGATGCGCTTTTTACTGTCATTCCCAAACGGTCAGATGCATACAACCAGGCACATGCCGAAGAAGGTGTGAAGGCAACCTGGATGGCAGATGGGACAGAGTGGCCTGGTACATGGATTGATCCATCTGAGAACCATAAGAAAGGAAATCACGCTGGAATTGTTCAGGTTATGTTGAACCATCCGAGCAATCAACCTCAACTTGGTCTACCAGCAAGCACTGACAGCCCTGTGGACTTCAGCAATGTTGATGTGCGCCTTCCTATGCTTGTATACATAGCCCGCGAGAAGCGCCCAGGCTATGACCACCAAAAGAAGGCAGGTGCCATGAACGTGCAGCTGCGAGTATCTGCCCTCCTCACCAATGCACCATTCATCATCAACTTTGATGGTGACCACTATGTCAACAACTCGAAGGCCTTCCGTGCTGGTATATGTTTCATGCTCGATCGACGTGAAGGTGACAACACTGCCTTTGTCCAATTCCCCCAACGTTTTGATGACGTTGACCCAACTGATAGGTATTGCAATCACAACCGAGTCTTCTTTGATGCTACTTTGCTCGGCCTCAATGGTATCCAGGGCCCCTCTTATGTTGGCACCGGTTGCATGTTCCGCCGAGTCGCACTGTATGGTGTTGACCCACCTCGTTGGAGACCCGATGATGGCAATATTGTGGATAGCTCCAAAAAGTTCGGCAACTTGGACTCCTTCATCAGCTCAATACCTATAGCAGCAAACCAAGAACGCTCAATCATATCACCACCTGCCCTTGAAGAGTCTATCCTGCAGGAGTTGAGTGATGCCATGGCATGTGCATATGAGGATGGGACTGACTGGGGCAAGGATGTTGGTTGGGTTTACAATATTGCAACCGAGGATGTGGTGACTGGTTTCCGATTGCATCGGACAGGGTGGCGCTCAATGTACTGCCGCATGGAGCCTGATGCATTCCGCGGCACTGCACCAATCAACCTCACTGAGCGCCTCTACCAGATTCTGCGCTGGTCAGGTGGCTCCCTTGAGATGTTCTTCTCACATAACTGCCCACTCCTTGCTGGCCGCCGACTCAACTTTATGCAACGAATTGCTTACATTAACATGACAGGCTACCCAGTTACATCAGTCTTCCTTTTGTTCTATCTCCTCTTCCCTGTCATATGGATCTTTCGCGGCATATTCTACATACAGAAGCCATTTCCTACATATGTATTGTACCTTGTGATCGTCATATTTATGTCAGAAATGATCGGTATGGTTGAGATCAAGTGGGCGGGGCTAACACTACTGGACTGGATCCGCAATGAACAGTTCTACATTATTGGAGCAACAGCTGTTTACCCGCTTGCAGTCCTGCACATAGTGCTGAAGTGTTTTGGTTTGAAGGGTGTCTCATTCAAGCTGACAGCAAAACAAGTAGCAAGCAGCACCAGCGAGAAGTTTGCAGAACTGTATGATGTTCAATGGGCACCATTGTTGTTCCCGACGATAGTGGTGATAGCAGTGAATATCTGTGCCATTGGCGCGGCAATAGGCAAGGCTCTCTTTGGAGGATGGTCACTGATGCAGATGGGAGATGCGTCGCTCGGGCTGGTATTCAACGTGTGGATCCTGCTGCTGATATATCCATTTGCACTGGGTATCATGGGAAGATGGAGCAAGAGACCCTATATCCTGTTCGTCTTGATTGTGATTTCATTTGTTATAATCGCATTGGCCGATATTGCCATCCAGGCAATGCGTTCTGGATCCGTTCGGCTCCACTTTAGACGGTCAGGTGGAGCCAACTTCCCTACAAGCTGGGGGTTT

>OsCSLF9

ATGGCGCTTTCGCCGGCGGCGGCTGGCCGTACCGGCCGAAACAACAATAACGACGCCGGCCTCGCGGACCCTCTGCTGCCGGCTGGCGGCGGCGGCGGCGGCGGTAAGGACAAGTACTGGGTGCCCGCCGACGAGGAGGAAGAGATTTGCCGCGGCGAGGACGGCGGCCGCCCGCCGGCGCCGCCGCTGCTGTACCGGACGTTCAAGGTCAGCGGCGTCCTCCTCCATCCCTACAGGTTACTGACCTTAGTCAGGCTGATCGCCGTCGTCCTCTTCCTCGCATGGCGCCTGAAGCACCGGGACTCCGACGCCATGTGGCTCTGGTGGATCTCGATCGCCGGCGACTTCTGGTTCGGCGTCACCTGGCTGCTCAACCAGGCCTCCAAGCTCAACCCCGTCAAGCGCGTCCCCGACCTCTCCCTCCTCAGGCGGCGCTTCGACGACGGCGGCCTCCCCGGCATCGACGTGTTCATCAACACCGTCGACCCCGTCGACGAGCCGATGCTCTACACCATGAACTCCATCCTGTCCATCCTCGCCACCGACTACCCCGCCGACCGGCACGCCGCCTACCTCTCCGACGACGGCGCGTCGCTGGCCCACTACGAGGGGCTGATCGAGACGGCGAGGTTCGCCGCGCTGTGGGTCCCGTTCTGCCGGAAGCACCGCGTCGAGCCGAGGGCGCCCGAGAGCTACTTCGCGGCGAAGGCGGCGCCGTACGCCGGGCCGGCGCTGCCGGAGGAGTTCTTCGGCGACCGCAGGCTCGTGCGCCGGGAGTACGAGGAGTTCAAGGCGCGGCTCGATGCGCTGTTCACTGACATTCCGCAACGATCGGAAGCGAGTGTTGGCAATGCAAACACCAAAGGCGCGAAGGCCACTCTGATGGCAGATGGGACCCCTTGGCCAGGGACATGGACCGAGCCAGCAGAGAATCACAAAAAAGGACAGCACGCCGGAATCGTTAAGGTAATGTTGAGCCATCCGGGTGAAGAGCCTCAGCTCGGCATGCCGGCGAGCTCCGGCCACCCTCTGGACTTCAGCGCCGTCGACGTGCGCCTCCCGATACTGGTCTACATCGCCCGGGAGAAGCGGCCGGGATACGACCACCAGAAGAAGGCCGGCGCCATGAACGCGCAGCTGCGCGTATCCGCGCTGCTGTCGAACGCGCCCTTCATCTTCAACTTCGACGGCGACCACTACATCAACAACTCCCAGGCGTTCCGCGCCGCCCTGTGCTTCATGCTCGACTGCCGCCACGGCGACGACACCGCATTCGTCCAGTTCCCGCAGCGCTTCGACGACGTCGACCCGACCGACCGGTACTGCAACCACAACCGCGTCTTCTTCGACGCCACGCTGCTCGGCCTCAACGGCGTCCAGGGCCCGTCCTACGTCGGCACCGGCTGCATGTTCCGCCGCGTCGCGCTCTACGGCGCCGACCCGCCGCGGTGGAGGCCGGAGGACGACGACGCCAAGGCGCTGGGCTGCCCCGGCAGGTATGGAAACTCGATGCCGTTCATCAACACGATACCGGCGGCGGCGAGCCAAGAACGGTCCATCGCGTCGCCGGCGGCGGCGTCGCTCGACGAGACGGCGGCCATGGCGGAGGTGGAGGAGGTGATGACGTGCGCGTACGAGGACGGGACGGAATGGGGCGACGGCGTCGGGTGGGTGTACGACATCGCGACGGAGGACGTGGTGACCGGCTTCCGGCTGCACCGGAAGGGGTGGCGGTCCATGTACTGCGCCATGGAGCCCGACGCGTTCCGCGGCACGGCGCCGATCAACCTCACGGAGCGCCTCTACCAGATCCTGCGGTGGTCGGGCGGCTCGCTCGAGATGTTCTTCTCCCGCAACTGCCCGCTCCTCGCCGGCTGCCGGCTGCGCCCCATGCAGCGCGTCGCGTACGCCAACATGACGGCGTACCCGGTCTCGGCGCTGTTCATGGTCGTCTACGACCTCCTCCCGGTGATCTGGCTCTCCCACCACGGCGAGTTCCACATCCAGAAGCCGTTCTCGACGTACGTCGCCTACCTCGTCGCCGTCATCGCCATGATCGAGGTGATCGGCCTGGTCGAGATCAAGTGGGCGGGGCTCACCCTGCTCGACTGGTGGCGCAACGAGCAGTTCTACATGATCGGCGCCACGGGGGTGTACCTGGCGGCGGTGCTGCACATCGTGCTCAAGAGGCTCCTCGGACTGAAGGGCGTGCGGTTCAAGCTGACGGCGAAGCAGCTGGCCGGCGGCGCGAGGGAGAGGTTCGCGGAGCTGTACGACGTGCACTGGTCGCCGCTGCTGGCGCCGACGGTGGTGGTGATGGCGGTGAACGTGACTGCCATCGGCGCGGCGGCGGGGAAGGCGGTCGTCGGGGGGTGGACGCCGGCGCAGGTCGCCGGCGCGTCGGCGGGGCTGGTGTTCAACGTGTGGGTCCTGGTGCTGCTCTACCCGTTCGCGCTCGGGATCATGGGGAGGTGGAGCAAGAGGCCGTGCGCGCTCTTCGCGCTGCTCGTGGCCGCGTGCGCGGCGGTCGCGGCGGGGTTCGTCGCCGTCCATGCCGTGCTCGCCGCCGGCTCCGCCGCGCCGTCCTGGTTGGGATGGTCTCGTGGCGCCACTGCCATTTTGCCGTCAAGCTGGCGACTTAAGCGGGGTTTC

>Sb01g021420

ATGGGCATCTTCGTGATGTTCTTCAAGTGGCGAATCAGCACTGCTCTTGCCATGATTAGCTCCACAGGCACCGATGACAAAAGTACAGTTCTTGGCATGTGGATGGTGTCCATCGCCGGGGAGCTCTGGTTTGCCCTGATGTGGGTGCTGGACCAGCTGCCCAAGATGCAGCCTGTCCGGCGCACCGTCTACGTCAGTGCACTCGATGAGTCGATGCTTCCGGCCATGGATGTATTCGTGACCACCGCCGACACCGAGAAGGAGCCGCCGCTGGTGACCGTGAACACCATCCTCTCCATCCTCGCGGCGGACTACCCCGCCGAAAAGCTCACTTGCTACGTCTCCGACGACGGTGGCGCTCAGATCACGCGCGACACGGTCGCCGAGGCTGCCCGGTTCGCTGGACTGTGGGTGCCGTTCTGCCGGAAGCACGCGGTGGAGCCGAGGAACCCAGAGGCCTACTTCAGCCCCGGCGCCGCCGCCAGTAATGGCGTCGTCAAGGCGAGGAGGGGTGACTATAAGGGAAGGGCGTGGCCGGAGCTGGCAAGGGACCGGCGGCGCGTGCGCCGGGAGTACGAAGAGCTGCGGCTGCGGATCGACGCTCTGCAAGCCGGAGACTTGCGGCGCCAGCAGCGGTCGACGTCGTCGCTGGCCGATGGAAGCTGCTGGCGGCGTGGCACCGCGGAAGATCATGCCGGAGCTGTCGAATTGGTACTCGATACTCCCGGCAGCACACCAGAACTTGGTGCCATCAGCGCCGTCGGTGGTGTCGGCAATCATCTGGACCTCAGCACCGTCGACGTGCGGGTTCCAGCGCTCGTGTACATGTGCCGGGAGAAGCGCCGCGGCCGCGTGCACCACGGCAAGGCCGGCGCCATGAACGCGCTGCTCCGTGCCTCGGCCGTGCTCTCCAACGCGCCCTTCCTCCTCAACCTTGACTGCGACCACTACGTGAACAACTCGCAGGCTCTCCGCGCCGGCGTCTGCCACCTGCTCGACGGCGAGGAGGGCAGCGACGTGGCGTTCGTCCAGTTCCCGCAGCGCTTCGACGGCGTCGACCCCGCCGACCGGTACGCCAACCACAACCGCGTCTTCTTCGACTGCACTGAACTCGGTCTCGACGGCCTTCAGGGACCCATCTACGTCGGAACCGGCTGCATCTTTCGCCGGGCGGCGCTATACGGCGTCGAACCGCCGCTCTGGAGACCACACGGCGACGACGCTGGCAAGGGCACTGAAGCCGACAAGTTGGCCGTGTCGACGCCGTTCCTTCGCTCGGTTCATGCGGTCCTGATGAACCAGCCGGAGTCGGAGGCGGAGCAATGGAACACGGTCGTTAGTATTTCACCACCAGCGTCCTCGTCGTTCGATGCGGCTGCCGTCGTCGAGGCAACGGCGCTCGTCTCGTGCGGCTACGAGGACGGGACGGCGTGGGGCCGTGACATCGGCTGGATGTACGGCAGCGTGACAGAGGACGTGGCCACGAGCTTCTGCATGCACCGGCGAGGGTGGCGCTCCGCCTACTGCGCCACCGCGCCGGACGCGTTCCGCGGCACGGCGCCCATCAACCTCACAGACCGGCTGTACCAGGTGCTCCGGTGGGCGGCGGGCTCCCTGGAGATACTCTTCTCCCGCAACAACGCCCTCCTCGCCGGCGGCGGCCGGCTGCACCCGCTGCAGCGCCTGGCGTACCTCAACACCACGGTGTACCCGTTCACCTCCATCTTTCTCATAGCCTACTGCGGCCTCTTCCCGGCGATCCAGCTCGTGACCGGGACCGGGACCGGGAAGGACGCCGCCACCACCGGCGGCGGCGCCTTCTTCTCAATAATCATCAGGCCGCCGTCTGCCACGTACATCGCCTTTGTGGCGGCTCTGATGCTGACGCTCGCGATGGTGGCCGTCCTGGAGGCGAGGTGGTCCGGCATAGCGTTGGTCGACTGGTGGCGGAACCAGCAGTTCTGGATGGTGTCTGCGACGAGCGCATACCTGGCGGCGGCGGTGCAGGTGGCGCTCAAGGTCGCGGCCGGGAAGGAGATATCGTTCAAGTTGACGTCCAAGCAGCGTGCGACGGCGAGCAATGGTGCGGCCAGTGTGAAAGACAGGTTCGCTGAGCTCTACGCCGTGAGGTGGACGGTGCTGATGGTCCCGACGGCCATGGTGCTAGCGGTGAACCTGACGTCCATGGTTGCAGCGATGATGGAAGGGGGGTCGTGGAGGGATGCCGGCCCAATGGAGGTGTTCGCTTTGGCGTTCAACGCGTACGTGGTGGTGCATCTCTACCCCTTCGCCCTGGGGCTCATGGGTCGCTGGAGCAACACGTTGAGCCCCGTGCTTGTCGCATTCTTCACCGTTCGATTACTTTGCTTCGTCCTCTATGTACGCATACTT

>Sb02g022011

ATGTCGTCCTCGCCGGTGCCCGGCGCCGGCGGTGCTGCTAGCGAGCACGCCGCCGGCGGCGGCGGCTTCAGAGAGCCGCTGCTCGCGAACGGCTGCGACGACGGCGGGTTTCACGACGGAGCACTGGCCGCGGTGGTCGTGGCCAATTATGCGCACGGCGGTGGGAGCAGGGGCAAGGAGAAGGACGCCGTGAAGAAGGCCAAGGACGGGTACTGGGTGGACGTCCACCACCGGCCGGCCGTGGCGGACGTGGAGAGCGGCGGCGGCGGCGACCGGCCGCTTCTCTTCTCTAACAAGAAGGTCATGGCGGCCCTCCTCTACCCGTACAGGGTACTGATTCTGGTGCGCCTCGTCGCCGTCATCCTATTCATCGCATGGCGCATCAAGCACAACAACTCCGACCTCCCAAAGTTCAGCCCCATCAAACGGACACCCGACCTCGCCGCGCTCCGGCGGCACTACGACGACCTCCCCGACGGCGGCGGCTCCATCCTCCCGGGCATCGACGTCTTCGTCACCACCGCCGACCCCGTGAGCGAGCCGGTGCTGTACACCATGAACTGCGTCCTCTCCATCCTGGCCACCGACTACCCCGTGGATCGGCTCACGTGCTACCTGACAGACGACAGCGGCGCGCTGGTTCTGTACGAGGCGCTGGTCGAGGCTGCGAGCTTCGCCGCCCTGTGGGTGCCCTTCTGCCGGAAGCACTCCGTCGAGCCGAGAGCGCCGGAGAGCTACCTCCAGCTGGAGGGGATGGTCTACAACGGGAGGTCGCCCGGTGAGTTCATGAATGACTACAGGCATGTGCAGAGGGAGTACGAGGAGCTGAAGGCGAGGCTGGAGATGCTCCCTAGCACCATCAAGGAGAGGTCAGATGTGTACAACAACAGCATGAAAGCAAAGGAAGGGGACCATGCTGGCATTGTCAAGATTGTGCAGAGCCACCCGAGCTGTGCCTGTGAGGCTCCTCCACCAGCAGAGGGCGGCAACCCCCTCAACTTTGACGGGGTGGACACTCGGGTCCCGATGGTCGTGTACGTGTCTCGCGAGAAGAGCCCAGGCCGCGAGCACAACAAGAAGGCGGGCAACCTGAACGCGCAGCTGCGCGTGTCGGCGCTGCTCTCCAACGCGCCCTTCACCATCAACTTCGACTGCGACCACTACATCAACAACTCGCAGGCGCTGCGAGCCGCCATGTGCTTCATGCTGGACGCCAGGGAGGGCGACCGCACCGGCTTCGTCCAGTTCCCGCAGCGGTTCCAGAACGTCGACCCCACGGACCGGTACGGCAACCACAACAGGGTCTTCTTCGACGGCGCCATGTACGCCCTCAATGGCCTCCAGGGCCCGACGTACCTCGGCACCGGCTGCATGTTCCGCCGCCTCGCGCTCTACGGCGTCGACCCGCCGCCTCGCCGGAGCCGGAGTTCCGACGAGGAGCACGGCCACGGCGGCGGCGTCACGGTCGACACTGACACTAGCAAGTTCGGTAACTCGGTGCTTTTCCTCGACTCGGTCTTGGCAGCTCTGAAGCAGGAGCGCCGCATTATCGCGCCTCCTGAGCTAGACGAGGCGGCGTTCCTCGCCGAGAAGACCACGGCCGTGTCGTCGTCGTACGACCAAGGGACGGACTGGGGCAGCAGCGTTGGGTACATATACAACATCGCGACCGAGGACATCGTCACCGGCTATCGCATCCACGGCCAAGGGTGGCGCTCCATGTACTGCAGCATGGAACGCGAGGCCTTCCAAGGCACTGCACCCATCAACCTCACCGAGCGCCTCTACCAGATCGTCCGGTGGTCCGGCGGATCCATGGAGGTGTTCTTCTCCCCTTACAACCCGCTGCTCTCCGGCCGCCGTCTCCACCTCCTGCAGCGGGCGGCGTACCTCAACTTCACCATCTACCCGGTCACGTCCGTCTTCGTCCTGCTCTACGCCTTCTGTCCGGTGATGTGGCTGATCCCGGCTGAAATCATCATCCAGAGGCCGTTCACTAGCTACGTGCTCTACCTCGTCGTCGTCGTCGGGCTGATCCACACCATCGGCGTCTTCGAGATAAAGTGGGCAGGGATCGCGTGGAACGACTGGTGGCGCAACGAGCAGTTCTTCATGATCGCCTCCATGAGCGCGTACCCGACGGCGGTGCTGCACATGGTGGTGAAGCCCATCACGGGGAAGGGCATCCACTTCAGGGTCACCTCGAAGCAGACGACGACGATGACGACGGCAGCTGACGACGACGACGACGGCGGCGGCGATGACAGGTACGCCGACATATATATGCGATGGGTGCCCATGCTGATCCCGCCGGCGGTGGTGCTGTTCTCCAACGTCATGGCCATCGGCGTGGCTCTGGGCAAAGCCGTCGTGGACAATGGAGTGTGGTCTGCGATGCAGAAGAGGCATGCGGCGCTGGGTATTCTCTTCAACGTGTGGATCATGGCGCTCCTCTATCCGTTTGGGTTGGCGGTTATAGGCCGGTGGAGCAAGAAACCTGGCATCCTCTTCGTCCTGTTGCCGCTGGCATTTGTGGTCATTGCAGCTGTGTATCTT

>Sb02g035980

ATGATGGCCGCGGCGGCAGTGACTCGCCGGGTCAATGCCATTGCCAGCCTCGACGTCGAGGCGACCAACGGCGTCGTCGCCGGCGAGGAGAGCAGGCGCAGCAGCTCGGCAGCTGACGACTCGCCGGTGGCCAAGCAGATTGACGATGCCGCCGCCAAGGACAATGATGTCTGGGTGGCCATTCAGGAGGGAGACATGCCTGCAGGCAACTCCAGCCAGCCCGTGCTGTTCCGGACCATGAAGGTCAAGGGCAGCATCCTGCATCCTTACAGGTTTGTGATTCTCCTGCGGCTGATCGCCATCATCGCGTTCTTCATATGGCGCATCCGGAACCGAAACCGCGACGGCGTGTGGATCTGGGCCATGTCCATGGCCGGCGACGTCTGGTTCGGCTTGTCATGGGTCCTCAACCAGCTCCCGAAGCTGAACCCCATCAAGCGTGTCCCGGACCTCGCCGCCATCAGAGACCAGCACGAGTCCACCAAGTCCAACTCCAATCTCCCCGGCATCGACGTCTTCCTCACCACCGTCGACCCGGTAGACGAACCCATACTGTACACGGTGAACTCCGTGCTCTCCATCCTCGCCACCGACTACCCGGTCGAGAAGTACGCCTGCTACCTCTCCGACGACGGCGGCACGTTGGTCCACTACGAAGCGATGCTCCAAGTTGCAAGCTTCGCCAAGCTATGGGCGCCGTTCTGCCGGAAGCATGGCGTCGAGCCGAGAGCGCCGGAGAGCTACTTCGGAGTGAAGAGGAGGCAGCCGTACACCGGAAGCATGCCGGAGGAGTTCACGAGTGATCACAGGCGCGTGCGCAGAGAATACGAAGAGTTCAAGGTGAGGATAGACTCTCTTTTCAGCACCATCTACCAGAGATCTGAAGCGTACAACAGGAAGCATGCCAAGGACGAAGATGGTGTGATGAAGGCGACGTGGATGGCTGACGGCACGCAGTGGCCTGGGACATGGATTGAGCAGGCTGAGAACCATAGAAAAGGACAGCACGCTGGAATTGTTAAGGTCATACTAAACCATCCGAGCCATAAACCACAGCTCGGTTCACCAGCAAGCACTGACAGTCCATTCAACTTCAGCAACGTCGACACACGCCTCCCCATGCTCGTCTACTTGTCCCGCGAGAAGCGCCATGGCTATAACCACCAGAAGAAAGCCGGCGCCATGAACGCGATGCTCCGGGCATCCGCCGTGCTCTCCAACGCGCCGTTCCTCATCAACTTCGACTGCGACCACTACATCAACAACTCGCAGGCTTTCCGCGCCAGCATGTGCTTCATGCTCGACCCACGCGACGGCGAGAACACCGCGTTCGTCCAGTTCCCGCAGCGCTTCGACGGCGTCGACCCCACGGACCGGTACGCCAACCACAACCGCGTCTTCTTCGACGGCACCATGCTCTCCCTCAACGGCCTGCAGGGGCCTTCCTACCTCGGCACCGGAACCATGTTCCGTCGCGCCGCGCTCTACGGCATGGAGCCACCGCGGTGGAGAGCAGCCGACGACGACGGCAATGGCAATGGCAACGGCAAAGAATATGGTAGATCGACATTGTTCATAAACTCAATGCTAGACGGCGCCCCGAACCAAGACCGACGATCTATCACGCCGGTGTTCGTCGACGGTGAAGAGTCGACGACGGTCAGCAGCGAGCTGCTGCTGGCGTCGCTGATGACGTGCGCTTACGAGGACGGGACTTCGTGGGGGAGAGACGCCGGGTGGGTGTACAACATCGCGACGGAGGACGTGGTGACCGGCTTCCGCATGCACCGGCAAGGGTGGCGCTCCGTCTACTGCTCCGTCGAGCCGGCGGCGTTCCGCGGCACCGCTCCGATCAACCTCACGGAGCGCCTCCTCCAGCTGCTCCGCTGGTCGGGCGGCTCCCTGGAGATGTTCTTCTCCCACAGCAACGCGCTCCTCGCCGCCGGCGCCGCCCGGATGCACCCGCTGCAGCGCGTGGCGTACCTCAACATGTCTACCTACCCGCTCGTCACCGTCTTCATCCTGGCCTACAACCTGTTCCCTCTGATGTGGCTCGTCTCGGAGCAGTACTACATCCAGAGGCCCTTCGGCGCGTACATCCTGTACCTGGCGGCGATCATCGCCATGATCCACGTGATCGGCATGTTCGAGGTGCGATGGGCGGGGCTGACGCTGCTGGACTGGTGCCGGAACGAGCAGTTCTACATGATCGGCGCCACGGGGGTGTACCCGACGGCGGTGCTGTACATGGCGCTGAAGCTCTTCACCGGAAAGGGCATCCACTTCAGGCTCACGTCGAAGCAGACGGCGGCGGAAGCCTGCTCCGGCGACAAGTTCGCCGACCTGTACGTCGTGAGGTGGGTGCCGCTGCTGGTCCCGACCGTCGCCGTTCTGGCCGTGAACGTCGCGGCGGTGGGCGTGGCGGTAGGCAAGGCGGCGACCTGGGGCCTGCTCACGGAGCAGGCGCAGCACGCGGTGCTTGGCATGGTGTTCAACGTCTGGATCCTTGTGCTTCTCTACCCGTTTGCGCTCGGGATCATGGGACACTGGGGGAAGAAACCGGCCATCCTGTTCGTTTTGCTGGTGATGGCCATTGGTACGGTCGCTGTCGTGTATATCAGCTTCAGTGCCACGTACCCAGTAGGATGGTCAGACATGGCAGCTTCTTCTCTTGGTACATCGGAATCGGTGATTGCAGCTGGGTCATCAGGG

>Sb02g035990

ATGACTTCGCCGGCGCTGTCCACCGGCGGCGCCGCTGTCATGGAAGATGGCGGCCTCATCGACCCACTTCTAGCGGCGCCGGATGGCAACGGCGCCACGAAGACGACGAAGACGAAGGAGTGCGGCGCCGAGGGCAAGTACTGGGTGGCCGCTGACGAGGCGGAGAGGCGGGCGGTGACCGAGTGCGGCGCTGAGGACGGCCGCCGCCCGCTGCTGTTCCGGACGTACAAGCTCAGGGGCGCCATCCTGCACCCCTACAGAGCACTAATCTTCGTGCGCCTAGTCGCGGTCCTCCTATTCTTCATCTGGCGCATCAGGAACAACAAATCCAATATTATGTGGTTCTGGGCTATGTCGGTTGTCGGGGACGCCTGGTTTGGATTCTCATGGCTCCTAAACCAGCTTCCCAAGTTCAACCCCATCAAGAGCATACCTGATCTTGATGCCCTCAGGCGATACTATGACCTTCCAGATGGCACCTCTAAACTCCCTAGCATCGACGTCTTTGTCACCACCGCTGATCCTATCGATGAGCCGATACTCTACACTATGAATTCCATCCTCTCTATCCTTGCCACTGATTATCCGATTGATAGACTTGCCTGCTACGTCTCTGATGATAGCGGGTCATTGATCCTTTATGAAGCATTGGTTGAGGTTGCAAAGTTTGCAATGTTGTGGGCTCCATTCTGTCACAAGCATTTCATTGAGCCAAGAGCACCAGAGAGGTATTTTGAGATGGAGGCACAACCACAAGGTGGAAGAGCAATGCAAGAGTTCTTAAATGACTACAAGAGGGTGCAAATGGAGTATGAAGAGTTTAAGGTGCGCTTGGGTAATCTTTCTGACACCATTCACAAGCGTTCGGATGTTTACAACAGCATGAGAACTTCAGAAGGAGATGCACAAGCAACTTGGATGGAAAATGGGATGCAGTGGCCAGGCACATGGATGGATCCAACAGAGAACCATAGAAAAGGACATCATAAGGGAATTGTTAAGGTTGTGTTGGATCAGCCAAGTCGTGGACATAACCATAGTCCCCAAGTCGGCGATGAAAATAAGTTTGACTTTGGTGTTGTTGGGTTGTGCCTACCAATGCTTGTGTATGTCTCTCGTGAAAAGAATCCAAGCTATGACCATAACAAGAAGGCGGGTGCATTGAATGCACAGCTGCGAGTCTCTGCTCTACTCTCCAATGCACAATTCATCATCAACTTTGACTGTGACCACTACATCAACAATTCTCAAGCTCTCCGTGCAGCAGTCTGCCTCATGCTTGATCAACGGAAAGGTGATAACACTGCCTTTGTTCAGTTCCCTCAACGCTTTGACAATGTTGACCCGACAGACCGTTATGGCAACCACAACCGAGTATTCTTTGATGGAACCATGCTTGCCCTGAATGGACTGCAAGGACCATCGTACCTTGGCACTGGATGCATGTTCCGCCGCATAGCACTCTATGGCATTGACCCACCTCATTATAGACAAGATAAAATCACACCTGAATCTAGTAAGTATGGCAAGTCCACTCCCTTAATAGACTCGATATCAAAAGCCATGAGAGAAGAAATGCTAACCACACAACCACCTTTTGATGACACATTTGTCACCGATACGAAGATGATTGTGGCAGCTTCTTATGATAAAGGGACTGACTGGGGTAAGGGTGTTGGCTATATTTATGACATAGCAACAGAGGACATAGTGACTGGATTTCGCATCCATGGGAAAGGGTGGAGTTCCATGTATTGCACAATGCAGCATGATGCATTTTGTGGCACAGCACCAATCAACCTAACAGAGCGCCTCCACCAAATTGTTCGTTGGTCCGGTGGGTCTCTAGAGATGTTCTTCTCTCACAACAACCCACTCATCGGTGGCCAACGACTACAACTTCTTCAACGTGTGTCCTACCTCAACATGACGGTCTACCCAGTTACATCACTTTTCATCCTGCTCTATTCTCTTTGCCCGGTAATGTGGCTCGTCCCCGATGAAATACACATCCAAAGGCCCTTCACTAGGTATGTTGTGTACCTTCTCATAATTATATTGATGATCCACATGATTGGCTGGCTCGAGATAAAGTGGGCTAGGTTCACATGGCTAGACTATTGGCGTAATGAGCAGTTCTTCATGATTGGGTCGACAAGTGCATACCCAATAGCACTATTTCACATGGCAAAGAAACTCCTCACAAAAAAGGGTATACACTTCAGGGTCACTTCCAAGCAAATGACCGCCAACACCAATGACAAGTTTGCCGATTTGTATGAGATGCGATGGACGTCGATGTTGATTCCCACGGTGTTTGTTTTGGTTGCCAATGTTGGTGCTGTTGGAGTGGCCATGGGCAAGGCCTTGGTGTACATGGGAGTATGGACGGTGTCGGAGAAGACTCATGCTGCACTTGGCCTTCTGTTCAATGTGTGGATCATGGTGCTCCTTTACCCCTTTGCACTGGCGATCATGGGAAGGTGGGCGAAGAGACCGATCATCCTGCTGCTTGATCCCAAGAACGGAAAAGTA

>Sb02g036010

ATGGCTTCACCGGCGCCGGCAGACGGTGACAATGGTGGCCTCACTGACCCACTTCTAGTGACCGCAACCGGCCATGGTGCCACCCCGAGGAAGGCTGGCCTCGGTGCCAAGGGCAAGTACTGGGTGGCCGCCGACAAGGCCGAGAGGATGGCTGCGAAGGAGGCCGGCGGTGAGGACGGCCGCGCGCTGCTGTTCCGGAAGTATAAGGTCAAAGGCGGCCTTCTGCACCCCTACAGGTTGCTGATCATCATCCGATTAATCGCTGTCCTCGTCTTCTTCGCATGGCGCATCAGGCACAACAAATCTGATATCATGTGGTTTTGGACAATGTCCATAGTCGGTGACGTCTGGTTCGGCTTCTCGTGGCTCCTCAACCAGCTCCCAAAGTTCAACCCCGTCAAGACCATCCCAGACCTTGCAGCCCTCAAGCAGCAGTTTGCCTTCTCTGAAGGCACCTCTAGGCTCCCCGGCATTGACGTTTTCGTGACCACTGCCGATCCCATTGATGAGCCCATACTCTACACCATGAATTGTGTCCTCTCCATCCTCGCTGTTGACTATCCAGTCGACAGGCTTGCCTGCTATCTCTCAGATGATAGCGGGGCACTCATCCTCTACGAGGCATTGGTCGAGGTCGGAAAATTTGCGCCTCTGTGGGTTCCATTTTGCCGCAAGTACTCCATTGAGCCAAGGGCGCCAGAAAGCTATTTTGAGCATGTGGCGCCACCACAAGCTGGAAGGGTGACGCAGGAGTTCTTGAATGATTATAGGAGGGTGCAAATGGAGTATGATGAGTTCAAGGTGCGCCTGGACATCCTCCCTGATGCCATCCGCAAGAGATCTGATGTTTATAGTAGCATGAGAGCTGCAGAAGGAGATCAAAAGGCAACTTGGATGGCAAATGGAACACAGTGGCCTGGCACATGGATTGATCCAACAGAAAACCATAGAAAAGGACACCACGCTCCTATTGCCAAGGTTGTGCTGCACCATCCAAGCAGTGGACAACATCTTGGTTCACAACCCATCACTGAAAGCAATCTCAGCATTACCACCACTGACGAGCGCCTCCCAATGCTTGTTTATGTCTCTCGTGAGAAGAACCCAAGTTATGACCACAACAAGAAGGCAGGTGCCCTGAATGCACAACTGCGGGCCTCTGCTCTCCTCTCTAATGCCCAACTCGTCATCAACTTTGACTGCGACCACTACATCAACAATTCTCAAGCCCTAAGCTCGGCTGTGTGCTTCATGCTAGATCAACGAGATGGTGATAACACCGCTTTCGTTCAGTTCCCACAGCGCTTTGACAATGTTGACCCCACAGACCGCTATGGTAACCACAATCGTGTCTTCTTTGATGGGACCATGCTTGCCCTTAATGGTTTGCAAGGACCCTCATACCTCGGCACTGGTTGCATGTTCCGTCGCTTAGCACTCTATGGTATTGACCCACCCCATTGTAGAGCAGAAAACATCACAGCCGAAGCTAGTAGGTTTGGTAACTCCACAATCTTTCTAGATTCAGTGTCAAAAGCCCTGAAAAATGACAGGTCAATCACACCACCACCAATTGATGACACATTCCTTGCTGAGTTAGAGAGGGTTGTGACATGTTCTTATGACCAAGGTTCTGACTGGGGCAAGGGTGTAGGGTACATCTATGACATAGCCACTGAAGATATAGTAACAGGATTTCACATTCACGGGCAGGGGTGGCGCTCTATGTATTGCACAATGGAGCATGATGCGTTTTGTGGTGTTGCACCGATCAACCTAACAGAACGCCTCCATCAAATTGTGCGTTGGTCTGGTGGGTCTTTAGAGATGTTCTTCTCCCACAACAACCCATTCATTGGTGGTCGCCGGATTCAACCTCTTCAACGTGTCTCCTACCTCAACATGACAGTCTACCCAGTCACATCAGTATTCATCCTGATCTATGCTCTAAGCCCAGTGATGTGGCTTATCCCTGACGAAGTATACATCCAGAGACCATTCACTAGGTATGTCGTGTACCTTATCATAATCGTCGTGATGATTCACATGATTGGCTGGCTTGAGATAAAGTGGGCGGGGGTCACATGGCTGGACTATTGGCGCAATGAGCAGTTCTTTATGATCGGCTCGACGAGTGCATATCCAATGGCAGTGTTGCACATGGTAGTGAACCTCCTCACAAAGAAGGGCATACACTTCAGGGTCACTTCCAAGCAAACAGCCGCAGATGACAATGACAAGTTTGCGGACTTGTATGATTTTCGATGGGTGCCAATGCTCATCCCAACAATGGCAGTTCTGGTCTGCAATGTTGGTGCCATTGGTGTAGCCCTGGGCAAAATAGTGGTAAACATTGAAACATGGACAGCGGCGAAGAAGATGCATGCTGCATTGGGTCTGCTGTTCAACATATGGATCATGTTTCTCCTCTACCCATTTGCACTAGCAATCATGGGGCGGTGGGCGAAGAGGCCAATCATCCTTGTAATCTTGCTGCCAGTTGTCCTTGTACTTGTTGCCATTTTGTATGTAGGTCTCCATATCTTACTTGCTGGTGTAATTCCACTC

>Sb02g036030

ATGTCGTCCTCGCCGGTGCCCGGCACCGGCGGTGCTGCTAGCGAGCACGATGCCGGCGGCGGCGGCTTCAGAGAGCCGCTGCTCGCGAACTGCGCCTGCGACGACGGCGGGTTTCACGACGGAGCACTGGCCGCGGTGGTCGTGGCCAATTCTGCGCACGGTGGCGGGAGCAGGGCCAAGGAGAAGGACGCCGTGAAGAAGGCCAAGGACGGGGTACTGATTCTGGTGCGCCTCGTCGCCGTCATCCTATTCATCGCATGGCGCATCAAGCACAACAACTCCGACGTCATGTGGTTCTGGGCGACGTCCGTCGTCGGCGACGTCTGGTTCGCGCTGTCGTGGCTGCTGTACCAGCTCCCAAAGTTGAGCCCCATCAAACGGACACCCGACCTCGCCGCGCTCCGGCGGCACTACGACGACCTCCCCGACGGCGGCGGCTCCATCCTCCCGGGCATCGACGTCTTCGTCACCACCGCCGACCCCGTGAGCGAGCCGGTGCTCTACACCATGAACTGCGTCCTCTCCATCCTGGCCACCGACTACCCCGTGGATCGGCTCACGTGCTACCTGACCGACGACAGCGGCGCGCTGGTCCTGTACGAGGCGCTGGTCGAGGCCGCGAGCTTCGCCGCCCTGTGGGCGCCCTTCTGCCGGAAGCACTCCGTCGAGCCCAGAGCGCCGGAGAGCTACTTCCAGCTGGAGGGGATGATCTACAACGGGAGGTCCCCCGGTGAGTTCATGAATGACTACAGACATGTGCAGAGGGAGTACGAGGAGCTGAAGGCGAGGCTGGAGATGCTCCCTAGCACCATCAAGGAGAGGTCAGACGTGTACAACAGCATGAAAGCTAAGGAAGGGGGTGCGCATGCGACTTGGATGGCTAATGGCACACAGTGGCCTGGCACTTGGATTGAGCCAGCAGAAAACCACAGGAAAGGAGACCATGCTGGCATTGTCAAGATTGTGCAGAGCCACCCGAGCAGTGATGCTCCTCCACCAGCAGAGGGCGGCAACAACAACAACATGAACCCCCTCAACTTTGACGGGGTGGACACTCGGGTCCCGATGGTCGTGTACGTGTCTCGCGAGAAGAGCCCAGGCCGCGAGCACAACAAGAAGGCGGGCAACCTGAACGCGCAGCTGCGCGTGTCGGCGCTGCTATCCAACGCGCCCTTCACCATCAACTTCGACTGCGACCACTACATCAACAACTCGCAGGCGCTGCGAGCCGCCATGTGCTTCATGCTGGACGCCAGGGAGGGCGACAGCACCGGCTTCGTCCAGTTCCCGCAGCGGTTCCAGAACGTCGACCCCACGGACCGCTACGGCAACCACAACAGGGTCTTCTTCGACGGCGCCATGTACGCGCTCAACGGCCTCCAGGGCCCGACGTACCTCGGCACCGGCTGCATGTTCCGCCGCCTCGCGCTCTACGGCGTCGACCCGCCGCCGCCTCGCCGGAGTTCCGACGTCGAGGAGCACGGCCACGGCGGCGTCACGGTCGACATTGACACTAACAAGTTCGGTAACTCGGTGCTCTTCCTCAACTCGGTCTTGGCAGCTCTGAAGCAGGAGCGCCGCATCGCGCCTCCTGAGCTAGACGAGGCGGCGTTCCTCGCCGAGATGACCATGGTGGTGTCGTCGTCGTACGACCAAGGGACGGACTGGGGCAGCAGCGTTGGGTACATCTACAACATCGCGACCGAGGACATCGTCACCGGCTATCGCATCCACGGCCAAGGGTGGCGCTCCATGTACTGCAGCATGGAACGCGAGGCCTTCCAAGGCACTGCACCCATCAACCTCACCGAGCGCCTCTACCAGATCGTCCGGTGGTCCGGCGGATCCATGGAGGTGTTCTTCTCCCCTTACAACCCGCTGCTCTCCGGCCGCCGGCTCCACCTCCTGCAGCGGGCGGCGTACCTCAACTTCACCATCTACCCGGTCACGTCCGTCTTCGTCCTGCTCTACGCCTTCTGTCCGGTGATGTGGCTGATCCCGGCTGAAATCATCATCCAGAGGCCGTTCACCAGCTACGTGCTCTACCTCGTCGGCGTCGTCGGGCTGATCCACACCATCGGCGTCTTCGAGATAAAGTGGGCAGGGATCGCGTGGAACGACTGGTGGCGCAACGAGCAGTTCTTCATGATCGCCTCCATGAGCGCGTGCCCGACGGCGGTGCTGCACATGGTGGTGAAGCCCATCACGGGGAAGGGCATCCACTTCAGGGTCTCCTCGAAGCAGACGACGACGACGGCGGCAGCTGACGACGACGGCGACGGCGGCGATGACAGATACGCCGACATGTACGAGATGCGGTGGGTGCCCATGCTGATCCCGCCGGCGGTGGTGCTGTTCTCCAATGTCATGGCCATCGGCGTGGCTCTGGGCAAAGCGATCGTGTACAATGGAGTGTGGTCTGCGGTGCAGAAGAGGCATGCGGCGCTGGGTATTCTCTTCAACGTGTGGATCATGGCGCTCCTCTATCCGTTTGGGTTGGCGGTTATAGGCCGGTGGAGCAAGAAACCTGGCATCCTCTTCGTCTTGTTGCCGCTGGCATTTGTGGTCATTGCAGCTGTGTATATTGGTGTTCATTTTTTTCTTGTCAAATTTCTTCCATTTATGGTGATA

>Sb02g036000

ATGGCTTCGCCGGCGCCGGCAGCCGACGGTGCCGTGAATGCGGCCAATGGTGGCCTCACTGACCCACTTCTAGTGAGCGCGAACGGCCACGGTGCCGCTCCGAGGAAGGCTGCCCATGGCGCCAAAGGCAAGTACTGGGTGGCCGCCGACAAGGCCGAGAGGCGGGCTGCGAAGGAGGCCGGCGGTGAGGACGGCCGCGCGCTGCTGTTCCGGAAGTACAAGGTCAAAGGCGCCCTTCTGCACCCCTACAGGTTGCTGATCATCATCCGATTAATCGCTGTCCTTGTCTTCTTCGCGTGGCGCATCAGGCACAACAAATCTGACATCATGTGGTTTTGGACAATGTCCATAGTTGGCGATGTTTGGTTCGGCTTCTCGTGGCTCCTCAACCAGCTCCCAAAGTTCAACCCTGTCAAGACCATCCCAGACCTGGCCGCCCTCAAGCGGCACTTTGGCTTCCCTGACGGCACCTCTAGGCTCCCCGGAATTGATGTTTTCGTCACCACTGCCGACCCCATTGATGAACCCATACTGTACACCATGAACTGTGTCCTCTCCATCCTCGCTGTTGACTACCCAGTCGACAGGCTTGCCTGCTATCTCTCAGATGACAGCGGGGCACTGGTTCTCTATGAGGCATTGGTCGAGGTCGGAAAGTTCGCACCTCTGTGGGTTCCATTCTGCCGCAAGTATTCCATTGAGCCAAGGGCACCAGAAAGCTATTTTGAGCATGTGGCGCCACCACAGGCTGGAAGGGTGACACAGGAGTTCTTGAATGATTATAGGAGGGTGCAAATGGAGTATGATGAGTTTAAGGTGCGCTTGGACAACCTCCCTGATGCCATCTGCAAGAGATCTGATGTTTACAATAGCATGAGAGCTGCAGAAGGAGATCAAAAGGCAACTTGGATGGCAAATGGGACGCAGTGGCCTGGCACATGGATTGATCCAACAGAAAACCATAGAAAAGGACATCATGCTCCAATTGCCAAGGTTGTGCTGGAGCATCCAAACCGTGGACAACATCATGAAAGCAATCTCAGCATTGGCACCACTGACGAGCGCCTCCCAATGCTTGTTTATGTCTCCCGTGAGAAGAACCCAAATTATGACCACAACAAGAAGGCAGGGGCCCTGAATGCACAACTGCGAGCCTCTGCACTCCTCTCTAATGCCCAACTCATCATCAACTTTGACTGCGATCACTACATCAACAATTCTCAAGCCCTAAGTTCAGCTGTCTGCTTCATGCTAGATCAACGGGATGGTGACAACACCGCATTTGTTCAGTTCCCACAGCGCTTCGACAATGTTGACCCCACAGACCGCTATGGCAACCACAACCGTGTCTTCTTTGATGGGACCATGCTTGCCCTTAATGGTTTGCAAGGACCCTCATACCTTGGCACTGGTTGCATGTTCCGCCGCTTAGCACTCTATGGTATTGACCCACCCCACTGCAGAGCAGAGAACATCACAGCCGAAGCTAGCAGGTTTGGTAACTCCACAATCTTCCTAGATTCAGTGTCAAAAGCCCTGAAAAATGACAGGTCAATTACACCACCACCAATTGATGACACATTCCTTGCTGAGCTAGAGAGAGTTGTGACATGTTCTTATGACAAAGGTACTGACTGGGGCAAGGGTGTAGGGTACATCTATGACATAGCCACTGAAGATATAGTAACAGGATTTCGCATCCACGGGCAGGGGTGGCGCTCCATGTATTGCACAATGGAGCATGACGCGTTCTGTGGCGTTGCACCAATCAACCTAACTGAACGCCTCCATCAAATTGTTCGTTGGTCTGGTGGGTCTTTAGAGATGTTCTTCTCCCACAACAACCCATTCATAGGTGGTCACCGGATTCAACCCCTTCAGCGCGTCTCCTACCTCAACATGACAGTCTACCCAGTCACATCAGTCTTCATCCTGATCTATGCTCTAAGCCCGGTGATGTGGCTTATCCCTGATGAAGTATACATCCAGAGACCATTCACTAGGTATGTCGTGTACCTTCTCGTAATCATAGTGATGATTCACATGATTGGCTGGCTTGAGATAAAGTGGGCGGGGGTCACATGGCTGGACTATTGGCGCAATGAGCAGTTCTTTATGATCGGCTCAACCAGCGCATATCCAATGGCAGTGCTGCACATGGCAGTGAACCTCCTCACAAAGAAGGGCATACACTTCAGGGTCACTTCCAAGCAAACAGCCGCAGATGACAATGACAAATTTGCTGACTTGTATGATTTTAGATGGGTGCCAATGCTCATCCCAACAATGACAGTTCTGATCTGCAATGTTGGTGCCATTGGTGTAGCCCTGGGCAAAACAGTGGTATACATTGGAACATGGACAGCGGCAAAGAAGATGCATGCAGCATTGGGTCTGTTGTTCAACATATGGATCATGTTTCTCCTCTACCCATTTGCACTAGCGATCATGGGGCGGTGGGCGAAGAGGCCAATCATCCTTGTAGTCTTGCTGCCAGTTGTCTTTGCACTTGTTGCGCTGTTGTATGCATTTGCTCGAACACCTGAAGGTTACAGCACGGAACAGCAGGACCCCACTCACACCAAACAATTGATTTCGTCAAACAAGAGGCAGAGGGCACGGAGGCTTACTTGTGAGCAGGGGAAGGCGAAGGTCGAGGGACACGAGCTGGCTAGGGAAATGGAATTGGAAGACAGCGGCAGCGGCAGCGGCGTTGCCAGAGACGATTCGCACCAAACGCCAGAACTGAGAACCGCCTGCGCGGCCGTGCGCGCGTGTTCCGCATTCAGATTCAGAATGCTCCGCCGCTTTTGGAGCAGGATACCAGCTTCGTGGGCTGAGGCCGTGCAT

>Sb02g036020

ATGGAAGGCATGCTCTCGCCGGCGGCTGGAAATAATGGAGGTCTCGCCAAGCCGCTGCTGGCCAACGACAACGGCAAGGCGGGGCGGGCCGGCGGCATCAGCGAGGACAAGTACTGGGTGCCCGTCGACGAGGAGGAGGAGGAGGAGGAGGAGGTGCTGGCGGCGGAGGAAGACGGCGGCAAGGACTGCCGCCGCCGGCCGCTGCTGTACCGGACGTTCAAGGTTAAAGGCATCCTCCTGCAGACTTACAGGCACCGGAACTCGGACAGCATGGTCCTCTGGTGGGTCACGGTGGTCGGCGACTTCTGGTTCGCCGTCAGCTGGCTGCTGAACCAGGCCTCCAAGCTCAACCCCATCCGGCGCGTCCCCAACCTCGCGCTCCTCAACCAACACTTTGATCCTCCCACGGCCACTCCCAGCGGCGGTGGCAGCTCCTGCAGCCAGCTCCCGGGCGTCGACGTGTTCATCAACACCGTGGACCCCGTGGACGAGCCCGTGCTCTGCACCATGAACTCCGTCCTGTCCATCCTCGCCACGGACTACCCAGTGGACAGGCACGCGACGTACCTCTCCGACGACGGCGGGTCGCTCGTCCACTACGAGGCGCTGCTCGAGACGGCGAAGTTCGCCGCGCTGTGGACGCCGTTCTGCCGGAAGCACCGCGTCGAGCCGAGGGCACCCGAGAGCTACTTCGCGGCGACGGCGGACGGGCCGTACGCTGGGGACGCGCCAGGGGAGTTCGTCGGCGACCGTCGGCACGTCCGCCAGGAGTACGAGGAGTTGAAGGCTCGGGTGGATGCGCTGTTCACCGTCATTCCCCAGAGGTCGGAGGCCAAACAAGGTGGTGACCATGCGACGTACATGGCCGATGGGACGCATTGGGCTGGCACCTGGATTGAGCCGGCTGAAAACCACAAGAAAGGACACCACGCCGCCATTGTCCAGGTTATTTTGAACCATCCCGGCGACGAGCCTCAGCTTGGCACGCCGGCGAGCTCCTCCAGCGCTCTGGACTTCAGCGCCGTGGACGTGCGCCTCCCTATGCTGGTGTACATCGCGCGTGAAAAGCGGCCGGGGTACGACCACCAGAAGAAGGCCGGCGCCATGAACGTGCAGCTGCGTGTCTCGGCGCTGCTCTCCAACGCGCCCTTCATCATCAACTTCGACTGCGACCACTACATCAACAACTCCGGTGCGTTCCGCGCCGCCATGTGCTTCATGGTCGACCCTCGCCACGGCGACGACACCGCCTTCGTCCAGTTCCCGCAGCGCTTCGACGACGTCGACCCTACGGACCGGTACTGCAACCACAACCGCGTCTTCTTCGACGCCACCTCCCTCGGCCTCAACGGCATCCAGGGGCCTTCCTACGTCGGCACCGGCTGCATGTTCCGGCGCGTCGCGCTCTACGGCGCCGACCCGCCCCGGTGGCAGCAGCCCGGCGACGGCGCCTCCAAGCTCCTGGACAACAACCCCCGCAGGCAGTTCGGCGGCTCCATGCCGTTCATCACCTCTGTGACCCTGGCCGCGCACCAGGAACGGCCCCTCACGCCGCCAGCGTCGCTCGACGACGAGCGGCTCGTGGCGGAGTTGGCCGACGTCGCGACCTGCGCGTACGAGGACGGGACGGAGTGGGGCGACGGCGTCGGGTGGGTGTACAACATCGCGACGGAGGACGTGGTGACCGGCTTCCGGGTGCACCGGAAGGGGTGGCGGTCCATGTACTGCGCCATGGAGCCCGACGCGTTCCGGGGCACGGCGCCGATCAACCTCACGGAGCGCCTCCACCAGATCCTGCGCTGGTCGGGGGGCTCCCTGGACATGTTCTTCTCCCGCAACTCGCCGCTCCTAGCCGGCCGGCGGCTCCACCCGATGCAGCGCGCCGCCTACACCAACATGACGGCCTACCCAATCTCGGCGGCTTTCATCTTCGTCTACGACCTTCTCCCGCTGATGTGGCTCCCCGGCGACGGCGAGTTCTACATACAGAAGCCGTTCCAGACCTACGCGCTGTACATGTTCGTCGGCATCGCCATGATGGAGGTGAGCGGCATGGTGGAGATCAAGTGGGCGGGGCTGACGCTGCTGGACTGGTGCCGGAACGAGCAGTTCTACATGATCGGCGCGACGGGCGTGTACCCGGCGGCGGTGCTGCACAGCCTGCTCAGGCTCGTCGGCCTCAAGGGCATTCCGTTCAAGCTGACGTCGAAGCTGGTGTCCGCGAGCGGCGGCGGCGTTGCCGCGGGGGAGAGGTTTGCGGAGCTCTACCAAGTGCAGTGGACGCCGCTGCTGGTGCCGACCGTGCTGGTGATCGCCGTGAACGTCGCCGCCATCGGCGTGGCCGTCGGCAGGGCGGCCGCCTTCGGGTGGTCGTTCGCGCAGGTCGCCGGCGCGGCGAGCGGGCTGCTTTTCAACGTGTGGGTGCTGCTGCTGCTGTACCCGTTCGCGCTCGGGATCATGGGGCGATGGAGCAAGAGGACCTACCTGCTCTTCGTCCTGCTCGTGGCCATGCTCGTTATCATCGCGTCCGCGTACGTCGCGGTCCTGGCGGTAGTTGCTCCAGGTTCTGTGGCGGCGCTC

>Sb02g036023

GCGGGCGGTACGGTGGAAGACAATCAGATGGGAGGTGCCAGCGGCGGGGGCGGCAATGCCAAAGTGATGAAGGGTTCCATGAAGGCGGAGGCGAAGGACGACAGGTACTGGGTGGATGTCCACCGAGAGGTAACAGCTGCGGCGGACCTGGAGAACGGCGGCGACGGTGGCCGGCCGCTCATGTTCCGAGAAAAGAAGGTCAAGCCAGCCCTCCTGTACCCCTACAGGACTTGCATCATGCCATGCTGTGGTTTTGGCCTCCGTCTGCACCTCGGACACACTAACAGGACACTAATTCTAATACGCCTCATCGCCGTTATCTTATTCATCGGATGGCGTATCAAGCACAATAACTCCGACGTCATGTGGTTTTGGACGACCTCTGTCGTCGCCGATGTCTGGTTCGCCTTCTCGTGGCTGCTCTACCAGATGCCGAAGTTCAATCCCATCAAAAGGTCCCCTGACCTGGATGCTCTACGACAGTACTACGACCTCCCTGATGGCGACTCCATCCTTCCAGCCATTGATGTCTTTGTCACCACCGCTGACCCCATCGATGAACCGGTGTTGTACACTATGAATTCTATCCTTTCCATCCTCGCCGTTGACTACCCCATTGATAGGTATGCATGCTACCTATCTGATGATAGTGGCACACTGATCGAATATGATGCTTTGGCTGAGACGGCAAAATTTGCCGCTTTGTGGGCACCCTTCTGCCGGAAGCACTCAATTGAGCCGAGAGCTCCAGAAAGCTACTTCCAGCGAGAGGGGATGATCTACAACGGCAAATCACCGAGTGAGTTCATTAATGACTATAGGCATGTGAACGTGGAGTATCAACGGTATAAGGCAAGGTTGGAAATGCTCACCAGCACTATTAGAGAGAGATCGAACTTCTATAACAATATCAAAACAACAAAAGGGGATGTAAACGCGACTTGGATGGCGAACGGGACACAGTGGCCAGGAACATGGCTTGAGCCAATAGACAATCATAGGAAAGGACATCATGAAGGAGTTGTTCAGGTTGTGCTAGAGCCTCCAAATGGTGGTAAGACACAACATGACAATATTGTGAACCCACTCAATTTTGATGGCATTGATGCACGCCTCCCAATGCTTGTCTACATGGCTCGTGGAAAGAGCCCATGTTATGACCACAATAAGAAGGCGGGTAACTTGAATGCCCAACTACGGGTTTCTGCTCTACTCTCTAATGCACCCTTTGTCATCAATTTCGACTGTGATCACTACATCAATGACTCTCGAGCTTTGCAAGCTGCTATGTGCTTCATGCTAGACTCAAGGGAAGGAGATAACATAGCCTTCGTTCAATTCCCCCAACGTTTTGAAAATGTTGACCCAACAGACAGATATGGAAACCACAATAGGGTTTTCTTTGATGGAGCTATGTATGCCCTAAATGGTATCCAAGGACCTTCTTATCTCGGCACTGGTTGCATGTTTCGCCGCCTTGCGCTCTATGGTATTGACCCACCACGCTGGAGACCTAATGACATTCTGGTTGATAGTAGCAAGTTTGGTAACTCCATACCCTTCTTGAACTCAGTGTTACAATCCTTAAAACAAGAAAGCCACATCTCACCGCTAAACCTAGATGACTCATTTATTGCTGAGATGATGCTGGTCATATCATCTTCCTTTGATATAGGGACAGACTGGGGGAGGGGTGTTGGGTATATATATGAGATGGCAACTGAGGATATGGTGACTGGCTTTCGCATCCACAAGCAAGGGTGGCACTCCATGTATTGCACCATGGATGTGGATACATTCTGTGGCACTGCACCAATCAACCTAACTGAGCGTCTCTACCAAATTGTTCGATGGGCCGGTGGTTCTGTAGAGATGTTCTTCTCCCATAACAACCCATTATTAGCTGGATGTCGGCTCCACCCAATGCAACGGATTGTATACCTCAATTATAATATCTACCCTATCACATCACTTTTTCTCCTACTCTACGCATTATGCCCAGTGATGTGGCTTCTCCCCGAGGAAATACTGATCCAGAGACCATTTACTCGATATGTTGTATTCCTCATCATTATCATTGCATTAATTCATACCATAGGCATCATGGAGATAAAGTGGGCGGGTACCAAATGGCTAGATTGGTGGCGCAATGAACAGTTCTTCATGATTGCCTCATTGAGCGCGTACCCAACGGCGTTGTTGCACATTGTTGTGAAGCTCCTCACAAGGGGTAAGGGGATCCGCTTTAGGGTCACCTCGAAGCAAACAAAGGTAGAGGACAATGAAGACAAGTATGCTGAGATGTACGAGATGCGCTGGGTACCTATGTTGATCCCAGCTATGGTGGCCCTGTTCTCTAACACCATGGCTATCGGCGTGGCCATAGGAAAAGCAATCGTGTATGGTGGGGTATGGCCTAAGACACAACGTTTGCATGCGATGCTAGGCCTGTTGTTTAATGTGTGGCTCATGATTCTCCTCCAACCATTTGCTTTGGCACTCATAGGACGTTGGAGCAAGAAGCCTAGCATCCTTTTCATCTTGTTTCCAGTGGCATTTGTGGTCTTTGCACTGGTGTACATTTGTGTT

>Sb07g004110

ATGGCGCCGGGCGGCGGAGACGGCCGGCGCAACGGCGAGGGACAGCAGCAGGCGAACGGCAACAACAACAACAACAACAGCAACGCTAAGGCTAAGCACGGGTGCGTGTGCGGGTTCCCCGTGTGCGCGTGCGCCGGCGCGGCCGCGGTGGCGTCCGCGGCCTCCTCCGCCGACATGGACCGCGTGGCCGCCGCGCAGACCGAGGGCCAGATCGGCGCCGTCAACGACGAGAGCTGGATCGCCGTCGACCTGAGCGACGACCTCTCCGGCGACGGCGGCGGCGCCGACCCCGGCGTCGCGATCGAGGACCGCCCCGTCTTCCGCACCGAGAAGATCAAGGGCATCCTCCTCCACCCCTACAGGGTGCTCATCTTCGTGCGCCTGATCGCGTTCACGCTGTTCGTCATCTGGCGTATCTCGCACCGCAACCCGGACGCGATGTGGCTGTGGGTGACGTCGATCGCGGGCGAGTTCTGGTTCGGCTTCTCCTGGCTGCTGGACCAGCTCCCCAAGCTGAACCCGATCAACCGCGTCCCGGACCTCGCGGTGCTCCGGCAGCGGTTCGACCGCGCCGACGGCACGTCCCGCCTCCCGGGCCTGGACATCTTCGTCACCACGGCGGACCCGTTCAAGGAGCCCATCCTGAGCACGGCCAACTCCATCCTCTCCATCCTTGCCGCCGACTACCCCGTGGAGCGCAACACGTGCTACCTCTCCGACGACTCCGGGATGCTGCTCACCTACGAGGCCATGGCGGAGGCCGCCAAGTTCGCCACCGTCTGGGTGCCCTTCTGCCGGAAGCACGGCATCGAGCCTCGTGGCCCCGAGAGCTACTTCGAGCTCAAGTCGCACCCCTACATGGGGAGGTCGCAGGAGGACTTCGTCAACGACCGCCGCCGTGTCCGCAAGGAGTACGACGAGTTCAAGGCCCGGATCAATGGCCTCGAGCATGATATCAAGCAGAGGTCCGACGCGTTTAACGCCGCTAGGGGGCTTAAGGACGGCGAGCCCAGAGCTACGTGGATGGCCGACGGGAACCAGTGGGAGGGCACATGGGTTGAGCCATCGGAGAACCACCGCAAGGGTGACCACGCCGGCATCGTCTATGTGCTTCTGAACCACCCGAGCCACAGCCGTCAGCTCGGCCCGCCGGCGAGCGCGGACAACCCGCTGGACTTCAGCATGGTGGACGTTCGCCTCCCCATGCTGGTGTACGTCTCCCGTGAGAAGCGGCCCGGGTTCAACCACGAGAAGAAGGCCGGCGCCATGAACGCGCTGACCCGCTGCTCCGCCGTGATCTCCAACTCGCCCTTCATCCTCAACCTGGACTGCGACCACTACATCAACAACTCGCAGGCGCTTCGCGCCGGCATCTGCTTCATGCTCGGCCGGGACAGCGACACGGTGGCGTTCGTGCAGTTCCCGCAGCGGTTCGAGGGCGTGGACCCCACGGACCTGTACGCCAACCACAACCGCATCTTCTTCGACGGCACGCTCCGGGCGCTGGACGGCATGCAGGGCCCCATCTACGTCGGCACCGGCTGCATGTTCCGCCGCATCACGCTCTACGGCTTCGACCCGCCGAGGATCAACGTCGGCGGGCCGTGCTTCCCGTCGCTCGGCGGCATGTTCGCCAAGACCAAGTACGAGAAGCCCGGGCTGGAGCTCACCACCAAGGCCGCCGTCGCCAAGGGCAAGCACGGCTTCCTCCCGTTGCCCAAGAAGTCGTACGGCAAGTCGGACGCGTTCGTCGACACCATCCCGAGGGCGTCTCACCCGTCGCCGTTCCTGAGCGCCGACGAGGCCGCCGCCATCGTCGCCGACGAGGCCATGATCACCGAGGCCGTGGAGGTGTGCACGGCGGCGTACGAGAAGAAGACCGGCTGGGGCAGCGACATCGGCTGGGTGTACGGCACCGTCACCGAGGACGTGGTGACGGGGTACCGGATGCACATCAAGGGGTGGCGGTCTCGCTACTGCTCCATCTACCCGCACGCCTTCATCGGCACCGCCCCGATCAACCTGACGGAGCGGCTGTACCAGGTGCTCCGCTGGTCCACGGGGTCGCTGGAGATCTTCTTCTCCCGGAACAACCCGCTGTTCGGCAGCACGTTCCTGCACCCGCTGCAGCGCGTGGCGTACATCAACATCACCACCTACCCGTTCACGGCGCTGTTCCTCATCTTCTACACCACCGTGCCGGCGCTGTCGTTCGTGACGGGGCACTTCATCGTGCAGCGGCCGACCACCATGTTCTACGTGTACCTGGCCATCGTGCTGGGGACGCTGCTCATCCTGGCCGTCCTGGAGGTGAAATGGGCGGGCGTCACCGTCTTCGAGTGGTTCAGGAACGGGCAGTTCTGGATGACGGCCAGCTGCTCCGCGTACCTGGCCGCCGTGTGCCAGGTGCTGGTGAAAGTGGTGTTCCGGCGAGACATCTCCTTCAAGCTCACATCCAAGCAGCCCGCCGGCGACGAGAAGAAGGACCCCTACGCCGACCTGTACGTGGTGCGCTGGACCTGGCTCATGGTGACCCCCATCATCATCATCCTCGTCAACATCATCGGATCCGCCGTGGCGTTCGCCAAGGTGCTGGACGGCGAGTGGACGCACTGGCTCAAGGTGGCCGGCGGCGTCTTCTTCAACTTCTGGGTGCTGTTCCACCTCTACCCGTTCGCCAAGGGCCTCCTCGGGAGGCACGGCAAGACCCCCGTGGTGGTGCTCGTCTGGTGGGCATTCACCTTCGTCATCACCGCCGTGCTCTACATCAACATCCCCCACATCCATGGCCCCGGCGGCAAGCACGGCGGCGCGATCGGAAAGCACGGCGCCGCCCACCACGGCAAGAAGTTCGACCTCGACAACCTCTCCTACAACTGGCCG

>HvCSLF3

ATGGCGTCGGCGGCCGGTGCTGCTGGGTCAAATGCCAGCCTCGCCGCCCCGCTGCTGGCGAGCCGCGAGGGAGGTGCCAAGAAGCCGGTCGGTGCCAAGGGCAAGCACTGGGAGGCCGCCGACAAGGACGAGCGGCGGGCCGCCAAGGAGAGCGGCGGCGAGGACGGCAGGCCGCTGCTGTTCCGGACGTACAAGGTCAAAGGCACCCTCCTGCACCCATACAGGGCGCTAATCTTCATTCGCTTAATTGCGGTCCTTCTATTCTTCGTATGGCGCATCAAGCACAACAAATCCGACATCATGTGGTTTTGGACAATATCAGTCGTCGGGGACGTATGGTTCGGGTTCTCGTGGCTGCTCAACCAACTCCCAAAGTTCAACCCTATCAAAACCATACCTGATATGGTCGCCCTTAGGCGACAATACGATCTTTCAGATGGGACATCTACACTCCCGGGCATAGATGTCTTTGTCACCACCGCTGACCCAATCGATGAGCCGATACTATACACCATGAATTGTGTCCTTTCTATCCTTGCTTCTGACTATCCTGTCGATAGGTGTGCCTGCTATCTCTCAGATGATAGTGGAGCATTGATTCAATACGAGGCCTTAGTTGAGACCGCAAAGTTTGCTACTTTGTGGGTCCCATTTTGTCGGAAGCATTGCATTGAGCCAAGAGCCCCAGAAAGCTACTTTGAAATAGAGGCACCGTTGTACACTGGAACTGCACCAGAGGAGTTCAAGAATGATTATAGTAGTGTACATAAAGAGTATGATGAGTTCAAAGAGCGCTTGGACTCACTATCCGATGCTATTTCCAAGCGTTCTGATGCTTACAACAGCATGAAGACTGAGGAAGGAGATGCAAAGGCCACGTGGATGGCAAATGGGACACAATGGCCAGGATCATGGATTGACACAACGGAAATCCATAGGAAAGGACATCATGCCGGAATTGTTAAGGTTGTGTTGGACCATTCGATCCGTGGGCATAATCTTGGTTCACAAGAAAGCACCCACAACCTCAGCTTCGCCAACACCGATGAGCGCCTCCCGATGCTTGTGTATATCTCTCGTGGAAAGAACCCAAGCTATGACCACAACAAGAAAGCTGGTGCCTTGAATGCGCAATTGCGTGCCTCTGCACTACTCTCCAACGCACAATTCATCATCAACTTTGACTGCGACCACTACATCAACAACTCTCAAGCCCTACGTGCAGCTATGTGCTTCATGCTTGATCAAAGGCAAGGTGATAACACTGCCTTTGTTCAATTCCCTCAACGCTTCGACAATGTTGATCCATCAGACCGATATGGAAACCACAACCGTGTCTTCTTTGACGGCACAATGCTCGCCCTCAATGGCCTCCAAGGGCCATCTTACCTTGGCACTGGTTGCATGTTCCGCCGCATAGCACTTTATGGCATTGACCCACCTGACTGGAGACATGACAACATCATAGTTGATGATAAAAAGTTTGGTAGCTCCATACCCTTCCTAGATTCCGTATCAAAAGCCATAAACCAAGAAAGGTCTACCATACCTCCACCCATTAGTGAAACATTGGTGGCTGAGATGGAAAGGGTTGTGTCGGCTTCACACGATAAAGCCACTGGTTGGGGCAAGGGTGTTGGGTACATATATGACATAGCCACAGAGGATATCGTGACTGGTTTCCGCATCCATGGGCAAGGTTGGCGTTCCATGTATTGTACAATGGAGCGTGACGCCTTCTGTGGCATTGCACCAATCAACCTAACCGAGCGCCTCCACCAAATTGTGCGCTGGTCCGGTGGATCTTTAGAGATGTTCTTCTCACTAAATAACCCACTCATAGGTGGTCGCCGGATCCAAGCCCTTCAGCGTGTCTCCTACCTCAACATGACAGTCTACCCAGTCACATCACTCTTTATCCTACTCTATGCTCTCAGCCCAGTGATGTGGCTTATCCCTGATGAAGTATACATCCAGAGGCCATTCACCAAATATGTCGTGTTCCTTCTCGTGATCATTCTGATGATCCATATAATTGGGTGGCTCGAGATAAAATGGGCGGGGGTCACATGGTTGGATTACTGGAGGAATGAACAGTTCTTTATGATCGGGTCGACGAGTGCATACCCAGCAGCCGTGCTGCACATGGTGGTGAATCTCCTTACAAAGAAGGGTATACACTTCAGAGTTACTTCGAAGCAAACAACGGCAGACACCAATGACAAGTTTGCTGACTTGTATGACATGCGATGGGTGCCAATGTTAATCCCTACAACAGTGGTGCTGATTGCCAATGTTGGTGCAATCGGTGTAGCCATGGGTAAAACGATAGTATACATGGGAGCATGGACAATTGCACAGAAGACACATGCCGCATTGGGTCTGCTCTTCAACGTGTGGATCATGGTCCTGCTCTATCCGTTTGCATTGGCGATCATGGGACGGTGGGCAAAGAGGCCAGTCATCCTGGTGGTCTTGTTGCCGGTTGCCTTTACAATAGTTTGCCTTGTATATGTTTCTGTTCATATATTACTTCTTAGTTTTCTTCCATTT

>HvCSLF4

ATGGCCCCGGCAGTCACTCGCCGAGCCAACGCTCTCCGCGTCGAGGCCCCGGACGGCAATGCCGAGAGCGGGCGCGCCAGCCTAGCAGCAGACTCCCCCGCGGCCAAGCGGGCCATCGATGCCAAGGACGATGTGTGGGTGGCCGCGGCTGAGGGAGACGCGTCTGGAGCCAGCGCCGGCAACGGCGACCGGCCGCCGCTGTTCCGGACCATGAAGGTCAAGGGAAGCATCCTCCATCCTTACAGGTTCATGATCCTCGTGCGCTTGGTCGCCGTCGTCGCGTTCTTCGCGTGGCGCCTGAAGCACAAGAACCACGACGGCATGTGGCTCTGGGCCACGTCCATGGTCGCCGACGTCTGGTTCGGCTTCTCATGGCTCCTCAACCAGCTGCCCAAGCTCAACCCCATCAAGCGCGTCCCCGACCTGGCCGCCCTCGCCGACCAGTGCGGCTCCTCCGGCGACGCCAACCTGCCAGGCATCGACATCTTCGTCACCACCGTGGACCCCGTGGACGAACCCATCTTGTACACCGTGAACACCATACTCTCCATCCTCGCCACCGACTACCCTGTCGATAAGTACGCCTGCTACCTCTCAGACGACGGCGGCACGTTGGTGCACTACGAGGCCATGATCGAAGTGGCCAATTTCGCGGTGATGTGGGTCCCTTTTTGCCGGAAGCACTGTGTCGAGCCAAGGTCCCCCGAGAACTACTTTGGGATGAAAACGCAGCCGTACGTCGGGAGTATGGCTGGAGAATTCATGAGGGAGCATAGGCGTGTGCGCAGAGAGTATGATGAGTTCAAGGTGAGGATAGACTCCCTGTCCACCACCATCCGCCAAAGATCTGATGCGTACAACTCGAGCAACAAAGGAGATGGTGTGCGTGCAACCTGGATGGCTGATGGGACACAATGGCCTGGTACGTGGATTGAGCAGGTTGAGAACCACCGGAGAGGACAACATGCTGGAATTGTTCAGGTCATACTAAGCCATCCTAGTTGCAAACCGCAACTGGGGTCTCCGGCGAGCACTGACAATCCACTTGACTTCAGCAACGTTGACACGAGGCTGCCCATGCTCGTCTACATGTCCCGGGAGAAGCGCCCCGGTTATAACCACCAAAAGAAGGCAGGCGCCATGAACGTGATGCTCCGTGTCTCGGCGTTGCTCTCCAACGCGCCATTCGTCGTCAATTTTGACTGCGACCACTACATCAACAACACGCAAGCTCTCCGCGCCCCTATGTGCTTCATGCTCGACCCTCGCGACGGTCAGAACACGGCCTTCGTCCAGTTTCCGCAGCGCTTCGACGACGTCGACCCGACGGACCGCTACGCCAACCACAACCGTGTCTTCTTCGACGGTACCATGCTCTCCCTCAACGGCCTTCAAGGGCCTTCCTACCTCGGCACTGGCACCATGTTCCGTCGTGTCACGCTCTATGGCATGGAGCCACCACGTTATAGAGCGGAGAACATCAAGCTTGTAGGTAAGACCTATGAGTTCGGTAGCTCGACGTCTTTCATCAATTCCATGCCGGACGGCGCAATCCAAGAGCGGTCTATCACGCCGGTGTTGGTCGACGAGGCACTCAGCAATGACCTGGCTACCCTGATGACGTGTGCTTACGAGGACGGGACCTCATGGGGGAGAGACGTTGGGTGGGTGTACAACATCGCGACGGAGGACGTGGTGACCGGATTCCGCATGCACCGGCAGGGGTGGCGCTCCATGTATTGCTCCATGGAGCCGGCCGCCTTCCGCGGAACAGCGCCGATCAACCTCACCGAGCGCCTTTACCAGGTGCTCCGGTGGTCGGGCGGCTCTCTCGAGATGTTCTTCTCCCACAGCAACGCTCTCATGGCCGGCCGCCGTATCCACCCTCTGCAGCGTGTCGCGTACCTCAACATGTCGACCTACCCGATCGTCACGGTGTTCATCCTGGCCTACAACCTCTTCCCCGTCATGTGGCTCTTCTCCGAGCAGTTCTACATCCAGAGGCCGTTCGGCACGTACATCATGTACCTCGTCGGCGTCATAGCGATGATTCACGTGATCGGCATGTTCGAGGTGAAATGGGCGGGGATCACGCTGCTCGACTGGTGCCGCAACGAGCAGTTCTACATGATCGGGGCGACGGGCGTGTACCCGACGGCGGTGCTTTACATGGCGCTCAAGCTTGTCACGGGGAAGGGGATATACTTCAGGCTCACATCCAAGCAGACGGACGCTTGCTCCAACGACAAGTTCGCCGACCTGTACACGGTGCGGTGGGTGCCGCTGCTGTTCCCGACGGTCGCAGTGCTCATCGTGAACGTCGCGGCTGTCGGGGCAGCGATAGGCAAGGCAGCAGCGTGGGGCTTCTTCACGGACCAGGCGCGGCACGTGCTGCTCGGGATGGTGTTCAACGTGTGGATCCTCGTGCTCCTCTACCCGTTTGCGCTCGGGATCATGGGGAAATGGGGGAAGAGACCCATCATCCTGTTCGTCATGTTGATCATGGCCATTGGCGCCGTCGGGCTCGTGTATGTCGCCTTCCATGATCCCTACCCAACTGATTTTTCAGAAGTTGCAGCTTCTCTTGGTGAAGCATCGCTGACCGGGCCATCTGGG

>HvCSLF6

ATGGCGCCAGCGGTGGCCGGAGGGGGCCGCGTGCGGAGCAATGAGCCGGTTGCTGCTGCTGCCGCCGCGCCGGCGGCCAGCGGCAAGCCCTGCGTGTGCGGCTTCCAGGTTTGCGCCTGCACGGGGTCGGCCGCGGTGGCCTCCGCCGCCTCGTCGCTGGACATGGACATCGTGGCCATGGGGCAGATCGGCGCCGTCAACGACGAGAGCTGGGTGGGCGTGGAGCTCGGCGAAGATGGCGAGACCGACGAAAGCGGTGCCGCCGTTGACGACCGCCCCGTATTCCGCACCGAGAAGATCAAGGGTGTCCTCCTCCACCCCTACCGGGTGCTGATTTTCGTTCGTCTGATCGCCTTCACGCTGTTCGTGATCTGGCGTATCTCCCACAAGAACCCAGACGCGATGTGGCTGTGGGTGACATCCATCTGCGGCGAGTTCTGGTTCGGTTTCTCGTGGCTGCTGGATCAGCTGCCCAAGCTGAACCCCATCAACCGCGTGCCGGACCTGGCGGTGCTGCGGCAGCGCTTCGACCGCCCCGACGGCACCTCCACGCTCCCGGGGCTGGACATCTTCGTCACCACGGCCGACCCCATCAAGGAGCCCATCCTCTCCACCGCCAACTCGGTGCTCTCCATCCTGGCCGCCGACTACCCCGTGGACCGCAACACATGCTACGTCTCCGACGACAGTGGCATGCTGCTCACCTACGAGGCCCTGGCAGAGTCCTCCAAGTTCGCCACGCTCTGGGTGCCCTTCTGCCGCAAGCACGGGATCGAGCCCAGGGGTCCGGAGAGCTACTTCGAGCTCAAGTCACACCCTTACATGGGGAGAGCCCAGGACGAGTTCGTCAACGACCGCCGCCGCGTTCGCAAGGAGTACGACGAGTTCAAGGCCAGGATCAACAGCCTGGAGCATGACATCAAGCAGCGCAACGACGGGTACAACGCCGCCATTGCCCACAGCCAAGGCGTGCCCCGGCCCACCTGGATGGCGGACGGCACCCAGTGGGAGGGCACATGGGTCGACGCCTCCGAGAACCACCGCAGGGGCGACCACGCCGGCATCGTACTGGTGCTGCTGAACCACCCGAGCCACCGCCGGCAGACGGGCCCGCCGGCGAGCGCTGACAACCCACTGGACTTGAGCGGCGTGGATGTGCGTCTCCCCATGCTGGTGTACGTGTCCCGTGAGAAGCGCCCCGGGCACGACCACCAGAAGAAGGCCGGTGCCATGAACGCGCTTACCCGCGCCTCGGCGCTGCTCTCCAACTCCCCCTTCATCCTCAACCTCGACTGCGATCATTACATCAACAACTCCCAGGCCCTTCGCGCCGGCATCTGCTTCATGGTGGGACGGGACAGCGACACGGTTGCCTTCGTCCAGTTCCCGCAGCGCTTCGAGGGCGTCGACCCCACCGACCTCTACGCCAACCACAACCGCATCTTCTTCGACGGCACCCTCCGTGCCCTGGACGGCATGCAGGGCCCCATCTACGTCGGCACTGGGTGTCTCTTCCGCCGCATCACCGTCTACGGCTTCGACCCGCCGAGGATCAACGTCGGCGGTCCCTGCTTCCCCAGGCTCGCCGGGCTCTTCGCCAAGACCAAGTACGAGAAGCCCGGGCTCGAGATGACCACGGCCAAGGCCAAGGCCGCGCCCGTGCCCGCCAAGGGTAAGCACGGCTTCTTGCCACTGCCCAAGAAGACGTACGGCAAGTCGGACGCCTTCGTGGACACCATCCCGCGCGCGTCGCACCCGTCGCCCTACGCCGCGGCGGCTGAGGGGATCGTGGCCGACGAGGCGACCATCGTCGAGGCGGTGAACGTGACGGCCGCCGCGTTCGAGAAGAAGACCGGCTGGGGCAAAGAGATCGGCTGGGTGTACGACACCGTCACGGAGGACGTGGTCACCGGCTACCGGATGCATATCAAGGGGTGGCGGTCACGCTACTGCTCCATCTACCCACACGCCTTCATCGGCACCGCCCCCATCAACCTCACGGAGAGGCTCTTCCAGGTGCTCCGCTGGTCCACGGGATCCCTCGAGATCTTCTTCTCCAAGAACAACCCGCTCTTCGGCAGCACATACCTCCACCCGCTGCAGCGCGTCGCCTACATCAACATCACCACTTACCCCTTCACCGCCATCTTCCTCATCTTCTACACCACCGTGCCGGCGCTATCCTTCGTCACCGGCCACTTCATCGTGCAGCGCCCGACCACCATGTTCTACGTCTACCTGGGCATCGTGCTATCCACGCTGCTCGTCATCGCCGTGCTGGAGGTCAAGTGGGCCGGGGTCACAGTCTTCGAGTGGTTCAGGAACGGCCAGTTCTGGATGACAGCAAGTTGCTCCGCCTACCTCGCCGCCGTCTGCCAGGTGCTGACCAAGGTGATATTCCGGCGGGACATCTCCTTCAAGCTCACATCCAAGCTACCCTCGGGAGACGAGAAGAAGGACCCCTACGCCGACCTCTACGTGGTGCGCTGGACGCCGCTCATGATTACACCCATCATCATCATCTTCGTCAACATCATCGGATCCGCCGTGGCCTTCGCCAAGGTTCTCGACGGCGAGTGGACGCACTGGCTCAAGGTCGCCGGCGGCGTCTTCTTCAACTTCTGGGTGCTCTTCCACCTCTACCCCTTCGCCAAGGGCATCCTGGGGAAGCACGGAAAGACGCCAGTCGTGGTGCTCGTCTGGTGGGCATTCACCTTCGTCATCACCGCCGTGCTCTACATCAACATCCCCCACATGCATACCTCGGGAGGCAAGCACACAACGGTGCATGGTCACCATGGCAAGAAGTTGGTCGACACAGGGCTCTATGGCTGGCTCCAT

>HvCSLF7

ATGTTAATGACATATATCACCAAGAAGCATGACTATGTTGCAACCTTGGATGAGAAGGAGTCGCCCGCGGATGAGAAGTCGGCCAATGTCGAGAGGTTGCTTGTCCGGACCACAAAACTTACAACAGTTACCATCAAGTTGTACAGGCTCATGATCGTTGTTCGGATGGCCATCTTCGTGCTGTTCTTCAAATGGCGAATCGGCACTGCTCTTGCGATGACCAGCAACGGCACAAGTACAGCTCGTGCAATGTGGACGGTGTCCATCGCCGGGGAGCTCTGGTTCGCCCTAATGTGGGTGCTGGACCAGCTGCCCAAGATGCAGCCTGTCCGGCGCACCGTCTTCGCCACCGCGCTGGAGGAGTCGCTGCTTCCGACCATGGATGTGTTCGTCACCACCGCCGACCCCGACAAGGAGCCGCCGCTGGTGACCGTGAACACTATCCTCTCCATCCTTGCCGCCGACTATCCCCCAGACAAGCTCACATGCTACGTCTCAGACGACGGCGGCGCTCTGCTCACGCCCGAGGCTGTGGTGGAGGCTGCCCGGTTCGCCGGACTGTGGGTGCCGTTCTGCCGGAAGCACGGGGTTGAGCCGAGGAACCCAGAGGCCTACTTCAGCCACGGCGTTAAGGTGAGGGTGGTGTCAAGGGCTGACTATAAGGGAAGATCGTGGCCGGAACTGGCACGGGACAGAAGGCGTGTGCGCCGGGAGTACGAAGAACTGCGGCTGCGGGTCGACGCGCTTCACGCCGGAGACGTGCAGCGCCCGTGGCGGTCGCGCGGCACGCCGGAAGATCATGCCGGAGTTGTTGAGGTGCTAGTGGATCCTCCCAGCTGTACGCCAGAGCCCGGCGTCAGTGGTAATCTACTGGACCTCAGCTCCGTCGATGTGCGGGTTCCAGCGCTCGTGTACATGTGCCGGGAGAAGCGCCGCGGCCGCGCGCACCACCGGAAGGCAGGTGCCATGAACGCGCTGCTCCGGACCTCGGCCGTGCTCTCCAACGCGCCCTTCATCCTCAACCTCGACTGCGACCACTACGTCAACAACTCGCAGGCCCTCCGCGCGGGCGTCTGCCTCATGCTCGACCGCGGCGGCAGCGACGTGGCGTTTGTCCAGTTCCCGCAGCGCTTCGACGGCGTCGACCCCGCCGACCGGTACGCCAACCACAACCGCGTCTTCTTCGACTGCACGGAGCTCGGCCTCGACGGCCTCCAGGGACCCATTTACGTGGGCACCGGGTGCATGTTTCGCCGTGCGGCGCTATACAGCGTCGACCCGCCGCTCTGGTGGTCACATGGCGACAGCGACGCCGGCAAGGACGTCGCTGCAGAGGCCGACAAGTTTGGCGTTTCGACGCCGTTCCTTGGCTCGGTGCGTGCGGCCTTGAACTTGAACCGGTCGGAGCAACGGAACACAGGTACTTCACCGCCGTGCTCGTTCGACGCGGCTGCCGTCGGCGAGGCCACCGCGCTCGTCTCGTGCGGCTACGAAGACAGGACGGCATGGGGCAGGGAAATCGGCTGGATATACGGGACGGTGACAGAGGACGTGGCCACGGGCTTCTGCATGCACCGGCGAGGGTGGCGCTCCGCCTACTGCGCCACCGCGCCGGACGCGTTCCGCGGCACGGCGCCCATCAACCTCACAGACCGGCTGCACCAGGTCCTCCGCTGGGCGGCGGGCTCCCTCGAGATATTCTTCTCCCGCAACAACGCCCTCTTCGCCGGGCCCCGGCTCCACCCGCTGCAGCGGCTGGCGTACCTCAACACGACGGTGTACCCGTTCACCTCCATCTTCCTCCTGGTCTACTGCCTCTTGCCGGCGATCCCGCTCGTGACCCGGAGCGCCTTCCCAGTCAGCATGCCGCCGTCGTCCACGTACATGGCCTTTGTGGCGGCACTGATGCTGACGCTGGCCATGGTGGCCGTGCTGGAGGTGAGGTGGTCGGGCATAACGCTGGGCGAGTGGTGGCGGAACGAGCAGTTCTGGATGGTTTCCGCCACGAGCGCGTACGCGGCCGCGGTGGTGCAGGTGGCGCTCAAGGTTTTGGCGGGGAAGGAGATAGCGTTCAAGCTGACGTCCAAGCGGCGCGCGTCGGGCTCCGGCGGCGGCGGCGTAGTAAAAGGCAGGTTCGCGGAGCTGTACGCCGTGAGATGGACGGTGCTAATGGTTCCGACGGCGGTGGTGCTGGCGGTGAACGTGGCGTCCATGGCAGCGGCAGTACAGGAGAGGCGGTGGAGGAAGGGCCCCGCGGCGGTGCTCGCGACGGCGTTCAACGCTTGGGTGGTGGTGCATCTCCACCCCTTCGCCCTTGGGCTCATGGGCCGTTGGAGCAAGACGTTGAGCCCCCTGCTCTTGCTTGTCGTAGCGTTCACAATTCTATCACTATGTTTTCTCCTCCATTTGCATATGTTT

>HvCSLF8

ATGGGTTCTTTGGCGGCAGCCAACGGGGCCGGTCATGCGAGCAATGGCGCCGGCGTCGCGGACCAGGCGCTGGCACTGGAGAACGGCACCGGCAATGGGCACAAGGCCAGCGACGCCAACCGAGCGACGCCGGTACAGCAGGCAAACGGCAGCAGCAAGGCCGCGGGGAAGGTTAGCCCGAAGGACAAGTACTGGGTGGCCGTCGATGAGGGAGAGATGGCGGCCGCCATAGCGGACGGCGGCGAGGACGGCCGGCGACCGCTGCTGTACCGGACGTTCAAGGTCAAGGGCATCCTCCTGCATCCCTACAGGTTGCTGAGCTTGATCAGATTGGTTGCTATCGTCCTATTTTTCGTATGGCGTGTCAGGCACCCATACGCTGATGGCATGTGGCTCTGGTGGATATCGATGGTTGGGGATCTTTGGTTTGGCGTCACTTGGTTGCTAAACCAAGTTGCAAAGCTCAACCCTGTCAAGCGTGTCCCCAACCTTGCGCTTTTGCAACAGCAGTTTGATCTCCCTGACGGCAACTCCAACCTTCCTTGTCTTGATGTCTTCATCAACACCGTTGATCCCATTAATGAACCTATGATATACACTATGAACTCCATCATATCCATCCTTGCTGCAGACTATCCGGTTGACAAGCATGCTTGCTACCTTTCAGATGATGGTGGGTCAATAATCCATTATGATGGTTTGCTTGAGACTGCAAAATTTGCTGCATTATGGGTTCCCTTTTGCAGAAAACATTCCATTGAGCCAAGAGCCCCTGAGAGCTATTTTTCTTTGAATACACGCCCATACACTGGAAATGCACCACAAGACTTTGTCAATGACCGCAGACACATGTGTAGAGAGTATGATGAGTTCAAGGAGCGCTTAGATGCACTTTTTACCCTCATTCCCAAACGGTCAGATGTGTACAATCATGCTGCTGGCAAAGAAGGTGCAAAGGCAACTTGGATGGCAGATGGGACACAGTGGCCAGGCACATGGATTGACCCAGCTGAAAACCATAAGAAAGGACAACATGCTGGGATCGTTAAGGTTTTGTTGAAACATCCAAGTTATGAACCAGAACTTGGTCTAGGAGCAAGCACCAACAGTCCTCTAGACTTCAGTGCAGTTGATGTGCGCCTCCCAATGCTCGTTTACATCTCCCGTGAGAAGAGTCCAAGCTGTGATCATCAAAAGAAGGCAGGTGCCATGAACGTACAGTTGCGAGTCTCTGCCCTCCTGACCAATGCGCCCTTTATCATCAACTTTGATGGTGACCACTACGTCAACAACTCGAAAGCCTTCCGTGCTGGCATATGTTTCATGCTCGATCGCCGTGAAGGTGACAATACTGCCTTTGTCCAGTTTCCCCAACGCTTCGATGATGTTGATCCCACAGATAGGTACTGCAATCACAATCGTGTCTTCTTTGACGCCACCTTGCTCGGCCTCAATGGCATCCAAGGGCCGTCTTATGTTGGCACTGGTTGCATGTTCCGCCGTGTCGCACTTTACGGTGTTGACCCACCTCGCTGGAGACCTGATGACGTGAAGATCGTGGACAGCTCCAGCAAGTTTGGCAGTTCAGAGTCATTCATCAGCTCAATACTGCCAGCAGCAGACCAAGAACGCTCCATCATGTCGCCACCGGCACTTGAAGAGTCTGTCATGGCTGACTTAGCTCATGTCATGACTTGTGCATATGAGGACGGGACTGAATGGGGCAGAGAAGTTGGTTGGGTGTACAACATTGCAACTGAGGATGTGGTGACCGGCTTCCGGCTGCACCGGAATGGGTGGCGATCCATGTACTGCCGCATGGAACCAGATGCATTCGCCGGCACCGCACCAATCAACCTCACTGAGCGGCTCTACCAGATCCTGCGCTGGTCGGGGGGCTCCCTTGAGATGTTCTTCTCGCACAACTGCCCACTCCTGGCTGGCCGCCGCCTCCACCCAATGCAAAGAATTGCCTATGCCAACATGACAGCCTACCCAGTTTCATCTGTCTTTCTTGTGTTCTATCTCCTCTTCCCGGTGATATGGATCTTCCGTGGGCAATTCTACATACAGAAGCCATTCCCCACGTATGTGTTGTACCTCGTCATCGTCATAGCCCTGACCGAGTTAATCGGTATGGTTGAGATCAAGTGGGCTGGGCTCACGCTGCTGGACTGGATCCGCAACGAGCAGTTCTACATTATTGGTGCAACAGCCGTGTACCCTACAGCAGTATTTCACATAGTGCTGAAGCTGTTTGGCCTGAAGGGTGTTTCATTCAAGCTGACGGCAAAACAGGTAGCAAGCAGTACCAGCGATAAGTTTGCTGAACTGTATGCCGTGCAGTGGGCTCCGATGCTGATCCCTACCATGGTGGTTATAGCGGTGAATGTTTGTGCCATTGGCGCATCGATAGGCAAGGCGGTAGTGGGAGGATGGTCACTGATGCAGATGGCCGATGCAGGACTTGGGCTGGTGTTCAACGCGTGGATTCTGGTGCTGATCTACCCGTTTGCACTGGGCATGATTGGACGGTGGAGCAAGAGGCCCTACATCCTGTTCATTCTGTTTGTCATTGCGTTTATTTTGATCGCATTGGTGGATATCGCCATCCAGGCCATGCGGTCTGGGATTGTTCGGTTCCACTTTAAAAGCTCAGGTGGCGCCACTTTTCCCACAAGCTGGGGTTTG

>HvCSLF9

ATGGCTTCTCCGGCGGCCGTCGGCGGGGGTCGTCTAGCCGACCCACTGCTGGCCGCCGACGTCGTCGTCGTCGGCGCCAAAGACAAGTACTGGGTGCCCGCCGACGAGAGAGAGATCCTGGCGTCGCAGAGCAGCGGCGGCGGTGAACAGGACGGCCGGGCACCGCTGCTATACCGCACGTTCAGGGTCAAGGGCTTCTTCATCAACCTTTACAGGTTATTGACTCTGGTCAGAGTTATCGTGGTTATTCTATTCTTCACGTGGCGCATGAGGCACCGGGACTCGGACGCGATGTGGCTGTGGTGGATCTCGGTCGTGGGCGACCTCTGGTTCGGAGTCACCTGGCTGCTCAACCAGATCACCAAGCTCAAGCCCAGGAAATGCGTCCCCAGCATCTCCGTCCTGAGAGAGCAGCTCGACCAGCCCGACGGCGGCTCCGACCTGCCCCTTCTCGACGTGTTCATCAACACCGTCGACCCGGTGGACGAGCCGATGCTCTACACCATGAACTCCATCCTCTCCATCCTGGCCACCGACTACCCCGTCCAGAAGTACGCCACCTATTTCTCCGATGACGGCGGGTCGCTGGTGCACTACGAGGGGCTGCTGCTGACGGCGGAGTTCGCCGCGTCGTGGGTCCCGTTCTGCCGGAAGCATTGCGTCGAGCCTCGCGCCCCGGAGAGCTACTTCTGGGCCAAGATGCGCGGGGAGTACGCCGGCAGCGCGGCCAAGGAGTTCCTTGACGACCATCGGAGGATGCGCGCGGCGTATGAGGAGTTCAAGGCGAGGCTGGACGGGCTTTCTGCCGTCATCGAGCAGCGGTCCGAGGCGTGCAACCGCGCTGCAAACGAGAAAGAAGGGTGTGGGAACGCGACTTGGATGGCCGATGGGTCGACGCAATGGCAGGGGACGTGGATCAAGCCGGCCAAGGGCCACCGGAAAGGACACCATCCTGCAATTCTTCAGGTTATGCTGGATCAACCTAGCAAGGATCCTGAGCTGGGAATGGCGGCGAGCTCCGACCACCCTCTGGATTTCAGCGCCGTGGACGTGCGCCTCCCGATGCTGGTCTACATTGCCCGGGAGAAGCGGCCTGGGTATGACCACCAGAAGAAGGCGGGCGCCATGAACGTGCAGCTGCGCGTGTCCGCGCTGCTCTCCAACGCGCCCTTCATCATCAACTTCGACGGCGACCACTACATCAACCACTCGCAGGCCTTCCGCGCCGCCATGTGCTTCATGCTCGACCCGCGCGACGGCGCCGACACCGCCTTCGTCCAGTTCCCGCAGCGCTTCGACGACGTCGACCCCACCGACCGCTACTGCAACCACAACCGCATGTTCTTCGACGCCACCCTCCTCGGCCTCAACGGCATCCAGGGCCCCTCCTTCGTCGGCACCGGATGCATGTTCCGCCGCGTCGCTCTCTACAGCGCCGACCCTCCACGGTGGCGGTCCGACGACGCCAAGGAGGCCAAGGCCTCGCACAGGCCCAACATGTTTGGCAAGTCTACGTCCTTCATCAACTCAATGCCGGCGGCCGCCAACCAAGAACGGTCCGTCCCGTCACCGGCGACAGTCGGCGAGGCGGAGCTCGCAGACGCGATGACTTGCGCGTACGAGGACGGCACCGAGTGGGGCAACGACGTTGGGTGGGTGTACAACATCGCGACGGAGGACGTGGTGACCGGCTTCCGGCTGCACCGGACGGGGTGGCGCTCCACGTACTGCGCCATGGAGCCCGACGCGTTCCGCGGCACGGCGCCCATCAACCTCACCGAGCGCCTGTACCAGATCCTGCGTTGGTCGGGGGGATCCCTCGAGATGTTCTTCTCCCGCTTCTGCCCGCTCCTGGCCGGCCGCCGCCTCCACCCCATGCAGCGCGTCGCCTACATCAACATGACCACCTACCCGGTCTCCACCTTCTTCATCCTCATGTATTACTTCTACCCGGTCATGTGGCTCTTCCAGGGGGAGTTCTACATCCAGAGGCCGTTCCAGACGTTCGCGCTCTTCGTCGTCGTCGTCATCGCCACGGTGGAGCTCATCGGCATGGTGGAGATCAGGTGGGCAGGCCTCACGCTGCTCGACTGGGTCCGCAACGAGCAGTTCTACATCATCGGCACCACCGGCGTGTACCCGATGGCCATGCTGCACATCCTCCTCAGGTCCCTCGGCATAAAGGGGGTGTCCTTCAAGCTGACGGCCAAGAAGCTCACGGGGGGCGCCAGGGAGAGGCTCGCGGAGCTGTACGACGTGCAGTGGGTGCCGTTGCTGGTGCCCACCGTGGTGGTCATGGCCGTGAACGTGGCCGCCATCGGCGCGGCGGCGGGCAAGGCGATCGTTGGGCGGTGGTCGGCAGCGCAGGTCGCGGGGGCGGCGAGCGGGCTTGTTTTCAACGTGTGGATGCTGCTGCTGCTCTACCCGTTCGCGCTCGGGATAATGGGGCACTGGAGCAAGAGGCCCTACATCCTGTTCCTTGTGCTGGTGACCGCGGTCGCTGCCACCGCGTCCGTGTACGTCGCACTCGCGGGGTCCTTGCTGTACTTGCATTCGGGGATAAAACTAGTT

>HvCSLF10

ATGTTGTCGCCCCGGACAGACGCCGGCGCCGGCGCCGCCACCGACCTCAGCCAGCCACTTCTCTGGAACCGCAACGGCGTTCACGCAGGAGCATTGGTCGTCATGCCAGTCGTGGCCAATGGTCACGGCGGCGGCGACAAGCTTAAGGGCGCCCCGAAAGCCAAGGACAAGTACTGGAAAGACGTCGACCAGCCGGACGACATGGCGGCAGCGCCAGACCTGGAGAATGGCGGCGGCCGGCCGCTGCTGTTCTCGAACAGGAGAGTCAAGAATATCATCCTGTACCCCTACAGGGTATTGATCCTGATACGAGTAATCGCCGTAATCTTATTTGTTGGATGGCGCATCAAGCATAACAATTCAGATGTCATGTGGTTTTGGATGATGTCCGTCGTCGCAGACGTGTGGTTTAGCTTATCATGGCTAAGCTACCAACTGCCAAAGTATAATCCCGTTAAAAGGATACCCGACCTTGCTACACTCAGGAAACAATATGACACACCAGGGAGGAGCTCCCAGCTGCCAAGCATTGACGTCATCGTCACCACTGCCAGTGCTACCGATGAGCCCATATTGTACACCATGAACTGTGTTCTCTCTATACTTGCAGCTGACTATCATATTGGCAGGTGCAACTGCTACCTATCAGATGATAGCGGCTCATTGGTCCTTTATGAGGCATTGGTTGAGACTGCAAAGTTTGCTGCTTTATGGGTTCCTTTCTGTAGAAAGCATCAGATTGAGCCAAGAGCACCGGAAAGCTATTTTGAACTAAAGGGCCCGTTGTATGGAGGGACGCCACATAAGGAGTTCTTTCAGGATTATAAGCATGTACGCACACAATATGAAGAGTTCAAGAAGAATTTAGATATGCTTCCTAACACCATCCATCAAAGGTCGGGAACTTACAGTAAAACAGGAACGGAGGATGAAGATGCAAAAGTGACTTGGATGGCTGACGGAACACAATGGCCAGGCACATGGCTTGACCCAGCAGAAAAACATAGGGCCGGGCATCATGCAGGAATTGTTAAGATTGTGCAGAGCCATCCAGAACATGTGGTTCAACCAGGCGTACAAGAGAGCCTTGACAACCCACTCAGCTTTGACGATGTTGATGTGCGCCTGCCCATGTTGGTATATGTGGCTCGTGAAAAGAGTCCAGGTATCGAGCATAACAAAAAGGCAGGCGCTTTGAATGCAGAGCTACGTATCTCAGCTCTACTCTCTAATGCACCTTTCTTCATTAACTTTGACTGCGACCACTACATCAACAATTCAGAAGCCCTACGTGCAGCTGTTTGCTTCATGCTAGACCCACGTGAAGGGGATAATACTGGATTTGTTCAGTTCCCGCAAAGATTTGATAATGTCGACCCAACTGACCGGTATGGAAACCATAATCGAGTCTTTTTTGATGGTGCCATGTATGGCCTCAATGGTCAACAAGGGCCTACTTACCTTGGCACAGGTTGCATGTTCCGTCGCCTTGCACTCTATGGAATTGATCCACCTTGCTGGAGAGCCGAGGACATCATAGTCGACAGTAACAGGTTTGGCAACTCATTACCCTTCCTCAACTCAGTACTAGCAGCCATAAAGCAAGAGGAAGGTGTCACACTACCACCACCACTAGATGATTCATTTCTTGAAGAGATGACAAAAGTTGTGTCATGTTCCTATGATGATTCCACTGATTGGGGTAGGGGCATTGGCTACATATACAATATGGCAACAGAAGACATAGTAACAGGATTTCGTATCCATGGGCAAGGGTGGTGCTCCATGTATGTTACCATGGAACGTGAAGCGTTCCGTGGCACTGCACCGATCAATCTAACAGAGCGCCTCCGCCAAATAGTGCGATGGTCTGGTGGTTCCCTAGAGATGTTCTTCTCGCACATCAGCCCACTATTCGCTGGTCGTCGACTCAGTTTGGTGCAGCGACTCTCGTACATCAATTTCACTATATACCCATTGACATCACTCTTTATCCTAATGTATGCCTTCTGTCCAGTGATGTGGCTTCTTCCAACAGAAATACTTATACAAAGGCCATATACCAGGTACATTGTGTACCTTATCATTGTCGTCGCGATGATCCATGTGATTGGCATGTTTGAGATAATGTGGGCAGGAATCACATGGTTGGATTGGTGGCGCAACGAGCAATTTTTCATGATCGGCTCGGTAACTGCATATCCAACGGCGGTGTTGCACATGGTGGTGAATATCCTTACAAAAAAGGGTATACACTTCAGAGTAACCACAAAGCAACCAGTGGCTGATACAGATGACAAGTATGCTGAGATGTATGAAGTGCATTGGGTACCCATGATGGTCCCCGCGGTTGTGGTATTGTTTTCCAACATCTTGGCTATTGGTGTAGCAATTGGTAAATCAGTCTTATACATGGGGACATGGTCTGTAGCACAGAAAAGGCATGGTGCACTAGGGCTATTGTTCAACCTGTGGATTATGGTGCTCCTTTACCCATTTGCATTGGCGATTATTGGAAGATGGGCCAAGAGAACCGGAATCCTATTCATCTTACTACCCATTGCTTTCTTGGCCACCGCATTGATGTACATTGGCATCCATACATTCCTTTTACATTTCTTTCCATCCATGTTGGTA

>HvCSLF11

ATGGCCCTGCGAGTGGAAGCCGACGCCGACCCCAAGGGCAGGCGCGCCACGGACACCGACGCCGACGACGTGTGGGTGGCGGCGGAGGAGGGAGACATGTCTGGAGCCAGCGCCGGCCGGCCGCTGCTGTTCCGGACCATGAAGGTCAAGGGAAGCATCCTGCATCCTTACAGGTTCTTGATTCTGGTGCGTTTGGTCGCCATCGTCGCCTTCTTCGCGTGGCGCGTAGAGCACAGGAACCACGATGGCATGTGGCTCTGGGCCACATCCATGGTGGCGGACGCCTGGTTCGGTTTCTCATGGCTCCTCAACCAGCTCCCCAAGCTCAACCCCACCAAACGAGTGCCGGACCTTGCCGCCCTGGCGGACCGGCACGACGATGCCATCCTTCCTGGTATCGACGTCTTCGTCACCACTGTCGACCCCGTGGATGAACCCGTCTTGTACACCGTGAACACCATCCTCTCTATCCTTGCCGCCGATTACCCCGTCGACAAGTATGCCTGCTACCTCTCCGACGATGGTGGCACATTGGTGCACTATGAGGCAATGCTACAGGTTGCCAGTTTTGCCGCTTTGTGGGTCCCGTTTTGCCGGAAGCATTGCATCGAGCCAAGGTCCCCGGAGAACTATTTTGGGATGAAAACCAGGCCATACGTCGGTGGTATGGCAGGAGAGTTCATGAGTGATCACAGGCGTGTGCGCAGAGAATATGGCGAGTTCAAGGTGAGAATAGAGTCCCTTTCTACGACCATCCGCCGACGATCTGATGCCTACAACAAAGGAGATGATGGTGTGCACGCCACTTGGATGGCTGATGGGACACCATGGCCGGGCACATGGATTGAGCAAGCTGACAACCATGGAAGAGGACAACATGCTGGAATTGTTGAGGTCATGCTAGACCATCCAAGTTGTAAACCGCAGCTTGGATTCTCGGCGAGCACCGACAATCCAATTGACTTGAGCAACGTTGACACAAGACTCCCCATGCTCGTCTACATTTCCCGGGAGAAGCGCTCCGGTTATGACAACCAAAAGAAGGCAGGCGCCATGAACGCGATGCTCCGCGTCTCCGCGTTACTCTCCAATGCGCCCTTCGTCATCAACTTTGACTGCGACCACTACATCAACAACTCGCGAGCTCTCCGCGCCCCTATGTGCTTCATGCTCGACCCTCGCGACGGTCAGAACACGGCCTTCGTCCAGTTCCCGCAACGCTTCGATGATGTCGATCCGACAGATCGCTACTCCAACCACAACCGTGTCTTCTTCGATGGCACCATGCTTTCGCTCAACGGTCTCCAAGGGCCCACCTACCTCGGCACCGGCACCATGTTCCGTCGTGTCGCCCTCTATGGCATGGAGCCACCACGCTATAGAGCGGAGGACATCAAGCTGGTAGGTAAGGCCGTTGAGTTGGGTAACTCGACGCCATTCCTGAACTCAATACCGGATGGCGCAATCCAAGAGCGATCAATCACCCCTGTGTTGGTCGACGACGAGCTCAACAATGACCTGGCCACCCTGATGGCATGTGGTTACGAGGACGGGAGCTCATGGGGGAGAGACGTCGGGTGGGTGTACAACATCGCGACGGAGGACGTGGTGACCGGATTCCGCATCCACCGACAGGGGTGGCGTTCCATGTATTGCTCCATGGAGCCGGCCGCGTTCCGCGGAACGGCTCCGATCAACCTCACCGAGCGCCTCTACCAGGTGCTCCGGTGGTCGGGCGGCTCCCTCGAGGCCTTCTTCTCCCACAGCAACGCTCTCATCGCCAGCCGTCGGCTCCACCTTCTGCAGCGCATCGCTTACCTCAACATGTCCATCTACCCGATCGCCACCATGTTCATCCTGGCATACAGCTTCTTCCCGGTGATGTGGCTCTTCTCCGAGCAGTCGTACTACATCCAGAGACCGTTCGGCACCTTCATCATGTACCTCGTCGCTGTCATAGCGATGATGCACGTGATCGGCATGTTCGAGGTGAAATGGTCGGGGATCACGCTGCTGGACTGGTGGCGCAACGAGCAGTTCTACATGATCGCGGCCACGGGCGTGTACCCGACGGCGGTGTTGTACATGGCGCTGAAGCTCGTGAGGGGGAAGGGGATACACTTCAGGCTCACGTCCAAGCAGACGGAGGCCTGCTCCGGCGAGAAGTTCGCCGACCTGTACGCCGTGCGGTGGGTGCCGTTGCTGATCCCGACCGTGGCGGTGCTCGTCGTGAACGTCGCGGCCGTCGGGGCGGCGATAGGCAAGGCAGCGACATGGGGGTTCTTCACGGACCAGGCGTGGCACGCGGTGCTCGGGATGGTGTTCAACGTGGGTACCCTGGTGCTCCTCTACCCGTTTGCACTCGGCATCATGGGGCAATGGGGGAAGAGACCAGGCATCCTGTTGGTCATGTTGGTCATGGCCATTGCCACCGTCGGGCTCTTGTATGTCGCACTCCAGCAGGATGGTCACAGCATGTCGTTCCTGACCAGGCCATCTGGA

>HvCSLF12

ATGTGGCTGTGGTGGATCTCGGTCGTCGGAGATTTCTGGTTCGGCCTGAGCTGGTGGCTAAACCAGGTCCCGAAGCTCAACCCGACCATATGCATCCCCACCATCTCCCTCCTGAGACAGCAGTTCGACCTGCCCGACGGCGGCTCCAACCTCCCCATCCTGGATGTGTTCATCAGCACCGTCGACCCCGTGGAGGAGCCCATGCTCCACACAATGAACTCCATCCTCTCCATCCTCGCCACCGACTACCCGGTCGACAAGTACGCCACCTACCTCTCGGACGACGGCGGATCACTGCTCCACTACGACGGGCTGGTCGAGACCGCCAAGTTCGCCGCGCTGTGGGTTCCCTTCTGCCGGAAGCATGGCGTTGAACCAAGGGCGCCGGAGAGCTACTTCGGGATGAAGATCCGCCCGTACACGGGGAATATGCCCGAAGAGTTCCTCGATGACCATAGGCGTCTGCGGAGGGAGTATGAGGAGTTCAGGACGCGCTTGGACGCACTGTTCACTGTCATCCCGCAACGGTCAGAGGCGCACGGCCGCCAAGACGCCAAACAAGGAGGAGGAGCCAAGGCGACTTGGATGGCGGACGGGACACAGTGGCCTGGCACATGGACTGAGCCGGCGGATGGTCACCGGAAAGGAGATCACGCTGGAATTGTTCAGGTCATGTTGTCCCAGCCAAGCACCGAGCCTCAGCTCGGCGCGCCGGCAAGCCCCGACGACAGTCCGCTGGACTTCAGCGCCGTCGACGTGCGGCTGCCGATGCTGGTGTACGTGTCCCGGGAGAAGCGCCCGGGCTATGACCACCAGAAGAAGGCGGGCGCGCTGAACGTGCAGCTGCGCGTCTCCGCTCTGCTCTCCAACGCGCCCTTCATCATCAACTTCGACTGCGACCACTACATCAACAACTCCCAGGCCTTCCGCGCCGCCATGTGCTTCATGATAGACCGGCGCGACGGCGACAACATCGCCTTCGTCCAGTTCCCGCAACGCTTTGACGACGTCGACCCTACGGACCGCTACGCCAACCACAACCGCATGTTCTTCGACGCCACCATGCTCGGCATGAACGGCATCCAGGGGCCCTCCTACGTCGGCACCGGCAGCATGTTCCGCCGGGTCGCGCTCTACGGCGCCGACCCGCCTCGGTGGCGCCCGGACGACGTCAAGGTGCTGGAGAACCCCAACAAGTTCGGCACGTCGATGACGTTCATCAACTCCATACCGGTGGCCGCGAACCAGGAGCGGTCCGTCATGTCGCCGGTGTCGCTTGAAGAGCCGGCAACAACGGAGATGGCCGACGTCATGACGTGCGCGTACGAGGACGGGACGGAGTGGGGCGATGGCGTCGGCTGGGTGTACGACATGGCAACGGAGGACGCGGTGACCGGATTCCGGCTGCACCGGACGGGGTGGCGGTCCATGTACTGCGACATGGAGCCCCCCGCGTTCCGCGGCACGGCGCCGATCAACATGACGGAGCGCATGTACCAGATCCTGCGCTGGTCGGGGGGCTCCTTGGAAGTGTTCTTCTCCCGCTTCTGCCCGCTCCTCGCCGGCGGCCGGCTCCACCCCATGCAGCGCGTCGCCTACACCAACATGACCTTCTACCCGCTCTCGGCGCTCTTCGTCGTCTGCTACCATCTCCTGCCGCTGATGTGGGTCTTCAACGGCCGCTTCTACATCCAGAAGCCCTACCCGACGTACGTGATGTACGTGCTCGTCATCATCGTCTCCAACGAGGTGATCGGCATGGTGGAGATCGTGTGGGCGGGGCTCACGTTGCTCGACTGGTTCCGCAACGAGCAGTTCTACATGATCTGTGCGACGGGCGTGTACCCGACGGCGGTGCTGCACGTCGTGCTCAGGTCCCTCGGCCTCAAAGGCATGTCCTTCAAGATGACGGCCAAGCAGCTGACCACGGGCGCGAGGGAGAGGTTCGCCGAGCTGTACGACGTGCAGTGGGCGCCGCTGCTGATACCCACACTGGTGGTCATCGCCGTTAACGTGGTTGCCATCGGCGCGGCGGTGGGGAAGGCGGTCACCTGGGGCTGGTCGGCCGGGCAGGTCGTCGAGGCGGCGAGCGGGCTCATGTTCAACGTCTGGATCCTGCTCATGTTCTACCCTTTCGCGCTCGGGGTTATTGGGCGCTGGGGCAAGAGGCCATATGTCCTCTTCGCCATGTTTATGGCCGCGTTCGCCGCCATTGCTGCGGTGTACGTCGCCGTCCTGGCGGCGCTCGCTGGCAACCTGCCCTACTTCCAGCTGGTACACTGGTCGGTCGGA

>HvCSLF13

ATGAACGTGATGCTCCGTGTCTCCGCGCTGCCCTCCAACGCGCCCTTCGTCATCAATTTTGACTGTGACCACTACATCAACAACTCGCAAGCTCTCCGTGCCCCTATGTGCTTCATGCTCGACCCTCGTGATGGTCAGAACACGGCGTTCGTCCAGTTCCTGCAGCGGTTTGACGACGTCGACCCGACTGATCGCTACGCCAACCACAACCGTGTCTTCTTCGACGGCACCATGCTCGCGCTCAACGGCCTCCAGGGGCCTACCTACCTCGGCACCGGCACCATGTTCAGTCGACCGGTGAGTTCGGTTACTCGACGCCGTTCATCAACTTTGGTGAAGATCGACGAGGGCCTCAGCAAGGACTTAGCTACCTTGATGACATGCGCTTACGAGGACGGGAGCTCATGGGGGAGAGATGCTGGATGGGTGTACAACATCGCGACGGAGGACGTGGTGACTGGATTCCGCATCCATCGGCAAGGGTGGCGTTCCATGTACTGCTCCATGGAACCTGCGGCGTTTCGCGGAACGGCTCCGATCAATCTCACCGAGCGCCTCTACCAGGTGCTTCGGTGGTCGGGCGGCTCCCTCGAGGTGTTCTTCTCCCACAGCAACGCCCTCATCGCCAGCCGCCGGCTCCACCCTCTGCAGCGCATCGCCTACCTCAACATGTCGACGTACCCGATCGTCACGGTGTTCATCCTGGCGTACAACTTCTTCCCTGTCATGTGGCTCTTCTCCGAGCAGCTCTACATCCAGAGGCCGTTCGGCACGTACATGGCGTACCTCATCGCCGTCATAGCCATGGTGCACATGATCGGCATGTTCGAAGTGAGATGGTCAGGGATCACACTGCTCGACTGGTTCCGCAACGAGCAGTTCTACATGATCGGGGCGACGGGCGTGTACCCGACGGTGGTGCTCTACATGTTGTGGAAGCTCGTCACGGACGGAGGCATGCTCCAACGACAAGTTCGCCGACCTCTACACAGTACGGTGGGTGCCGCTGCTGGTCCGACCGCCGCGGTGATTGTCGTCAACGTCGCGGCTGTCGGGGCGGCGATAGGCAAGGCGGCGACATGGGGCTTCTACACAGATGAGGCGTGGCACGCACTCCTCGGCATGGTGTTCAACGTCGGGATCCTCGTGCTTCTCTACCCGTTTGCACTCGGCATCATGGGGAAGTGGGGGAAGAGACCCATCATCCTGTTCGTCATGTTGGTCATGGGCATTGGCGCCGTCGGGCTCTTATATGTCACGCTCCACGCCCCGTACCCAGGAGACTGGTCACAAGTTTCCGTTCCTCTTGGTAAAGCG

>Bradi5g10130

ATGGCGGGCGCCAGGAAGAAGCTGCAGGAGAGGGTGGCCCTCCGGAGAACGGCGTGGAAACTGGCCGACATCGTAGTCCTCTTCCTCCTGCTTGCCCTCCTGGCCCGCCGCGCCGCCACGCTAGGGGACACGTCGACGACGTGGCAATGGCCATGGCTCGCGGCGCTCGTCTGCGAGGCCTGGTTCACCTTCGTGTGGCTCCTCACCATCAACGGCAAGTGGAGCCCCGTCCGCTTCGACACCTTCCCCGAACACCTCCTCGAGGCCGACGACGAGCTCCCGGCGGTGGACATGTTCGTGACCACGGCGGACCCGGCGCTGGAGCCACCGGTGATCACGGTCAACACGGTGCTCTCGCTGCTCGCCGTCGACTACCCCGACGCCCGCAAGCTGGCGTGCTACGTCTCCGACGACGGCTGCTCCCCGGTGACGTGCTACGCGCTGAGCGAGGTCGCCGCGTTCGCCGCGCTCTGGGTGCCCTTCTGCAAGCGGCATGCCGTCGGGGTCAGAGCCCCTTTCATGTACTTCTCTTCCGCTCCGGATGAGGCTGGCAGCCACGGCGCCGACTTCTTGGAAAGCTGGGCATCCATGAAGAGCGAGTATGACAAGCTAGCCAGCCGAATCGAGAACGCCGACGAGGGGTCCATTCTGCAGGACGCCGAGTTCGCCGAGTTCGTTGGCTCCGAGCGCAGGAACCATCCTACCATCATTAAGGTTCTCTGGGATAACAGCAAGAGCAAGACAGGGGAAGGGTTCCCACATCTCGTGTACGTCTCGAGAGAGAAGAGCCCGAGACATTACCACAACTTCAAGGCCGGCGCCATGAACGTTCTGACAAGGGTATCGGCTGTGATGACCAACGCGCCAATCATGCTGAACGTGGATTGCGACATGTTCGCCAACAATCCAGGGGTCGCCCTGCATGCCATGTGTCTCCTCTTGGGGTTTGACGACGAGACCGACAGCGGGTTCGTCCAGGCGCCGCAGAAGTTCTACGGTAGCCTCAGGGATGATCCCTTTGGTAACCAGATGGAGGTTTTGTTCCAGAAACTTGGAGGTGGAGTCGCTGGGATCCAAGGCATGTTCTACGGCGGCACCGGGTGTTTTCACCGCAGGAAAGTCATATACGGCACGCCACCACCAGACACCGTTAAACATGGGACAACAGGTTCACCATCTTACAAGGAGCTGCAAATGAAGTTTGGGAATTCGAAAGAATTGATCGACTCATCTAGGAGCATCATCTCCGGAGATGTGCTCGCTAGAACAACAGCAAATATGTCAAGCCGCATCGAAATGGCAAAACAAGTGGGCGCCTGCAACTACGAGGCTGGTACATGTTGGGGCCAGGAGGTTGGCTGGGTCTACGGGTCTATGACAGAAGACATCTTGACCGGGCAGCGAATCCATACAACAGGTTGGAAATCCGTGTTGATGGACACCAACCCACCGGCGTTCCTAGGATGCGCTCCGACCGGGGGACCAGCCAGCTTGACCCAGTTCAAGAGATGGGCAACAGGGGTTTTGGAGATACTCATCAGCAAGAACAGCCCGATCCTCGGCACCATCTTCGGGCGCCTCCAGCTCCGGCAGTGCCTCGCTTACCTGATTGTAGATGTGTGGCCAGTGAGGGCGCCTTTCGAGCTGTGCTATGCGCTATTGGGGCCTTTCTGCCTCCTCGCAAACCAGTCCTTCTTGCCAAAGGCGTCGGATGAAGGTTTCTATATCCCAGTGGCTCTGTTCTTGACCTTCCAGGTATACTACTTGATGGAGTACAAGGATTGCGGGCTCTCGGCCCGTGCATGGTGGAACAACCACAGGATGCAACGCATCACGTCAGCCTCCGCCTGGCTCCTTGCGTTCCTCACGGTGCTCCTCAAGACCATGGGACTGTCCGAAACCGTGTTCGAGGTCACCCGCAAGGAGAGCAGCACATCTGATGGCAGTGGGAGCACCAACGAGGCTGACCCAGGGCTGTTCACCTTCGATTCATCGCCCGTGTTCATCCCAGTGACGGTGCTCGCAATCCTGAATATTGTCGCCATCGCTGTCGGTGTATGGCATGCTGTTGTTACCGGGACGGTGAAGGGTATTCATGGTGGCCCAGGAATCGGAGAATTCCTCTATTGTTGTTGGACGGTGCTGTGCTTCTGGCCCTTCGTGAGAGGGCTTGTTAGCAGGGGGAGGTATGGGATCCCGTGGAGTGTCAAGGTGAAGGCTGGTTTGATTGTGGGTGCGCTTGTGCACTTCTGCACAAGGAAC

>OsCSLH1

ATGGAGGCGGCGGCTAGAGGCAACAAGAAGCTGCAGGAGAGGGTGCCCATCCGGCGCACCGCGTGGAGGCTCGCCGACCTCGCCATCCTCTTCCTCCTCCTCGCCCTCCTCCTCCACCGCGTCCTCCACGACAGCGGCGCGCCATGGCGGCGCGCCGCGCTCGCCTGCGAGGCGTGGTTCACCTTCATGTGGCTGCTCAACGTGAACGCCAAGTGGAGCCCCGTCCGTTTCGACACGTTCCCGGAGAACCTCGCCGAAAGGATCGACGAGCTCCCGGCGGTGGACATGTTCGTGACGACGGCGGACCCGGTGCTGGAGCCGCCGCTGGTGACCGTGAACACGGTGCTGTCGCTGCTCGCCCTCGACTACCCGGCCGCCGGCGAGAAGCTGGCGTGCTACGTCTCCGACGACGGGTGCTCGCCGCTGACGTGCTACGCGCTGCGGGAGGCCGCCCGGTTCGCCAGGACGTGGGTGCCCTTCTGCCGGCGGCACGGCGTCGCCGTCAGGGCGCCCTTCCGGTACTTCTCCTCCACGCCGGAGTTCGGCCCGGCGGATGGCAAGTTCTTGGAGGACTGGACATTCATGAAGAGCGAGTATGAGAAGTTGGTCCACCGGATCGAGGACGCCGATGAGCCTTCCCTTCTGCGGCACGGCGGTGGTGAGTTCGCAGAGTTTCTGGATGTTGAGAGGGGGAACCACCCTACTATCATAAAGGTTCTGTGGGATAACAACAGGAGCAGGACAGGAGATGGCTTCCCTCGTCTGATATACGTCTCAAGGGAGAAGAGCCCCAACCTACACCATCACTACAAGGCTGGCGCCATGAATGCCCTGACAAGGGTGTCAGCACTGATGACCAACGCCCCATTCATGCTAAACCTAGACTGCGACATGTTTGTAAACAACCCCCGGGTCGTCCTCCATGCCATGTGCCTTCTGTTAGGTTTTGACGATGAGATCAGCTGCGCGTTTGTTCAGACGCCGCAGAAATTCTACGGTGCCTTGAAGGATGATCCTTTCGGGAACCAGCTGGAAGTTAGTTTGATGAAAGTTGGACGTGGGATTGCAGGGCTTCAGGGCATATTTTATTGTGGAACAGGCTGCTTTCACCGCAGAAAAGTCATTTACGGCATGAGGACAGGGCGAGAAGGCACCACAGGTTATTCATCTAACAAGGAGCTCCATAGTAAATTCGGAAGTTCAAATAATTTTAAGGAATCAGCCAGGGATGTCATTTATGGGAACTTGTCAACAGAGCCAATAGTAGATATATCAAGTTGCGTTGATGTTGCCAAAGAAGTAGCTGCCTGCAACTACGAGATTGGCACATGTTGGGGTCAGGAGGTTGGTTGGGTCTATGGATCACTGACAGAAGACGTGTTGACCGGACAACGGATCCATGCAGCGGGTTGGAGATCCACGCTGATGGAAATCGAACCACCAGCATTCATGGGTTGTGCACCAAATGGAGGGCCCGCCTGCCTAACCCAGTTGAAGAGATGGGCATCAGGTTTTTTAGAAATACTCATCAGTCGGAATAACCCAATCCTCACAACCACATTTAAGAGTCTCCAATTCCGACAATGCCTTGCATACCTGCACAGCTATGTGTGGCCTGTGAGGGCACCTTTCGAATTGTGCTATGCATTGTTGGGGCCTTATTGCTTACTATCAAACCAATCCTTCTTGCCAAAGACATCAGAAGACGGTTTCTACATCGCATTAGCTCTATTCATTGCCTATAACACATACATGTTCATGGAGTTCATAGAGTGTGGGCAGTCTGCACGTGCATGTTGGAACAACCACAGGATGCAACGGATCACCTCAGCTTCTGCTTGGCTACTGGCATTTCTTACCGTCATCCTCAAGACCTTAGGCTTCTCCGAGACTGTGTTCGAGGTCACCCGCAAAGACAAGAGCACATCAGATGGTGATTCCAACACCGATGAGCCTGAGCCAGGGAGGTTCACCTTCGACGAATCGACGGTGTTCATTCCCGTGACAGCACTTGCAATGTTAAGTGTCATTGCAATCGCTGTAGGAGCATGGAGGGTGGTTTTGGTGACAACGGAAGGCTTGCCCGGTGGCCCTGGTATCAGTGAATTCATCTCCTGTGGGTGGCTGGTGCTGTGCTTCATGCCATTGCTGAGAGGTCTAGTGGGAAGTGGTCGATATGGCATTCCTTGGAGTATCAAGATGAAGGCCTGCTTGCTTGTTGCTATATTCTTGCTCTTCTGCAAAAGAAAT

>OsCSLH2

ATGGCGGTGGTGGCGGCGGCGGCGGCCACCGGTTCCACCACCAGATCAGGCGGCGGCGGCGGCGAGGGGACGAGGTCCGGGAGGAAGAAGCCGCCGCCGCCGCCGCTGCAGGAGAGGGTGCCCCTCGGGCGGCGCGCGGCGTGGGCGTGGCGGCTGGCCGGCCTCGCCGTCCTCCTCCTCCTCCTCGCCCTCCTCGCCCTCCGGCTGCTTCGCCACCACGGCGGCGCCGGGGGCGACGGCGGCGTGTGGCGCGTGGCGCTCGTGTGCGAGGCGTGGTTCGCGGCGCTGTGCGCGCTCAACGTGAGCGCCAAGTGGAGCCCCGTCCGGTTCGTCACGCGGCCGGAGAACCTCGTGGCGGAGGGCAGGACGCCGTCGACGACGGCGGCGGAGTACGGCGAGCTGCCGGCGGTGGACATGCTGGTGACGACGGCGGACCCGGCGCTGGAGCCGCCGCTGGTGACGGTGAACACGGTGCTCTCGCTGCTCGCCCTCGACTACCCGCGCGCCGGCGAGCGGCTGGCCTGCTACGTCTCCGACGACGGGTGCTCGCCGCTGACGTGCCACGCGCTGCGGGAGGCCGCCGGGTTCGCCGCCGCGTGGGTGCCCTTCTGCCGCCGGTACGGCGTCGCCGTCAGGGCCCCGTTCCGGTACTTCTCCTCCTCCTCCTCGCCGGAGTCCGGCGGCCCGGCCGATCGCAAGTTCTTGGACGACTGGACATTCATGAAGGATGAGTATGACAAGTTAGTGCGGCGCATCAAGAACACCGACGAGCGCTCCCTCCTCCGGCACGGCGGCGGCGAGTTCTTCGCCGAGTTCTTGAACGTCGAGAGGAGGAATCACCCCACCATCGTCAAGACGAGGGTGTCAGCTGTGATGACCAACGCACCGATCATGCTGAACATGGACTGCGACATGTTTGTGAACAATCCCCAGGCCGTCCTCCATGCGATGTGCCTGCTGCTGGGGTTCGACGACGAGGCCAGCAGCGGGTTCGTCCAGGCGCCGCAGAGATTCTACGACGCCCTCAAGGACGATCCATTTGGGAACCAGATGGAGTGTTTTTTCAAGAGATTTATCAGTGGGGTTCAAGGAGTTCAGGGTGCCTTTTATGCTGGAACCGGCTGCTTTCACCGTAGGAAAGCAGTTTATGGCGTGCCACCGAACTTCAATGGAGCCGAGAGAGAAGATACCATAGGTTCATCGTCTTATAAGGAGCTTCATACCAGGTTTGGAAACTCAGAGGAATTGAACGAATCAGCAAGAAACATCATTTGGGATCTGTCCTCTAAGCCAATGGTTGATATATCAAGTCGCATTGAAGTGGCAAAAGCAGTTTCAGCTTGCAACTATGATATTGGCACATGTTGGGGACAGGAGGTTGGTTGGGTCTATGGATCACTAACAGAAGACATATTGACCGGACAGCGGATACACGCGATGGGTTGGAGATCCGTATTGATGGTAACCGAACCACCCGCATTCATGGGCTCCGCGCCGATTGGAGGACCAGCCTGCCTAACTCAGTTCAAGAGATGGGCAACTGGCCAATCTGAGATAATCATCAGCCGGAACAACCCAATTCTCGCAACCATGTTCAAGCGCCTCAAATTCCGGCAATGTCTTGCCTACCTGATCGTCCTTGGGTGGCCTCTGAGAGCGCCTTTTGAGCTATGCTATGGATTGTTGGGACCTTATTGCATACTCACAAACCAATCCTTCTTGCCAAAGGCATCAGAAGATGGTTTCAGCGTCCCGTTAGCCCTATTCATATCCTATAACACATACAACTTCATGGAGTACATGGCGTGCGGGCTCTCCGCCCGTGCATGGTGGAACAATCATAGGATGCAACGGATCATCTCGGTCTCTGCCTGGACACTAGCATTTCTTACCGTGCTCCTCAAGTCCTTAGGCCTCTCCGAAACTGTTTTTGAGGTCACCGGCAAGGACAAAAGCATGTCTGATGACGATGACAACACCGATGGTGCTGACCCTGGGAGGTTCACCTTCGACTCATTGCCGGTGTTCATCCCCGTGACGGCACTTGCGATGTTAAACATCGTTGCGGTCACTGTCGGAGCATGTAGGGTAGCTTTCGGGACAGCGGAAGGTGTGCCCTGTGCCCCGGGTATCGGCGAATTCATGTGTTGTGGGTGGCTGGTGCTGTGCTTCTTCCCGTTTGTAAGAGGGATAGTGTGGGGCAAGGGAAGCTATGGGATCCCTTGGAGTGTCAAGCTGAAGGCTAGCTTACTTGTGGCTATGTTTGTTACCTTCTGCAAAAGAAAC

>OsCSLH3

ATGGCGGCGGCGAGCGGCGAGAAGGAGGAGGAGGAGAAGAAGCTGCAGGAGAGGGCGCCGATCCGGCGCACGGCGTGGATGCTGGCCAATTTCGTCGTACTCTTCCTCCTCCTCGCCCTCCTCGTCCGCCGCGCCACCGCCGCCGACGCCGAGGAGCGCGGCGTCGGCGGCGCGGCGTGGCGCGTGGCGTTCGCCTGCGAGGCGTGGTTCGCGTTCGTGTGGCTGCTCAACATGAACGCCAAGTGGAGCCCCGCCCGGTTCGACACCTACCCGGAGAACCTCGCCGGAAGGTGTGGCGCCGCCCATCGTCCTAGAAAGTCGTCGTGCATCTCCGGCCATCTCGATCTCATGCGGAGACAGTGTGCTTTGATGCAGGATCGACGAGCTGCCGGCGGTCGACATGTTCGTGACGACGGCGGACCCGGCGCTCGAGCCGCCGGTGGTGACGGTGAACAAGGTGCTCTCGCTGCTCGCCGTCGACTACTACCCGGGCGGCGGCGGCGCCGGCGGCGGCGAAGGCTGGCCTGCTACGTCTCTGACGACGGGTGCTCGCCGGTGACGTACTACGCGCTGCGGGAGGCCGCCGGGTTCGCGAGGACGTGGGTGCCCTTCTGCCGGCGGCACGGCGTCGCCGTCAGGGCCCCCTTCCGGTACTTCGCCTCCGCGCCGGAGTTCGGCCCGGCCGACCGGAAGTTCTTAGACGATTGGACATTCATGAAGAGTGAGTACGACAAGCTAGTCCGTCGGATCGAGGACGCCGACGAGACCACCCTTCTGCGGCAAGGCGGCGGCGAGTTCGCCGAGTTCATGGACGCCAAGAGGACGAACCACCGCGCCATTGTCAAGGTTATATGGGATAATAACAGCAAGAACAGGATAGGCGAAGAAGGAGGGTTCCCGCATCTCATATACGTCTCAAGGGAGAAGAGCCCCGGACACCACCATCACTACAAGGCCGGCGCCATGAACGCCCTGACGAGGGTGTCAGCCGTGATGACCAACGCACCGATCATGCTGAACGTGGACTGCGACATGTTCGCGAACGATCCCCAGGTCGTCCTCCACGCGATGTGCCTGCTGCTGGGGTTCGACGACGAGATCTCCAGCGGGTTCGTTCAGGTGCCGCAGAGTTTCTACGGCGACCTCAAGGACGATCCTTTCGGGAACAAGCTGGAGGTTATTTACAAGAAACTTCTAGGCGGGGTTGCAGGGATT

>HvCSLH1

ATGGCGGGCGGCAAGAAGCTGCAGGAGAGGGTCGCCCTGGCGAGAACCGCGTGGATGCTGGCCGACTTCGCGATCCTCTTCCTCCTCCTCGCCATCGTGGCCCGCCGCGCCGCCTCGCTCCGGGAGCGCGGCGGGACGTGGTTGGCGGCGCTCGTCTGCGAGGCGTGGTTCGCCTTCGTGTGGATCCTCAACATGAACGGCAAGTGGAGCCCCGTCCGGTTCGACACCTACCCCGACAACCTCGCCAACAGGATGGAGGAGCTCCCGGCGGTGGACATGTTCGTCACGACCGCGGACCCGGCGCTGGAGCCTCCGTTGATCACGGTGAACACGGTGCTCTCGCTGCTCGCCCTGGACTACCCGGACGTCGGCAAGCTGGCGTGCTACGTCTCTGACGACGGCTGCTCCCCGGTGACGTGCTACGCGCTGCGTGAGGCCGCCAAGTTCGCCGGCCTCTGGGTCCCTTTCTGCAAGAGGCACGACGTTGCTGTGAGGGCCCCATTCATGTACTTCTCTTCCACGCCGGAGGTTGGCACAGGCACAGCCGACCACGAGTTCCTGGAAAGCTGGGCGCTCATGAAGAGCGAATATGAGAGACTAGCCAGCCGAATCGAGAACGCCGATGAGGGCTCCATTATGCGTGACAGCGGCGACGAGTTCGCCGAGTTCATCGACGCCGAGCGCGGGAACCATCCTACCATCGTTAAGGTTCTGTGGGATAACAGCAAGAGCAAAGTGGGGGAAGGATTCCCACATCTGGTGTACCTCTCTCGAGAGAAAAGCCCCAGACATCGCCACAACTTCAAGGCTGGTGCCATGAATGTTCTGACAAGGGTGTCAGCCGTGATGACCAACGCTCCCATCATGCTGAATGTGGACTGCGACATGTTCGCCAACAATCCGCAGGTCGCCCTACACGCGATGTGCCTCCTATTGGGGTTCGACGACGAGATCCACAGCGGGTTCGTCCAAGTGCCACAGAAGTTCTACGGTGGCCTCAAGGACGATCCCTTTGGCAACCAGATGCAGGTTATAACCAAGAAAATTGGAGGTGGAATCGCCGGGATCCAAGGCATGTTCTACGGCGGCACGGGCTGTTTTCACCGCAGGAAAGTCATTTACGGCATGCCGCCACCTGACACCGTCAAACACGAGACAAGAGGTTCACCATCTTACAAGGAGCTGCAAGTCAGGTTTGGGAGCTCAAAGGTGTTGATCGAATCATCTAGGAACATCATCTCAGGAGACCTGCTCGCTAGACCAACCGTTGATGTATCGAGTCGCATCGAAATGGCAAAACAAGTCGGCGATTGCAACTATGAGGCTGGCACGTGTTGGGGCAAGGAGATTGGTTGGGTCTATGGATCAATGACAGAAGACATTTTGACCGGACAACGGATCCATGCGGCGGGTTGGAAATCGGCCTTGTTGGACACCAACCCACCGGCATTCTTGGGATGTGCTCCGACCGGGGGACCGGCCAGCTTGACCCAGTTCAAGAGATGGGCAACAGGGGTTCTGGAGATACTCATCAGCCGGAACAGCCCTATCCTCGGCACCATCTTCCAACGCCTCCAACTCCGGCAATGCCTTGGCTATCTCATCGTCGAGGCGTGGCCCGTGAGGGCGCCTTTCGAGCTGTGCTATGCACTATTGGGACCTTTCTGCCTTCTCACAAACCAGTCCTTCTTGCCAACGGCATCGGATGAAGGTTTTCGCATCCCAGTAGCTCTATTCTTGAGTTACCACATATACCACTTGATGGAGTACAAGGAGTGCGGGCTCTCTGCCCGCGCCTGGTGGAACAACCACAGGATGCAACGCATCACCTCGGCCTCCGCCTGGCTCCTCGCCTTCCTCACCGTGATCCTCAAGACACTAGGGCTCTCTGAGACCGTGTTCGAGGTCACCCGCAAGGAAAGCAGCACGTCCGATGGCGGCGCCGGCACCGACGAGGCCGATCCAGGACTGTTCACATTCGACTCGGCGCCCGTTTTCATCCCGGTGACGGCGCTCTCAGTGTTGAACATTGTGGCCCTCGCCGTCGGGGCATGGCGCGCCGTCATCGGGACTGCGGCGGTCGTTCATGGTGGCCCGGGCATCGGAGAGTTCGTGTGCTGTGGCTGGATGGTGTTGTGCTTCTGGCCGTTCGTGAGAGGGCTTGTCAGCAGGGGAAAGCATGGAATCCCGTGGAGCGTCAAGGTGAAGGCTGGTTTGATTGTGGCTGCGTTCGTGCACCTCTGCACAAGGAAC

>HvCSLJ

ATGGCGGCGCCGTCGCAGGACGCGCCGCTCCAGCTCAACACCGTGCAAACGGACCAGCCCCTCGCCACCGTCAACCGCCTCCTCGCCGCCCTCCACGTGGCGCTCGCCGCCGCGGCCATCGCCCACCGCGGCGCCCACGTGATGCTGGCGGCCGACCTGGCGCTCCTCTTCCTGTGGGCGCTGTCGCAGGCGCCCATGTGGCGTCCCGTCTCCCGCGCCGCCTTCCCGTCCCGGCTCTCGCGCGCCGCCCTCCCGGCCGTGGACGTGATGGTGGTGACGGCCGACCCGGACAAGGAGCCCGCGGCGAAGGTGATGAGCACGGTGGTCTCCGCCATGGCGCTCGACTACCCGGGCGGGCGGCTCAGCGTGTACCTCTCCGACGACGCCGGGTCGCCGCGGACCCTGCTCGCTGCCAGGAAGGCCTACGCCTTCGCCAGGGCCTGGGTGCCCTTCTGCAGGAAGTACGGCGTGCGGTGCCCGTGCCCCGACAGGTTCTTCGCCGGCGACGACCAGCTCGACCTCGGCGGTCACCACCGCCAAGAGCTCGACGACGACATGCTGAGGATCAAGAACATGTATGAGACGTTCAACGAGGGTGTGGAGGAGGTGATGAACGACGCAGCTCTTTCTCAGAGTTGGATAAAAGCAGATCACGACGCCCACATCGAGATGATGACCGACGGCAGCAACATCGACTCCGGGGACGAGGATGAGGACGCCATGCCCTTGCTGGTCTACGTGTCCCGCGAGAAGCGGAGGCCGTCGGCTCACCACTTCAAAGCCGGCGCACTCAACGCCCTCCTCCGGGTGTCGAGCCTGATGAGCAACTCGCCGTACGTGATGGTGCTGGACTGCGACATGTACTGCAACAGCAGGAGCTCCGTCCTCGAGGCCATGTGCTTCCACCTCGACGGCCGCCGCCCCGCCGACCTCGCCTTCGTGCAGTTCCCTCAGATGTTCCACAATCTCAGCAGTAGCGACATCTACGCCAACGAGCTCAGGTCCATCTTCTGGACGCGGTGGAAAGGTTTGGACGGCCTCCGGGGCCCGATCCTCTCCGGCACCGGCTTCTGCGCCCGAAGAGACGCCCTCTACGGCGCCCGGCCGGCCAGCTCGCAGGGTCAGTTCTCCGGCATGGAGGTCGGCGAGCTGAAGAGAAGGTTCGGCGTCTCCAATGGTCACATAGCGTCGCTGCGCCGGTCGGCCGGGAACGGGAGCACGACGGTTGCGCGTGATGTTCTCCCACAGGATGCAGAGTTCGTGGTGTCCTGTGCGTACGAGCCGGGCACGGAATGGGGCGAGGAGGTCGGCTTCTTGTACCAGTCGGTGGTGGAGGACTACTTCACCGGCTACCGGCAGCTCTACTGCCGAGGATGGACGTCCGTCTACTGCTTCCCGGCGACGGCGTCGAGGCCGCCGTTCCTCGGCAGCGTGCCCACCAACCTCAACGACGCGCTGGTGCAGAACAAGCGGTGGATGTCCGGCATGCTCGCCGTCGGCCTCTCCAGGCACTGCCCCTTCGCCTCCGCCGCCGTCTCCGTGCCTCAGAGCATGGGCTTCGCCTACTACGCCTTCATGGCCCTGTACGCCTTCCCCGTGCTCTGCTACGCCACCGTGCCGCAGCTCTGTTTCTTCCGTGGCGGCACGTCGTTTCCCGGGGCGTCGACGCCCTGGTTCGGCGCCGTGTTCGTGTCGTCGTCGCTGCAGCACCTGGTGGAGGTGTCGGTGGCGAAGCGCGGGCTGGCGGCGAGGACGTGGTGGGACGAGCAGAGGTTTTGGGCGCTCAACGCCGTCACGGGCCAGCTCTTCGCCTGCCTCCGCGTTGCCCTCAGCCTGGCCGGCGCCGGCAGCCGGGCGGTGGACTTCGACCTCACCAGCAAGGCGTCCGACGACAGGCTGTACCGGGACGGCGTGTTCGACTTCGCCGGATGCTCGGCGCTGCTCCTGCCGGCCACCACGCTCTGCCTGCTCAACGCCGCAGCGCTCCTCGGCGGGGTGTGGAAGATGGTCGGCGGGGGCGGCAGCGTGTCCGGCGAGCTGTTCCTCCTCTGCTACGTCGTGGCACTGAGCTATCCGCTGCTGCAGGGGATGTTCCTCAGGCGGGATGCTGCAAGAGTTCCAGCGCGGATCACGGCAATATCCGTCGCCATGGTTGCCGCTCTGCTTTGCTTGTTTGGT

>Sb06g016770

ATGGCGAGCGCCAAGACGAAGCTGCAGCTGCAGCTGCAGGAGAGGGTCCCGCTCCCCCGCACGGCGTGGAAGCTGGCCGACCTCGCCGTTCTCTTCCTGCTCCTGGCCCTCCTCGCTCACCGTGTGTCCTCGCTGATGGTGGCCGTCGGCGGCGGCGGCGGCGCGGCGTGGTGCTGTGTCGCCGCGCTCATCTGCGAGGCGTGGTTCACGGTGGTGTGGCTCCTCAACATGAACGCCAAATGGAACCCGGTCCGCTTCGACACGCACCCTGAACGCCTCGCCGAACGGATGAGCGACGGCGAGCTTCCGGCGGTGGACATGTTCGTGACGACGGCGGACCCGAAGCTGGAGCCGCCGCTGGTGACGGTGAACACGGTGCTGTCGCTGCTAGCGCTGGACTACCCCGCAGGCAAGCTGTCCTGCTACGTCTCCGACGACGGGTGCTCGGCGGCGACCTGCTACGCGCTGCGCGAGGCCGCCGAGTTCGCCAAGCTCTGGGTGCCCTTCTGCATGAAGCACGGCGTCAAGGTCAGGGCCCCCTTCGTCTACTTCTCCGAGCGTGGCGGGGCCGCAGCCGACGACGACGACGCTGAGTTCTTGCGCGCTTGGACGTCCATGAAGAACGAGTACGAGGAGTTGGTCCGCCGGATCGAGAACGCCGAGGAGTCTCTAGTCCGGCGCGGCGACGGCGAGTTCGCTGAGTTCGTGGGTGCTGACCGCAGGAATCACCCGACCATAATCAAGGTCCTGATGTGGGACAACAGCAGCAACGACGACAGCAAGAATGCTGCAGGCGATGGAATAATCCCAAGCCTCATATACGTCTCGCGGGAGAAGAGACCCACACAGCACCACTACTTCAAGGCCGGTGCCATGAACGTCCTGACGAGGGTGTCCGGCGTGGTGACCAACGCGCCCATCATGCTGAACGTGGACTGCGACATGTTCGCCAACAACCCGCAGGTGGCACTCCACGCCATGTGCCTCCTCCTGGGCTTCGACGACGAGCTCCACAGCGGCTTCGTTCAGGCGCCGCAGAAGTTCTACGGCGGCCTCAAGGACGATCCTTTCGGCAACCAGATGCAGGTCATATACGAGAAAGTTGGATTTGGAGTTGCCGGACTTCAAGGCATATTTTACGGCGGGACAGGTTGCTTTCACCGTAGGAAAGTCATCTACGGCGTGCCACCAGACTCCACCACCACCATCGGCATCAAAGATTCACCATCTTACAAGGAGCTGCATAAGAAGTTTGGCAGCTCGAAAGAACTGATCGAGTCGGCGAGGAGCATCATTTCAGGGGACATGTTCAGAACACCAACTGAGGTTGTGGCAGACCTAACGAGTCTCATCGAAGCAGCCAAACAAGTCTCCGCCTGCAGCTACGAGACCGGCACGAGCTGGGGTCAGGAGGTCGGCTGGGTCTACGGGTCCATGACCGAGGACGTCCTGACGGGGCAACGCATCCACGCCGCCGGCTGGAGGACGGCGTTGTTGAACCCCGACCCGCCGGCGTTCCTCGGCGGCGCGCCCACCGGCGGACCCGGCAGCCTCACCCAGTACAAGAGGTGGGCGACGGGCCTGCTGGAGATACTCCTCAGCCGCCATAACCCGTTCCTTCTCGCCGCGTTCAAGCGCCTCGATCTCCGGCAGTGCGTCGCCTACCTAGTCATCGACGTGTGGGCCATTAGGGCACCCTTTGAGCTGTGCTACGCGTTGCTCGGACCTTACTGCGTCATCGCAAACCACTCCTTCCTGCCAAAGGCGTCAGAACCGGGTTTCGTCATCCTGCTGGCCCTGTTCCTAGGTTACAACGTGTACAACCTGGGCGAGTACAAGGACTGCCGGCTCTCGGTGCGCGCCTGGTGGAACAACCACCGGATGCAGCGGATCGTGTCGTCCTCCGCCTGGCTGCTCGCGTTCCTCACCGTCGTCCTCAAGACGCTCGGCCTCTCGGAGACGGTGTTCGAGGTGACGCGCAAGGAGCAGCAGAGCACGTCTGATGGTGGTGCGGACGCCGTCGCCGACCCGGGGCGGTTCACCTTTGACTCGTCGCCGGTGTTCATCCCGCCGACAGCGCTCACGATGCTGAGCATCGTCGCCGTCGTCTTTGGGGCGTGGAGGCTGGTCGCCGGTGAGGGGGAAGGCGTGCCCAGTCCCAGTGGCCCGGGCGTCGGGGAGTTCGTGTGCTGCGGTTGGCTGGTGCTCTGCTTCTGGCCGTTCGTGAGGGGGCTCGTCAGCAAGGGAAGCTACGGCATCCCCTGGAACGTCAGGCTCAAGGCTGCCCTGCTTGTAGCCGCGTTCGTGCACTTGTCCACATGGAAG

>Sb06g016760

ATGGCGAGCGCCAAGACGAAGCTGCGGCTGCAGGAGAGGGTCCCGCTCCCACGCACCGCGTGGAAGCTGGCTGACCTTGCCGTTCTCTTCCTGCTCCTGGCCCTCCTCGCTCGCCGTGCGTCCTCGCTGGTGGCGGCTGGCGGTGCCGGCGCGGCGCCGGCGTGCACCTGGTGCTGGGTCGCCGCGCTCATCTGCGAGGCATGGTTCACGGTGGTGTGGCTCCTCAACATGAACGCCAAGTGGAACCCGGTCCGCTTCGACACGCACCCTGAACGCCTCGCCGAACGGACCGACGAGCTGCCGGCGGTGGACATGTTCGTGACGACGGCGGACCCGAAGCTGGAGCCGCCGCTGGTGACGGTGAACACGGTGCTGTCGCTGCTGGCGCTGGACTACCCCGCGGGCAAGCTGTCGTGCTACGTCTCCGACGACGGGTGCTCGGCGGTGACCTGCTACGCGCTGCGCGAGGCCGCCGAGTTCGCCAAGCTCTGGGTGCCCTTCTGCAAGAAGCACGGCGTCAAGGTCAGGGCCCCCTTCGTCTACTTCTCCGAGCGTGGCGGGGCCGCAGCCGACGACGACGACGACGTTGTTGAGTTCTTGCGCGCTTGGACGTCCATGAAGAACGAGTACGAGGAGTTGGTACGCCGGATCGAGAACGCCGAAGAGTACTCTTTAGTCCGGCGTGCCGACGGCGAGTTCGCCGAGTTCGTGGGTGCTGACCGCAGGAATCACCCGACCATAATCAAGGTGCTCTGGGACAGCAGCAATCAGGATGCTGCAGGCGATGGGATCCCAAGCCTCGTATACGTCTCCAGGGAGAAGAGCCCCACACAGAACCACCACTTCAAGGCCGGAGCCATGAACGTCCTGACGAGGGTGTCCGGCGTGGTGACCAACGCTCCCATCATGCTGAACGTGGACTGCGACATGTTTGCAAACAACCCGCAGGTGGCTCTCCACGCCATGTGCCTTCTCCTGGGCTTCGATGACGACGTCCACAGCGGCTTCGTCCAGGCGCCGCAGAAGTTCTACGGCGCCCTCAAGGACGACCCTTTCGGGAACCAGCTGCAGGTCATATTCGAGGTAACGAAAGTCATGTACGGCGTGCCACCAGACAACGCCGCCGCCACCACCACCAGCATGAAAGATTCACCGTCTTACAAGGAGCTACAGAACAGGTTTGGCAGATCGAATGAACTGATCGAGTCAGCGAGGAGCATCATTTCAGGGGACATGTTCAGAATCAGAACACCAACTGTGGTGGTGCCAGACCTAACGAGTCGCATCGAAGCAGCCAAACAAGTCTCTGCCTGCAGCTACGAGACCGGCACGAGCTGGGGTCAGGAGGTTGGCTGGGTCTACGGGTCCATGACCGAGGACGTTCTGACCGGGCAGCGCATCCATGCCGCTGGCTGGAGGTCGGCGATCCTCAACCCTGACCCGCCGGCGTTCCTCGGCGGTGCGCCCACCGGCGGGCCGGCAAGCCTCACCCAGTACAAGAGATGGGCGACGGGCCTGCTGGAGATACTCCTCAGCCGCCATAACCCTATCCTCCTCTCCGCGTTCAAGCGCCTCGATTTCCGGCAGTGCGTCGCCTACCTAGTCATTGACGTGTGGCCCGTCAGGGCACCTTTTGAGGTGTGCTACGCATTGCTCGGACCTTACTGCATCATCGCAAACCACTCCTTCCTGCCAAAGGTTACGGCGTCAGAACCGGGTTTCCTCATCCTGCTGGTCCTGTTCCTAGGTTACAACGTGTACAACTTGGGTGAGTACAAGGACTGCCGGCTCTCGGTGCGCGCCTGGTGGAACAACCACAGGATGCAGCGGATCGTGTCGTCCTCCGCCTGGCTGCTCGCGTTCCTCACCGTCGTCCTCAAGACGCTCGGCCTCTCGGAGACGGTGTTCGAGGTGACGCGCAAGGAGCAGAAGAGCTCGTCTGATGGTGGTGCGGACGCCGACGACGCCGACCCGGGGCGGTTCACCTTTGACTCGTCGCCGGTGTTCGTCCCGCCGACAGCGCTCACGATGCTGAGCATCGTCGCCGTCGCCGTTGGAGCGTGGAGGCTGGTCGCCGGTGCGGGGGAAGAAGGCGTGTCCGGCGGCTCGGGCGTCGGGGAGCTCGTGTGCTGCGGCTGGCTGGTGCTCTGCTTCTGGCCGTTCGTGAGGGGGCTGGTCGGCGGCAGGGGGAGCTACAGCATCCCCTGGAGCGTAAGGCTGAAGGCTGCCCTGCTCGTGGCCGCGTTCGTGCACTTGTCCACACGGAAG

>Sb06g016750

ATGGCGGGCATCAAGGCGAAGCTGCAGCTGCAGGAGAAGGTCCCGCTCCCACGCACGGCGTGGAAGCTGGCCGACCTCGCCGTTCTCTTCCTTCTCCTGGCCCTCCTCGCTCACCGTGTGTCCTCGCTGATGGTGACCAGCGGCAGCGGCGCGGCGGCGGCGTGCACGTGGTGCTGGGTCGCCGCGCTCATCTGCGAGGCGTGGTTCACGGTGGTGTGGCTCATCAACATGAACGCCAAATGGAACCCGGTCCGCTTCGATACGCACCCTGAACGTCTCGCCGGACGCAGCGCCGACGAGCTGCCGGCGGTGGACATGTTCGTGACGACGGCGGACCCGAAGCTGGAGCCACCGGTGGTGACGGTGAACACGGTGCTGTCGCTGATGGCGCTGGACTACCCCGCGGGCAAGCTGACGTGCTACGTCTCCGACGACGGCTGCTCGGCGGTGACCTGCTACGCGCTGCGCGAGGCCGCCGAGTTCGCCAAGCTCTGGGTGCCGTTCTGCAAGAAGCACGGCGTCGGGGTCCGGGCCCCCTTCGTGTACTTCTCCGGCGGCGGCACGGCGGAGCGCGGTGGCGCCACCACCGACGACGTTGCCGAGTTCATGCGCGCCTGGACGTCCATGAAGAACGAGTACGAGGAGTTGGTCCACCGGATCGAGAACGCCGAGGAGGAGTCTCTAGTCCGGCGCGGCGACGGCGAGTTCGCCGAGTTCGTGGGTGCTGACCGCAGGAACCACCCGACCATAATCAAGGTGCTGTCGGACAATCAGGATGCTGCAGGCGATGGAATCCCAAGCCTCATATACGTCTCGAGGGAGAAGAGCCCCACACAGCCCCACCACTTCAAGGCCGGCGCCATGAACGTCCTGACGAGGGTGTCCGGCGTGGTGACCAACGCTCCCATCGTGCTAAACGTGGACTGCGACATGTTCGCCAACAACCCGCAGGTGGCACTCCACGCCATGTGCCTGCTCATGGGCTTCGACGACGACGTCCACAGCGGCTTCGTCCAGGTGCCACAGAAGTTCTACGGCGCCCTCAAGGACGATCCTTTTGGCAACCAGATGCAGGTTATGTTCGAGAAAATTGGATACGGAGTTGCCGGACTTCAAGGCATATATTACTGTGGGACGGGTTGCTTTCACCGTAGGAAAGTCATGTATGGCGTGCCACCATACCACGCCACCACCAGCAGCAGCAGCATGAAAGATTCACCATCTTACAAGGAGCTACAGAACAGGTTCGGCAGATCGAATGAACTGATCGAGTCGGCGAGGAGCATCATCTCCGGGGACATGTTCAAAGCACCAACGACTCTGGTGGCAGACCTAACGAGTCGCATCGAAGCAGCGAAACAAGTTTCTGCCTGCCGCTACGAAACTGGCACAAGCTGGGGCCAGGAGGCAGGCTGTTGGCATGGCCATGAAGGAAGGGCTATCGGAGGTGAGGAGAAGAGAGCCCTGCCGCCGCCCGATGTGCTAACCAACACTGCCACAACCACCGCCAGCCACCTCGTCAACCTGCCGAGGCATGCTGGATCTAGCCACCAAGGCTCCGGATCCGGCTACCCAGGCCCCGACCAACACTGCGCATCCACGCCGCCGGCTGGAGGTGACCCGCCAGCGTTCCTCGGCGGCGCGCCCACCGGCGGACCAGCCAGCCTCACCCAGTACAAGAGGTGGGCAACAGGCCTGCTGGAGATACTCCTCAGCCGCCATAACCCATGCCTTGTCTCTGCGTTCAAGCGCCTCGATTTCCGGCAGTGCGTCGCCTACCTGGTCATCGACGTGTGGCCCGTCAGGGCACCTTTTGAGCTGTGCTACGCCCTGCTAGGACCTTACTGCCTCATCGCAAACCACTCCTTCCTGCCAAAGGCGTCAGAGCCGAGTTTCCTCATCCCACTGGCCCTCTTCCTGGGCTACAACGCGTACAACCTGGGCGAGTACAAGGACTGCCGGCTCTCGGCGCGTGCCTGGTGGAACAACCACAGGATGCAGCGGATCGTGTCGTCCTCCGCCTGGCTGCTCGCCTTCCTCACCGTCGTCCTCAAGATGCTGGGCCTCTCGGAGACGGTGTTCGAGGTGACACGCAAAGAGCAGCAGAGCTCGTCCGACGGAGGCGCCGGCGACGGCGCGGACCCAGCAGGGCGGTTCACCTTTGACTCGTCGCCAGTGTTCGTCCCACCGACAGCGCTCACGGTGCTGAGCATCGTCGCCATCGCCGTCGGGGCGTGGAGGGCGGTGGTCGCCGGTGCGGTGGAAGGCCGCGTGCCCACCACCGGTGGTGGCCCGGGCGTCGGGGAGCTCGCGTGCTGCGTCTGGCTGGTGCTCTGCTTCTGGCCGTTCGTGAGGGGGCTCGTCGCCGTCGGCAGGGGAAGCTACGGCATCCCCTGGAGCGTCAGGCTCAAGGCGGCTCTGCTCGTCGCCGCGTTCGTGCACTTGTCCACACGCAAC

>Sb03g047220

ATGGAGGATGGCGAGAAGAAGGATTCGCAAGCGCCACCACCACCGCTGAGCGCGGTGCACGTGAACAGGCCCCTGGTCGCCGCCAACCGCGCCATGGCCGCCGTCCATGCCGCGCTCGTCGCCGCGGTCATCGCGCACCGCGTCCTGGCGCTGCTCTCAGTCTCAGTCGTCTCCCGGCACGTGGACGTGGCCATGGCGCTGGCCGACCTGACGCTGCTGTTCCTGTGGACGCTGTCGCAGTCGGGGCTGTGGCGTCCCGTCACGCGCGCGGCGTTCCCGGACCGGCTGCTGGCGGCGGTGCCCCGCGACGCGCTGCCGCGCGTGGACGTGCTGGTGGTGACGGCCGACCCGGACAAGGAGCCGCCGCTCGGTGTGATGAACACGGTGGTGTCGGCCATGGCGCTGGACTACCCCGGCGCGGCGCTCAGCGTGTACCTGTCGGACGACGCCGGGTCGCCGCTCACGCTGCTCGCCGCCAGAAAGGCCTACGCCTTCGCCAGGGCCTGGGTGCCCTTCTGCAGGAGGCACTCCGTGCGGTGTCCCTGGCCCGACAGGTACTTCGCCGGCGACGACGACGCTCACGGCGGCCGGGAGGAGCTCGCCGAGGAGAGGGCAAGAGTCAAGAAATTATACGAGAAGTTGAAAGCGGACATAGAGGCGGCCAACAAGGACGACAATATTTCCGGGAGCTGGACAAAAGCAGAACGCCAGGATCACGATGCTTACGTGGAGATCATCAGTGGTAAGGAAGACGGCGACGAGGAGGAGGAGATGCCGCCGGCGCTGGTGTACGTAGCCCGCGAGAAGCGAAGGGCGTGGCCTGACCACTTCAAGGCCGGCGCTCTCAACGCCCTGCTGCGAGTGTCGGGCGTGGTGAGCAACGCGCCCTACGTGCTGGTCCTCGACTGCGACATGGCCTGCAACAGCCGCGCCTCCGCCATGGACGCCATGTGCTTCCTCCTCCTCGACGACCGCCGCAGCCCGCCGACTAACCTCGCCTTCGTGCAGTTCCCGCAGATGTTCCACAACCTCAGCCACAACGACATCTACACCAACGAGCTCAGATACATCTTTGGGACCCGATGGTTCGGCCTGGACGGCGTCCGGGGCCCTTTCCTCTCCGGCACCGGCTTCTACGTCAGGAGAGACGCGCTGTACGGGGCCACGCCGCCGCCCGGGAGCACAGACTTGAGCTCCATGGATGCCGGAGACCTCAAGGCAAGGTTCGGCCACTCCGACCGTCTCGTGGCATCCTTACGCGGCGGCGGCGATGACCAGCGTCGTCGTCGTCGTCTCCCCCCAGAGCCAGTAGAATCCCTGGTGGCCACCTGCGCGTACGAGGCGGGCACCGCCTGGGGCACCGGCGTCGGCTTCATGTACCAGTCGGTGGTGGAGGACTACTTCACTGGGTACCAGCGCTTCTTCTCGCGGGGGTGGACGTCCGCCTACTGCTACCCGGAGCCCCGGCCGGCGTTCCTCGGCAGCGTGCCCACCAACCTCAACGACGTGCTGGTCCAGAACAAGCGCTGGATGTCCGGGATGCTCGCCGTCGGCGTGTCCAGGCACCACAGCCCGCTCGCCTGCCGCCCGCTCCTCAGGGCCTCCCTGCTCCAGGCCATGGGCTACGCCTACTTCGGCTTCGCCGCGCTCTACGCCGTCCCCGTGCTCTGCTACGCCACCCTGCCGCAGCTCTGCCTCCTCCACGGCGTCCCGCTCTTCCCCTGCCCCGCCGCCGCCGCAGCGGCGTTCGCGTCCTCGCTGCTGCTGCACCTGGCGGAGGTGTGCGTGGCCAGGCGTGGGAGGATGGACCTGCGCACGTGGTGGAACGAGCAGAGGTTCTGGGTGCTCAACGCCCTCACCGGCCAGCTCTTGGGCTGCGTCAGCGCCGCCCAGGAGCTGCTCGGCGCGCGCGCCTTGGACTTCGACCTCACCACCAAGGCCGCCGACGCCGACGGGAGGCTGTACCAGGACGGCGTCTTCGACTTCACGGGCTGCTCCACCCTGCTGCTACCTGCCACCACGCTCTCCGTGCTCAACGCCGCCGCCATCGTCGCGGGAACCTGGAAGATGACGTTCCAATTCGCCGGCGAGCTGCTCCCGCAGCTGTTTCTCATGTGCTACGGCGCGGCGCTCAGCTACCCGCTGCTGGAGGGGATGTTCCTCCGGTGGGATGCTGCCAGGGTTCCGCCACGCATCACCGCACTGTCGGTCGCCTCGGCAGCCGTGCTGCTCGCCGTGTTTGGA
